# Development of Biarylalkyl Carboxylic Acid Amides with Improved Anti‐schistosomal Activity

**DOI:** 10.1002/cmdc.201900423

**Published:** 2019-09-18

**Authors:** Alejandra M. Peter Ventura, Simone Haeberlein, Kerstin Lange‐Grünweller, Arnold Grünweller, Roland K. Hartmann, Christoph G. Grevelding, Martin Schlitzer

**Affiliations:** ^1^ Department of Pharmaceutical Chemistry Philipps Universität Marburg Marbacher Weg 6 35032 Marburg Germany; ^2^ BFS, Institute of Parasitology Justus-Liebig-Universität Gießen Schubertstrasse 81 35392 Gießen Germany

**Keywords:** biaryls, carboxamides, inhibitors, schistosomiasis, structure–activity relationships

## Abstract

The parasitic disease schistosomiasis is the cause of more than 200 000 human deaths per year. Although the disease is treatable, there is one major shortcoming: praziquantel has been the only drug used to combat these parasites since 1977. The risk of the emergence of resistant schistosomes is known to be increasing, as a reduced sensitivity of these parasites toward praziquantel has been observed. We developed a new class of substances, which are derived from inhibitors of human aldose reductase, and which showed promising activity against *Schistosoma mansoni* couples in vitro. Further optimisation of the compounds led to an increase in anti‐schistosomal activity with observed phenotypes such as reduced egg production, vitality, and motility as well as tegumental damage and gut dilatation. Here, we performed structure–activity relationship studies on the carboxylic acid moiety of biarylalkyl carboxylic acids. Out of 82 carboxylic acid amides, we identified 10 compounds that are active against *S. mansoni* at 25 μm. The best five compounds showed an anti‐schistosomal activity up to 10 μm and induced severe phenotypes. Cytotoxicity tests in human cell lines showed that two derivatives had no cytotoxicity at 50 or 100 μm. These compounds are promising candidates for further optimisation toward the new anti‐schistosomal agents.

## Graphical abstract

**No flukes with these amides**! Schistosomiasis causes more than 200 000 deaths per year and is treated with the single drug praziquantel. We have previously reported biarylalkyl carboxylic acid derivatives as novel chemical entities with in vitro anti‐schistosomal activity. In continuation of these studies, we report here a series of amide derivatives. The best results were obtained with morpholine, thiomorpholine and substituted piperazine derivatives.

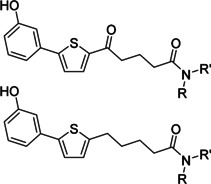

## Introduction

In recent years, the neglected tropical disease schistosomiasis has gained attention as more than 206 million people were infected in 2016, and 800 million people are at risk of being infected.[^1^, ^2^, ^3^] This disease is caused by parasitic flatworms of the genus *Schistosoma*, which are characterised by their sexual dimorphism. Female and male worms pair once they are fully developed. Depending on the species, each pair produces between 300 and approximately 3000 eggs per day.^[4]^ These eggs either remain within the host and cause the symptoms of the disease, or are excreted into the environment. Upon exposure to fresh water, miracidia hatch from the eggs and seek an intermediate host (fresh water snails), in which they develop into cercariae, the infectious stage of the parasite′s life cycle. After penetrating the final host, cercariae develop into schistosomula (juvenile stage) and later into adult worms.[^2^, ^5^] With respect to their relevance for human health, three species are most common: *S. mansoni*, *S. japonicum* and *S. haematobium*. All three species localise in different parts of the body but cause similar symptoms.^[5]^ If not released in feces (*S. mansoni*, *S. japonicum*) or urine (*S. haematobium*) into the environment, remaining eggs get trapped in host tissues, such as liver and spleen, causing severe inflammation, periportal fibrosis, anaemia, and/or haematuria.^[6]^

**Figure 1:**
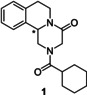
Structure of the anti‐schistosomal drug praziquantel (PZQ).

Praziquantel (PZQ) is the only drug that is widely used against all schistosome species (Figure 1).^[2]^ An advantage of this drug is its efficacy toward all schistosome species and its low cost for treatment.^[7]^ If 60 mg kg^−1^ of PZQ is administered orally on three consecutive days, a 90 % cure rate can be achieved.^[8]^ Notably, the drug mainly affects adult worms but not the juvenile stage.^[9]^ As this drug has been used since the 1970s, the hazard of emerging resistance is growing. Furthermore, a lower sensitivity of schistosomes toward this drug has been reported.[^10^, ^11^] Therefore, the development of new potential anti‐schistosomal agents is urgently needed.^[2]^

**Figure 2:**
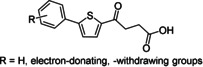
Inhibitors of human aldose reductase that were initially tested on adult *S. mansoni* couples.^[17]^

The schistosomal aldose reductase (AR) is thought to play an important role in antioxidant pathways and protection of the worms from attack by its host's reactive oxygen species.[^12^, ^13^] This makes schistosomal AR an interesting target for novel anti‐schistosomal inhibitors. Furthermore, one orthologue of AR has been identified in the genome of *S*. *mansoni* (Smp_053220).[^14^, ^15^] Therefore, inhibitors of human AR were tested against *S. mansoni* couples in our previous studies (Figure 2).[^16^, ^17^]

**Figure 3:**
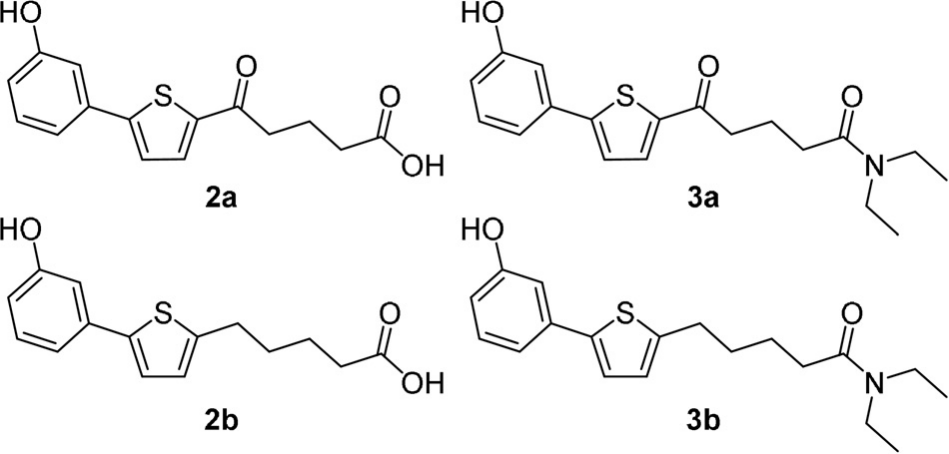
The best biarylalkyl carboxylic acid derivatives from previous studies.^[17]^

Variation of the R substituent of the terminal phenyl moiety showed that structures without electron‐withdrawing substituents had anti‐schistosomal activity.^[17]^ This is in contrast to inhibitors of human AR for which a NO_2_ group conferred best activity.^[16]^ Furthermore, good anti‐schistosomal activity was observed with the diethylamido compounds **3 a** and **3 b** (Figure 3). Because most AR inhibitors have an acidic functionality, AR appears unlikely as a potential target structure.[^17^, ^18^, ^19^] Nevertheless, we chose structures **3 a** and **3 b** (Figure 3) as our starting point for further structure–activity relationship (SAR) studies toward novel anti‐schistosomal agents, in which the N‐substituents were varied.

## Results and Discussion

### Chemistry

**Scheme 1:**
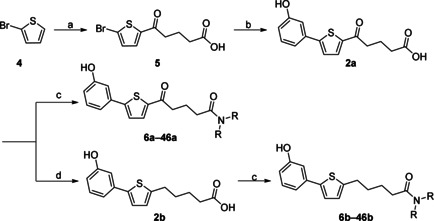
Modified synthesis of the biarylalkyl carboxylic acid derivatives: a) glutaric anhydride, AlCl_3_ in CH_2_Cl_2_, 0 °C to RT, overnight; b) 3‐hydroxyphenylboronic acid, K_3_PO_4_, Pd(OAc)_2_, EtOH/H_2_O, RT, overnight; c) amine, HOBt, EDC**⋅**HCl, NEt_3_, CH_2_Cl_2_, 0 °C to RT, overnight; d) hydrazine monohydrate, KOH, diethylene glycol, 180 °C, overnight.

The biarylalkyl carboxylic acid amides were synthesised in three steps (Scheme 1). In contrast to our previous procedures,[^16^, ^17^] we modified the synthesis by changing the reaction sequence. Consequently, a higher yield was obtained for the previously described biarylalkyl carboxylic acid **2 a**. First, a Friedel–Crafts acylation with 2‐bromothiophene (**4**) and glutaric anhydride was performed, affording precursor **5**. This was subsequently used in a Suzuki reaction, which afforded the previously described carboxylic acid **2 a** in a total yield of 77 % over both steps. The final step was coupling of **2 a** with the appropriate amines to yield the desired carboxylic amides **6 a**–**46 a**.

In addition, the carboxylic acid amides were synthesised without the carbonyl group in the linker to compare their biological activity. This was achieved by one additional step: the reduction of **2 a** using a Wolff–Kishner protocol leading to the carboxylic acid **2 b**. Subsequent coupling with the appropriate amine afforded the desired products **6 b**–**46 b**.

**Scheme 2:**
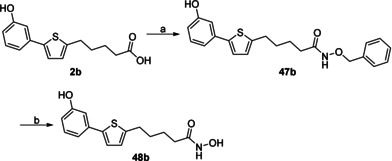
Synthesis of the hydroxamic acid derivative **48 b**: a) 1. oxalylchloride, DMF, CH_2_Cl_2_, RT, 3 h; 2. *O*‐benzylhydroxylamine, NEt_3_, CH_2_Cl_2_, RT, overnight; b) Pd/C, H_2_ atmosphere, MeOH, overnight.

The synthesis of the hydroxamic acid **48 b** was achieved by in situ activation of the carboxylic acid moiety as an acid chloride and coupling with *O*‐benzylhydroxylamine to afford **47 b** (Scheme 2). After the removal of the benzyl protecting group, the desired product **48 b** was obtained.

### Biological evaluation and structure–activity relationships

The anti‐schistosomal activity was determined using an established assay.[^17^, ^20^, ^21^] Ten adult *S. mansoni* couples were exposed to the respective compound in the medium at an initial concentration of 25 μm and analysed by bright‐field microscopy every 24 h for a total time of 72 h. Phenotypes such as worm motility, pairing stability, egg production, tegumental damage, and morphological aberrations including gut dilatation were observed. For negative and positive controls, worms were treated either with DMSO or PZQ, respectively. Furthermore, the cytotoxicity of the most active compounds was assessed by measuring the proliferation of HepG2 and LS174T cells according to the instructions of the manufacturer (Roche, Mannheim, Germany, WST assay).^[22]^

The compounds used as references in previous studies,^[17]^ **2 a/b** and **3 a/b** (Figure 3), were selected in order to compare the extent of the phenotypes to those of the newly synthesised compounds. Unexpectedly, the previous results with these compounds could not be completely reproduced. Phenotypes obtained with **2 a/b** and **3 a/b** did not occur to the same extent or at similar concentrations as before. Whereas a decrease in egg production was again found for **3 b**, on this occasion, other effects were not observed with these compounds. Because different sources of the added in vitro culture supplements were used compared to the previous study (newborn calf serum, HEPES and antibiotic–antimycotic; see the Experimental Section), we tested their influence. A slight influence was observed on egg production, which was more affected by these compounds if the supplements were missing (data not shown). Other parameters such as vitality, motility, and pairing stability were unaffected. Thus, it is unclear why the results with **2 a/b** and **3 a/b** differed from the former study.

Derivatisation of the original compounds began by varying the alkyl groups at the amide nitrogen (Table 1). Lengthening the dialkyl chains of the amide led to a significant reduction of egg production (by 90 % to 98 %) after 72 h at 25 μm (Table 1). Microscopic analysis of the worms revealed no effect on motility or morphological phenotypes. Interestingly, we observed a higher decrease in egg production with compounds lacking the keto group except for the dibutylamides **8 a** and **8 b**. The best result was obtained with **9 b**, leading to a 98 % reduction in egg numbers after 72 h. These results led to the assumption that the keto group within the alkyl chain is not necessary to reduce the numbers of eggs produced. In contrast to the N,N‐disubstituted amides, N‐monosubstituted amides showed no significant activity on egg production. In this respect, hydroxamic acid derivatives **47 b** and **48 b** were also inactive.

**Table 1:** Alkyl amides and phenotype observation.

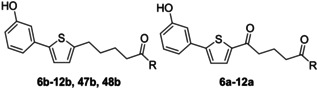			
Compd	R	Activity [μm]^[a]^	Reduction in egg number
**6 a**	N(CH_3_)_2_	n.a.	–
**7 a**	N(C_3_H_7_)_2_	n.a.	–
**7 b**		25	90 %
**8 a**	N(C_4_H_9_)_2_	25	96 %
**8 b**		25	90 %
**9 a**	N(C_5_H_11_)_2_	25	90 %
**9 b**		25	98 %
**47 b**	NHOBzl	n.a.	–
**48 b**	NHOH	n.a.	–
**10 a**	NHC_2_H_5_	n.a.	–
**10 b**		n.a.	–
**11 a**	NHC_4_H_9_	n.a.	–
**11 b**		25	81 %
**12 a**	NCH_3_Bzl	n.a.	–
**12 b**		n.a.	–

[a] Activity measured at 25 μm, values were determined in single experiments, and are the lowest concentration at which an effect on egg production was observed; n.a.=not active. Bzl=benzyl. *n*=1.

**Figure 4:**
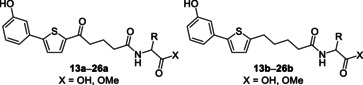
General structures of the amino acid derivatives **13 a**–**26 a** and **13 b**–**26 b**.

Glucose transporters and the amino acid transporter SPRM1lc have been discovered in the tegument of schistosomes.[^23^, ^24^, ^25^, ^26^] This indicates that nutrients such as glucose and amino acids can be taken up by the schistosomes not only through their mouth but also through their tegument.[^23^, ^24^, ^25^, ^26^] Therefore, we coupled amino acids (Figure 4, **13 a**–**26 a** and **13 b**–**26 b**) with the carboxyl moiety to use them as “trojan horses”, possibly favouring the uptake of the compounds by schistosomes. As summarised in Table S1 (Supporting Information), this approach failed to provide the desired results. None of the amino acid derivatives showed anti‐schistosomal activity at a concentration of 25 μm after 72 h.

By variation of the amide dialkyl chain length, different compounds with an inhibitory effect on egg production were found. However, no effects on vitality, motility and pairing stability of the adult *S. mansoni* couples were found using this series. Mono‐substituted amides and amino acids showed no activity at 25 μm.

**Figure 5:**
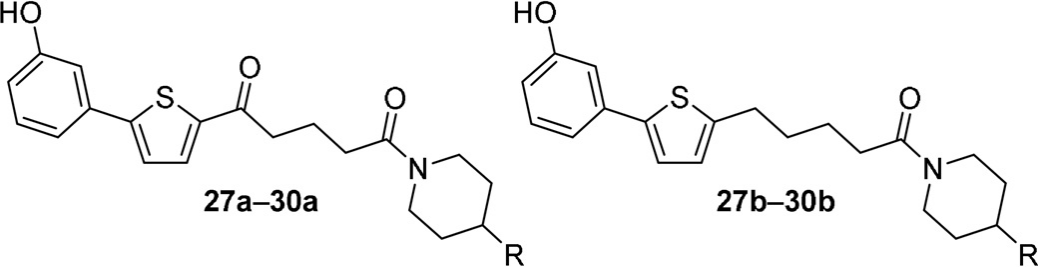
General structures of the piperidine derivatives **27 a**–**30 a** and **27 b**–**30 b**.

To increase rigidity of the amide, piperidine derivatives (Figure 5, **27 a**–**30 a** and **27 b**–**30 b**) were synthesised. This variation also provided no apparent anti‐schistosomal activity at 25 μm (Table S2).

**Figure 6:**
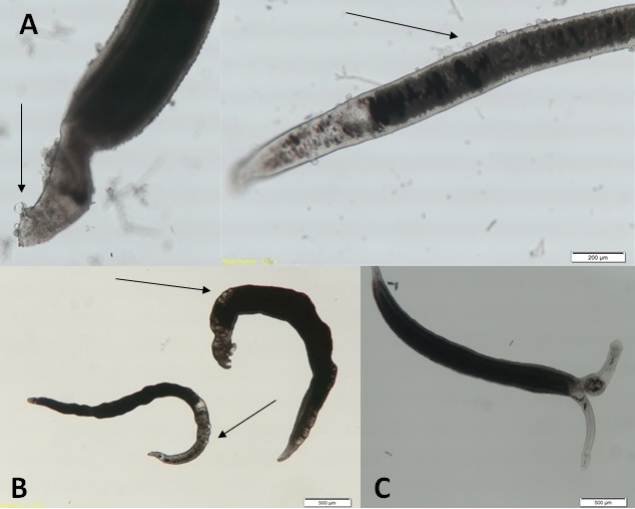
Photographs of *S. mansoni* worms after 72 h treatment with the following compounds (25 μm): A) **46 b**, arrows show tegumental damage; B) **39 b**, arrows show gut dilatation; C) control, untreated *S. mansoni* couple.

In summary, dialkylamines showed negligible activity, as did mono‐substituted alkylamines, amino acids, and piperidine derivatives. Next, we examined whether an additional heteroatom in the piperidine moiety would increase activity (Table 2). After treating the worms with such compounds for 72 h at 25 μm, we observed remarkable reduction in egg production. In addition, we observed separation of worm couples, reduced motility, and tegumental damage (Figure 6 A) and/or severe gut dilatation (Figure 6 B) for most derivatives.

**Table 2:** Phenotype observation for piperazine derivatives.

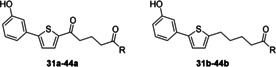			
Compd	R	Activity [μm]^[a]^	Phenotypes
**31 a**	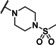	n.a.	–
**31 b**		25	100 % e.r., 100 % c.s., teg. dam. ♀
**32 a**	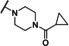	n.a.	–
**32 b**		n.a.	–
**33 a**	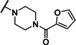	n.a.	–
**33 b**		n.a.	–
**34 a**	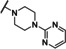	n.a.	–
**34 b**		n.a.	–
**35 a**	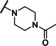	n.a.	–
**35 b**		n.a.	–
**36 a**	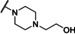	n.a.	–
**36 b**		n.a.	–
**37 a**	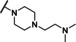	n.a.	–
**37 b**		n.a.	–
**38 a**	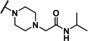	n.a.	–
**38 b**		n.a.	–
**39 a**	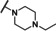	25	99 % e.r., 90 % c.s., gut dil. ♀ and ♂
**39 b**		25	85 % e.r.
**40 a**	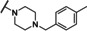	25	100 % e.r., 80 % c.s., gut dil. ♀
**40 b**		25	97 % e.r., gut dil. ♀
**41 a**	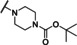	n.a.	–
**41 b**		25	100 % e.r., 70 % c.s., gut dil. ♀
**42 a**	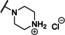	n.a.	–
**42 b**		25	100 % e.r, 90 % c.s, gut dil. ♀ and ♂
**43 a**	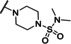	n.a.	–
**43 b**		n.a.	–
**44 a**	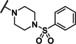	n.a.	–
**44 b**		25	100 % e.r., 100 % c.s., teg. dam. ♀ and ♂, gut dil. ♀

[a] Activity measured at 25 μm; values are the lowest concentration at which anti‐schistosomal activity was observed. n.a.=not active at 25 μm; e.r.=egg number reduction; c.s.=couple separation; gut dil.=gut dilatation; teg. dam.=tegumental damage. *n*=3 for active compounds.

Treating worms with the phenylsulfonyl piperazine compound **44 b** provoked gut dilatation in the female and tegumental damage of both female and male worms. The Boc‐protected piperazine derivative **41 b** and the deprotected piperazine analogue **42 b** showed similar phenotypes at 25 μm. Interestingly, these phenotypes were observed with the Boc‐protected **41 b** after prolonged treatment. This might be explained by the Boc‐protected piperazine acting as a prodrug being slowly transformed into its active amine form.

Because the compound containing an unprotected piperazine moiety (**42 b**) showed promising results, we exchanged the distal nitrogen atom with an oxygen or sulfur atom that is, exchanging piperazine for morpholine and thiomorpholine groups, respectively (Table 3). These modifications resulted in active compounds. Both the morpholine (**45 b**) and thiomorpholine (**46 b**) derivatives without the carbonyl linker were active at 25 μm. Treatment of worms with the morpholine derivative **45 b** at 25 μm led to a 98 % inhibition of egg production and 40 % decreased pairing stability. Also, at 10 μm, this compound was also active although the phenotypes were of lower intensity.

**Table 3:** Phenotype observation for morpholine and thiomorpholine derivatives.

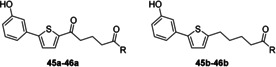			
Compd	R	Activity [μm]^[a]^	Phenotypes
**45 a**	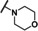	n.a.	–
**45 b**		10	73 % e.r., 50 % c.s.
**46 a**	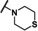	n.a.	–
**46 b**		25	100 % e.r., 100 % c.s., teg. dam.

[a] Activity measured at 25 μm; values are the lowest concentration at which anti‐schistosomal activity was observed. n.a.=not active. e.r.=egg number reduction; c.s.=couple separation; gut dil.=gut dilatation; teg. dam.=tegumental damage. *n*=3.

Compound **46 b** was active at a concentration of 25 μm. The worms showed the same phenotypes as those treated with **45 b**. In addition, we observed tegumental damage (Figure 6 A) of both male and female worms. Notably, only the compounds without the carbonyl group in the linker exhibited anti‐schistosomal activity. This result is consistent with the other results in this series. We assume that the carbonyl group in the linker might not be required, and it even lowers the anti‐schistosomal effects.

Of all the prepared series, 10 compounds active at 10 and/or 25 μm were obtained. The six most active compounds (**39 a**, **31 b**, **42 b**, **44 b**, **45 b** and **46 b**) were further tested for their cytotoxicity in HepG2 and LS174T cells (Table 4). Importantly, two compounds (**31 b** and **45 b**) showed no cytotoxicity at 100 μm in either cell line. Compound **39 a** was cytotoxic only at 100 μm but not at 50 μm in both cell lines. Interestingly, **44 b** had no cytotoxicity at 100 μm in LS174T cells, however it was cytotoxic at 50 μm in HepG2 cells. Based on these results, **31 b** and **45 b** were selected for further evaluation as the most promising candidates.

**Table 4:** Cytotoxicity of the top six compounds in HepG2 and LS174T cells.

Compd	Activity [μm]^[a]^	Cytotoxicity	
		HepG2	LS174T
**39 a**	25	50<*x*<100	50≤*x*<100
**31 b**	25	>100	≥100
**44 b**	25	<50	≥100
**42 b**	25	<50	n.d.
**45 b**	10	>100	≥100
**46 b**	25	<50	n.d.

[a] Activity measured at 25 μm; values are the lowest concentration at which anti‐schistosomal activity was observed. n.d.=not determined.

## Conclusions

Schistosomiasis represents a worldwide threat for humans and animals. Lowered PZQ sensitivity in treated patients has been observed, provoking the fear of future resistance,[^10^, ^11^, ^27^, ^28^] therefore alternative medications are needed. A new approach originating from potential inhibitors of human AR revealed promising anti‐schistosomal compounds. Our results indicate that the carboxylic acid amides possess higher activity than the corresponding free carboxylic acid. Because the carboxylic acid is an important moiety for inhibiting the human AR,^[29]^ we think that the schistosome AR might not be the target for these compounds. Evidently, the keto carbonyl group is not essential for anti‐schistosomal activity, as its removal led to a higher activity in most cases. Out of 82 carboxylic acid amides, we identified the best six, which showed activity as low as 10 μm. These six compounds were tested for their cytotoxicity in two cell lines (HepG2 and LS174T). The results showed that two compounds were non‐cytotoxic at concentrations of up to 100 μm in both cell lines. Therefore, these two compounds, the morpholine derivative **45 b** and the methylsulfonyl piperazine derivative **31 b**, are the most interesting starting points for further optimisation. Selected compounds will be assayed against other pathogens such as *S. japonicum* and *Fasciola hepatica*. In addition, the most active compounds will be evaluated for their DMPK data to facilitate subsequent in vivo testing. Furthermore, additional SAR studies will be performed, for example, variation of the thiophene ring.

## Experimental Section

### Biological methods

**In vitro assay on** ***S. mansoni*** **couples**: Animal experiments were approved by the Regierungspräsidium (regional council) Giessen (V54‐19 c20/15 cGI18/10) and were performed in accordance with the European Convention for the Protection of Vertebrate Animals guidelines on the use of animals for experimental and other scientific purposes (ETS No. 123; revised Appendix A).

Forty‐six days after infection of Syrian hamsters (*Mesocricetus auratus*) with cercariae, *S. mansoni* couples were obtained by hepatoportal perfusion and incubated in groups of 20 couples in 60 mm diameter Petri dishes (Greiner Bio‐One) with M199 medium (Gibco) supplemented with 10 % newborn calf serum (NCS, Sigma), 1 % HEPES (1 m, Roth), and 1 % antibiotic–antimycotic (ABAM, GE Healthcare; 10 000 units penicillin, 10 mg streptomycin, and 25 mg amphotericin B per mL) at 37 °C with 5 % CO_2_. Twenty‐four hours after the perfusion, in vitro culture experiments were started. Six‐well plates (Greiner Bio‐One) were filled with 10 couples per well and a total volume of 5 mL (medium+compound). All substances were dissolved as 10 mm stock solutions in DMSO (Sigma–Aldrich). The substances were initially tested at 25 μm. Therefore, the latter were dissolved in DMSO. If a positive result was achieved, the experiment was repeated at a lower concentration of that substance (10 μm). DMSO (25 μm) was used in every experiment as the negative control, whereas PZQ (5 μm) served as a positive control. Every 24 h, new plates with medium and substance were prepared. After evaluating the worms for their pairing stability and phenotypes (such as gut peristalsis, vitality and motility) under bright‐field microscopy, the worms were transferred to the newly prepared six‐well plates. Then, the eggs produced during the preceding 24 h were counted. Compounds showing only a reduction in egg production were not regarded as active. Compounds showing at least one phenotype in addition to egg number reduction were regarded as active and re‐tested twice at different days. For active compounds, three independent test experiments were performed.

**Cytotoxicity measurements**: The two cell lines tested, HepG2 (human hepatoma) and LS174T (colon carcinoma), were each cultured in Iscove's modified Dulbecco medium (IMDM), l‐glutamine (5 μm) and 10 % fetal calf serum (FCS) at 37 °C and 5 % CO_2_. A WST‐1 assay was performed for both cell lines. In brief, 1×10^4^ cells in IMDM (200 μL, containing 10 % FCS) were seeded in 96‐well microplates and incubated for 24 h (37 °C, 5 % CO_2_). Afterward, old medium was replaced by fresh medium containing the respective compound (50 or 100 μm), and the cells were incubated for up to 144 h. DMSO‐treated cells served as controls. Samples were measured after 24, 72, 120, and 144 h (*n*=3 for each time point and concentration). After the removal of the medium, 10 % WST‐1 reagent (110 μL in phosphate‐buffered saline; Roche, Mannheim, Germany) was added, and the absorbance at 450 nm was measured after 2 h by using a Tecan Safire II plate reader.

### Chemistry


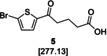
 **5‐(5‐Bromothiophene‐2‐yl)‐5‐oxopentanoic acid (5)**:^[17]^ Under an argon atmosphere, 2‐bromothiophene (**4**, 1.00 g, 6.13 mmol, 1.0 equiv) and glutaric anhydride (0.70 g, 6.13 mmol,1.0 equiv) were dissolved in anhydrous CH_2_Cl_2_ (40 mL) and cooled to 0 °C. After the addition of AlCl_3_ (1.80 g, 13.5 mmol, 2.2 equiv), the reaction mixture was stirred at 0 °C for 5 h and at RT overnight. The reaction was quenched by the addition of 10 % aq HCl (60 mL) and the mixture was stirred at RT for 1 h. The aqueous phase was extracted with CH_2_Cl_2_ and the combined organic layers were washed with 1 m NaOH. The aqueous phase was acidified with concentrated HCl, and the precipitate was filtered, washed with demineralised H_2_O and dried to afford **5** as an orange solid (1.50 g, 5.44 mmol, 89 %), which was used without further purification. ^1^H NMR ([D_6_]DMSO, 400 MHz): *δ*=12.03 (s, 1 H), 7.78 (d, ^3^ *J*=4.1 Hz, 1 H), 7.39 (d, ^3^ *J*=4.0 Hz, 1 H), 2.95 (t, ^3^ *J*=7.2 Hz, 2 H), 2.28 (t, ^3^ *J*=7.6 Hz, 2 H), 1.81 ppm (quin, ^3^ *J*=7.3 Hz, 2 H); ^13^C NMR ([D_6_]DMSO, 100 MHz): *δ*=192.2, 174.2, 145.4, 134.0, 132.4, 121.6, 36.9, 32.7, 19.4 ppm; MS (ESI+): *m*/*z* (%): 277 (10) [*M*+H]^+^, 294 (100) [*M*+NH_4_]^+^; HRMS (ESI−): *m*/*z*: calcd for C_9_H_8_O_3_SBr: 274.9383 [*M*−H]^−^; found: 274.9374.


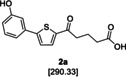
 **5‐[5‐(3‐Hydroxyphenyl)thiophene‐2‐yl]‐5‐oxopentanoic acid (2 a)**:^[17]^ Under an argon atmosphere, **5** (5.38 g, 19.5 mmol, 1.2 equiv), 3‐hydroxyphenylboronic acid (3.23 g, 23.4 mmol, 1.2 equiv) and K_3_PO_4_ (8.28 g, 39.0 mmol 2.0 equiv) were suspended in EtOH/H_2_O (2:1, 100 mL). After 5 min of stirring, Pd(OAc)_2_ (87.6 mg, 0.39 mmol, 2 mol %) was added and the mixture was stirred at RT overnight. Aqueous HCl (1 m) was added and the mixture extracted with EtOAc. The combined organic layers were washed with NaOH (1 m) and the aqueous phase was acidified with concentrated HCl. The precipitate was filtered, washed with H_2_O and EtOH and dried to afford **2 a** as a light‐brown solid (5.00 g, 17.2 mmol, 88 %). ^1^H NMR ([D_6_]DMSO, 400 MHz): *δ*=12.03 (s, 1 H), 9.73 (s, 1 H), 7.91 (d, ^3^ *J*=4.0 Hz, 1 H), 7.55 (d, ^3^ *J*=3.9 Hz, 1 H), 7.26 (t, ^3^ *J*=7.9 Hz, 1 H), 7.19 (ddd, ^3^ *J*=7.7 Hz, ^4^ *J*=1.7, 1.0 Hz, 1 H), 7.11 (t, ^3^ *J*=2.0 Hz, 1 H), 6.81 (ddd, ^3^ *J*=8.1 Hz, ^4^ *J*=2.4, 1.0 Hz, 1 H), 2.99 (t, ^3^ *J*=7.3 Hz, 2 H), 2.31 (t, ^3^ *J*=7.3 Hz, 2 H), 1.85 (quin, ^3^ *J*=7.5 Hz, 2 H); ^13^C NMR ([D_6_]DMSO, 100 MHz): *δ*=192.5, 174.1, 157.9, 151.3, 142.1, 134.2, 133.8, 130.4, 124.8, 116.8, 116.3, 112.6, 37.2, 32.8, 19.6 ppm; MS (ESI+) *m*/*z* (%): 291 (100) [*M*+H]^+^; HRMS (ESI+): *m*/*z*: calcd for C_15_H_15_O_4_S: 291.0686 [*M*+H]^+^; found: 291.0677; calcd for C_15_H_14_O_4_SNa: 313.0505 [*M*+Na]^+^; found: 313.0497.


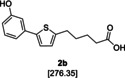
 **5‐[5‐(3‐Hydroxyphenyl)thiophene‐2‐yl]pentanoic acid (2b)**:^[17]^ The keto carboxylic acid **2 a** (2.50 g, 8.61 mmol, 1.00 equiv), hydrazine monohydrate (1.68 mL, 34.4 mmol, 4.00 equiv) and KOH (1.93 g, 34.4 mmol, 4.00 equiv) were suspended in diethylene glycol (35.0 mL) and heated at reflux for 21 h. The reaction was quenched with H_2_O (200 mL) and the aqueous phase was extracted with EtOAc. The aqueous phase was acidified with 10 % aq HCl solution and extracted with EtOAc. The combined organic layers were washed with 1 m NaOH and the aqueous phase was acidified with concentrated HCl. The precipitate was filtered, washed with H_2_O and dried to afford **2 b** as a brown solid (2.10 g, 7.59 mmol, 88 %). ^1^H NMR ([D_6_]DMSO, 400 MHz): *δ*=11.98 (s, 1 H), 9.49 (s, 1 H), 7.22 (d, ^3^ *J*=3.6 Hz, 1 H), 7.17 (t, ^3^ *J*=7.9 Hz, 1 H), 7.01 (d, ^3^ *J*=7.8 Hz, 1 H), 6.95 (s, 1 H), 6.82 (d, ^3^ *J*=3.6 Hz, 1 H), 6.67 (dd, ^3^ *J*=8.5 Hz, ^4^ *J*=1.8 Hz, 1 H), 2.79 (t, ^3^ *J*=7.3 Hz, 2 H), 2.25 (t, ^3^ *J*=7.0 Hz, 2 H), 1.67–1.53 ppm (m, 4 H); ^13^C NMR ([D_6_]DMSO, 100 MHz): *δ*=174.3, 157.8, 144.3, 140.9, 135.1, 130.0, 125.6, 123.0, 115.8, 114.3, 111.7, 33.3, 30.5, 29.1, 23.9 ppm; MS (ESI+): *m*/*z* (%): 277 (75) [*M*+H]^+^, 294 (100) *M*+NH_4_]^+^, 299 (20) [*M*+Na]^+^; HRMS (ESI+): *m*/*z*: calcd for C_15_H_16_O_3_SNa: 299.0712 [*M*+Na]^+^; found: 299.0709.

**General procedure 1A**: **synthesis of carboxylic acid amides**:^[17]^ The carboxylic acid (1.0 equiv), amine (1.0 equiv) and NEt_3_ (3.0 equiv) were dissolved in CH_2_Cl_2_ and stirred at RT for 5 min. The mixture was cooled to 0 °C and EDC⋅HCl (1.5 equiv) and HOBt (1.5 equiv) were added. Then, the mixture was stirred at RT overnight. The solvent was removed under reduced pressure and the crude product purified by column chromatography.

**General procedure 1B**: **synthesis of carboxylic acid amides**: The carboxylic acid (1 equiv) was dissolved in CH_2_Cl_2_. Oxalyl chloride (1.5 equiv) and DMF (cat. amount) were added. The reaction mixture was stirred for 2 h at RT. The volatile compounds were removed under reduced pressure and a mixture of NEt_3_ (1.5 equiv) and amine (1.5 equiv) in CH_2_Cl_2_ was added. The reaction was stirred at RT overnight, diluted with CH_2_Cl_2_, washed with aq HCl (1 m) and saturated NaHCO_3_ solution, and dried over MgSO_4_. The solvent was removed under reduced pressure and the crude product purified by column chromatography.

**General procedure 2A**: **alkaline hydrolysis**:^[30]^ The ester (1.00 equiv) was dissolved or suspended in MeOH and KOH (3.0 equiv) was added. The reaction mixture was stirred overnight at 35 °C. The reaction was quenched by the addition of H_2_O and the aqueous phase was extracted with EtOAc. The aqueous phase was acidified with concentrated HCl and the precipitate was filtered, washed with H_2_O and dried.

**General procedure 2B**: **alkaline hydrolysis**: The ester (1.00 equiv) was dissolved or suspended in EtOH and NaOH (2 m) was added. The reaction mixture was stirred at 100 °C until the reaction was complete (TLC). The workup was performed as described in procedure 2A. If necessary, the compound was purified by column chromatography.

**General procedure 3**: **removal of Boc protecting group**: The Boc‐protected compound and HCl (4 m in 1,4‐dioxane) were dissolved in 1,4‐dioxane and stirred for 3 h at RT. The resulting precipitate was filtered, washed with 1,4‐dioxane and dried.

**General procedure 4**: **synthesis of sulfonamides**:^[31]^ The corresponding amine (1.0 equiv) and NEt_3_ (3.0 equiv) were dissolved or suspended in 1,4‐dioxane and H_2_O. The sulfonyl chloride (1.0 equiv) was added and the reaction mixture was stirred at RT overnight. The solvent was removed under vacuum and the residue was diluted with EtOAc. The organic phase was washed with brine. After removal of the solvent, the crude product was purified by column chromatography.

**General procedure 5**: **removal of benzyl protecting group**: The benzyl‐protected compound (1 equiv) was dissolved in MeOH. Pd/C (10 wt %, 0.1 equiv) was added and the reaction mixture was stirred at RT under a H_2_ atmosphere until the reaction was complete (TLC). The catalyst was filtered off and the solvent evaporated. The crude product was purified by column chromatography.

Further details about the synthesis of these compounds are provided in the Supporting Information.

## Conflict of interest

*The authors declare no conflict of interest*.

## Supporting information

As a service to our authors and readers, this journal provides supporting information supplied by the authors. Such materials are peer reviewed and may be re‐organized for online delivery, but are not copy‐edited or typeset. Technical support issues arising from supporting information (other than missing files) should be addressed to the authors.

Supplementary
