# Supplementary material for: Development of Biarylalkyl Carboxylic Acid Amides with Improved Anti‐schistosomal Activity
Source: ChemMedChem. 2019 Sep 18;14(21):1856–62. doi: 10.1002/cmdc.201900423 (PMC7687077; doi:10.1002/cmdc.201900423)
Supplement: Supplementary file 1 — Supplementary [file CMDC-14-1856-s001.pdf]

CHEMMEDCHEM
Chemistry & Drug Discovery

Supporting Information

Development of Biarylalkyl Carboxylic Acid Amides with Improved Anti-schistosomal Activity

Alejandra M. Peter Ventura,[a] Simone Haeberlein,[b] Kerstin Lange-Grünweller,[a] Arnold Grünweller,[a] Roland K. Hartmann,[a] Christoph G. Grevelding,*[b] and Martin Schlitzer*[a]

cmdc_201900423_sm_miscellaneous_information.pdf

Index of contents

Supplemental Tables: 7

Experimental Section 9

5-(5-(3-Hydroxyphenyl)thiophene-2-yl)-*N*,*N*-dimethyl-5-oxopentaneamide (6a) 9

5-(5-(3-Hydroxyphenyl)thiophene-2-yl)-5-oxo-*N*,*N*-dipropylpentaneamide (7a) 10

5-(5-(3-Hydroxyphenyl)thiophene-2-yl)-*N*,*N*-dipropylpentaneamide (7b) 11

N,N-Dibutyl-5-(5-(3-hydroxyphenyl)thiophene-2-yl)-5-oxopentaneamide (8a) 12

N,*N*-Dibutyl-5-(5-(3-hydroxyphenyl)thiophene-2-yl)pentaneamide (8b) 12

5-(5-(3-Hydroxyphenyl)thiophene-2-yl)-5-oxo-*N,N*-dipentylpentaneamide (9a) 13

5-(5-(3-Hydroxyphenyl)thiophene-2-yl)-*N*,*N*-dipentylpentaneamide (9b) 14

N-(Benzyloxy)-5-(5-(3-hydroxyphenyl)thiophene-2-yl)pentaneamide (47b) 15

N-Hydroxy-5-(5-(3-hydroxyphenyl)thiophene-2-yl)pentaneamide (48b) 16

N-Ethyl-5-(5-(3-hydroxyphenyl)thiophene-2-yl)-5-oxopentaneamide (10a) 17

N-Ethyl-5-(5-(3-hydroxyphenyl)thiophene-2-yl)pentaneamide (10b) 18

N-Butyl-5-(5-(3-hydroxyphenyl)thiophene-2-yl)-5-oxopentaneamide (11a) 19

N-Butyl-5-(5-(3-hydroxyphenyl)thiophene-2-yl)pentaneamide (11b) 20

N-Benzyl-5-(5-(3-hydroxyphenyl)thiophene-2-yl)-*N*-methyl-5-oxopentaneamide (12a) 21

N-Benzyl-5-(5-(3-hydroxyphenyl)thiophene-2-yl)-*N*-methylpentaneamide (12b) 22

Methyl *N*-(5-(5-(3-hydroxyphenyl)thiophene-2-yl)-5-oxopentanoyl)glycinate 22

5-(5-(3-Hydroxyphenyl)thiophene-2-yl)-5-oxopentanoyl)glycine (14a) 24

Methyl *N*-(5-(5-(3-hydroxyphenyl)thiophen-2-yl)pentanoyl)glycinate (13b) 24

N-(5-(5-(3-Hydroxyphenyl)thiophene-2-yl)pentanoyl)glycine (14b) 25

Methyl *N*-(5-(5-(3-hydroxyphenyl)thiophene-2-yl)-5-oxopentanoyl)-L-alaninate (15a) 26

N-(5-(5-(3-Hydroxyphenyl)thiophene-2-yl)-5-oxopentanoyl)-L-alanine (16a) 27

N-Methyl (5-(5-(3-hydroxyphenyl)thiophene-2-yl)pentanoyl)-L-alaninate (15b) 28

N-(5-(5-(3-Hydroxyphenyl)thiophene-2-yl)pentanoyl)-L-alanine (16b) 29

Methyl *N*-(5-(5-(3-hydroxyphenyl)thiophene-2-yl)-5-oxopentanoyl)-L-phenylalaninat (17a) 30

N-(5-(5-(3-Hydroxyphenyl)thiophene-2-yl)-5-oxopentanoyl)-L-phenylalanine (18a) 31

N-Methyl (5-(5-(3-hydroxyphenyl)thiophene-2-yl)pentanoyl)-L-phenylalaninate (17b) 32

N-(5-(5-(3-Hydroxyphenyl)thiophene-2-yl)pentanoyl)-L-phenylalanine (18b) 33

Methyl *N*-(5-(5-(3-hydroxyphenyl)thiophene-2-yl)-5-oxopentanoyl)-L-prolinate (19a) 34

N-(5-(5-(3-Hydroxyphenyl)thiophene-2-yl)-5-oxopentanoyl)-L-proline (20a) 35

Methyl *N*-(5-(5-(3-hydroxyphenyl)thiophene-2-yl)pentanoyl)-L-prolinate (19b) 35

N-(5-(5-(3-Hydroxyphenyl)thiophene-2-yl)pentanoyl)-L-proline (20b) 36

4-*tert*-Butyl 1-methyl *N*-(5-(5-(3-hydroxyphenyl)thiophene-2-yl)-5-oxopentanoyl)-L-aspartate (21a) 37

N-(5-(5-(3-Hydroxyphenyl)thiophene-2-yl)-5-oxopentanoyl)-L-aspartic acid (22a) 38

4-*tert*-Butyl 1-methyl *N*-(5-(5-(3-hydroxyphenyl)thiophene-2-yl)pentanoyl)-L-aspartate (21b) 39

N-(5-(5-(3-Hydroxyphenyl)thiophene-2-yl)pentanoyl)-L-aspartate (22b) 40

N-Methyl (5-(5-(3-hydroxyphenyl)thiophene-2-yl)-5-oxopentanoyl)-L-histidinate (23a) 41

Methyl *N*-(5-(5-(3-hydroxyphenyl)thiophene-2-yl)pentanoyl)-L-histidinate (23b) ... 42

N-(5-(5-(3-Hydroxyphenyl)thiophene-2-yl)-5-oxopentanoyl)-L-histidine (24b) 43

Methyl *N*-(5-(5-(3-hydroxyphenyl)thiophene-2-yl)-5-oxopentanoyl)-L-valinate (25a) 43

N-(5-(5-(3-Hydroxyphenyl)thiophene-2-yl)-5-oxopentanoyl)-L-valine (26a) 44

Methyl *N*-(5-(5-(3-hydroxyphenyl)thiophene-2-yl)pentanoyl)-L-valinate (25b) 45

N-(5-(5-(3-Hydroxyphenyl)thiophene-2-yl)pentanoyl)-L-valine (26b) 46

1-(5-(3-Hydroxyphenyl)thiophene-2-yl)-5-(piperidine-1-yl)pentane-1,5-dione (27a) 47

5-(5-(3-Hydroxyphenyl)thiophene-2-yl)-1-(piperidine-1-yl)pentane-1-one (27b).... 48

1-(5-(5-(3-Hydroxyphenyl)thiophene-2-yl)-5-oxopentanoyl)piperidine-4-carboxamide (28a) 49

1-(5-(5-(3-Hydroxyphenyl)thiophene-2-yl)pentanoyl)piperidine-4-carboxamide (28b) 49

Ethyl 1-(5-(5-(3-hydroxyphenyl)thiophene-2-yl)-5-oxopentanoyl)piperidine-4-carboxylate (29a) 50

Ethyl 1-(5-(5-(3-hydroxyphenyl)thiophene-2-yl)pentanoyl)piperidine-4-carboxylate (29b) 51

1-(5-(5-(3-Hydroxyphenyl)thiophene-2-yl)-5-oxopentanoyl)piperidine-4-carboxylic acid (30a) 52

1-(5-(5-(3-Hydroxyphenyl)thiophene-2-yl)pentanoyl)piperidine-4-caboxylic acid (30b) 53

1-(5-(3-Hydroxyphenyl)thiophene-2-yl)-5-(4-(methylsulfonyl)piperazine-1-yl)pentan-1,5-dione (31a) 54

5-(5-(3-Hydroxyphenyl)thiophene-2-yl)-1-(4-(methylsulfonyl)piperazine-1-yl)pentane-1-one (31b) 55

1-(4-(Cyclopropylcarbonyl)piperazine-1-yl)-5-(5-(3-hydroxyphenyl)thiophene-2-yl)pentan-1,5-dione (32a) 56

1-(4-(Cyclopropylcarbonyl)piperazine-1-yl)-5-(5-(3-hydroxyphenyl)thiophene-2-yl)pentan-1-one (32b) 57

1-(4-(2-Furoylpiperazine-1-yl)-5-(5-(3-hydroxyphenyl)thiophene-2-yl)pentane-1,5-dione (33a) 58

1-(4-(2-Furoylpiperazine-1-yl)-5-(5-(3-hydroxyphenyl)thiophene-2-yl)pentan-1-one (33b) 58

1-(5-(3-Hydroxyphenyl)thiophene-2-yl)-5-(4-(pyrimidine-2-yl)piperazine-1-yl)pentane-1,5-dione (34a) 59

5-(5-(3-Hydroxyphenyl)thiophene-2-yl)-1-(4-(pyrimidine-2-yl)piperazine-1-yl)pentane-1-one (34b) 60

1-(4-Acetylpiperazine-1-yl)-5-(5-(3-hydroxyphenyl)thiophene-2-yl)pentane-1,5-dione (35a) ... 61
1-(4-Acetylpiperazine-1-yl)-5-(5-(3-hydroxyphenyl)thiophene-2-yl)pentane-1-one (35b) ... 62
1-(4-(2-Hydroxyethyl)piperazine-1-yl)-5-(5-(3-hydroxyphenyl)thiophene-2-yl)pentane-1,5-dione (36a) ... 63
1-(4-(2-Hydroxyethyl)piperazine-1-yl)-5-(5-(3-hydroxyphenyl)thiophene-2-yl)pentane-1-one (36b) ... 64
2-(4-(5-(5-(3-Hydroxyphenyl)thiophene-2-yl)-5-oxopentanoyl)piperazine-1-yl)-*N*,*N*-dimethylethan-1-ammonium (2,2,2-trifluoroacetate) (37a) ... 65
(2-(4-(5-(5-(3-Hydroxyphenyl)thiophene-2-yl)pentanoyl)piperazin-1-yl) (2,2,2-trifluoroacetate)-*N*,*N*-dimethylethan-1-ammonium (37b) ... 66
2-(4-(5-(5-(3-Hydroxyphenyl)thiophene-2-yl)-5-oxopentanoyl)piperazine-1-yl)-*N*-isopropylacetamide (38a) ... 67
2-(4-(5-(5-(3-Hydroxyphenyl)thiophene-2-yl)pentanoyl)piperazine-1-yl)-N-isopropylacetamid (38b) ... 68
1-(4-Ethylpiperazine-1-yl)-5-(5-(3-hydroxyphenyl)thiophene-2-yl)pentane-1,5-dione (39a) ... 69
1-(4-Ethylpiperazine-1-yl)-5-(5-(3-hydroxyphenyl)thiophene-2-yl)pentane-1-one (39b) ... 69
1-(5-(3-Hydroxyphenyl)thiophene-2-yl)-5-(4-(4-methylbenzyl)piperazine-1-yl)pentane-1,5-dion (40a) ... 70
5-(5-(3-Hydroxyphenyl)thiophene-2-yl)-1-(4-(4-methylbenzyl)piperazine-1-yl)pentane-1-one (40b) ... 71
tert-Butyl 4-(5-(5-(3-hydroxyphenyl)thiophene-2-yl)-5-oxopentanoyl)piperazine-1-carboxylate (41a) ... 72
tert-Butyl 4-(5-(5-(3-hydroxyphenyl)thiophene-2-yl)pentanoyl)piperazine-1-carboxylate (41b) ... 73
4-(5-(5-(3-Hydroxyphenyl)thiophene-2-yl)-5-oxopentanoyl)piperazinium-chloride (42a) ... 74

4-(5-(5-(3-Hydroxyphenyl)thiophene-2-yl)-5-pentanoyl)piperazinium-chloride (42b) 74

tert-Butyl 4-(*N,N*-dimethylsulfamoyl)piperazine-1-carboxylate (49) 75

4-(*N,N*-Dimethylsulfamoyl)piperazinium chloride (50) 76

4-(5-(5-(3-Hydroxyphenyl)thiophene-2-yl)-5-oxopentanoyl)-*N*,*N*-dimethylpiperazine-1-sulfonamide (43a) 76

4-(5-(5-(3-Hydroxyphenyl)thiophene-2-yl)pentanoyl)-*N*,*N*-dimethylpiperazine-1-sulfonamide (43b) 77

tert-Butyl 4-(phenylsulfonyl)piperazin-1-carboxylate (51) 78

4-(Phenylsulfonyl)piperazinium chloride (52) 79

1-(5-(3-Hydroxyphenyl)thiophene-2-yl)-5-(4-(phenylsulfonyl)piperazine-1-yl)pentane-1,5-dione (44a) 79

5-(5-(3-Hydroxyphenyl)thiophene-2-yl)-1-(4-(phenylsulfonyl)piperazine-1-yl)pentane-1-one (44b) 80

1-(5-(3-Hydroxyphenyl)thiophene-2-yl)-5-morpholinopentane-1,5-dione (45a) 81

5-(5-(3-Hydroxyphenyl)thiophene-2-yl)-1-morpholinopentane-1-one (45b) 81

1-(5-(3-Hydroxyphenyl)thiophene-2-yl)-5-thiomorpholinopentane-1,5-dione (46a) 82

5-(5-(3-Hydroxyphenyl)thiophene-2-yl)-1-thiomorpholinopentane-1-one (46b) 83

Supplemental Tables:

Table S1. Amino acid derivatives and phenotypic observation

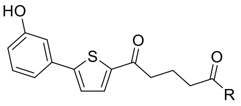

13a-26a

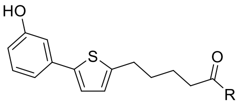

13b-26b

Compound	R	Activity [µM][a]	Phenotypes
**13a**	Gly-OMe	na	-
**13b**		na	-
**14a**	Gly	na	-
**14b**		na	-
**15a**	Ala-OMe	na	-
**15b**		na	-
**16a**	Ala	na	-
**16b**		na	-
**17a**	Phe-OMe	na	-
**17b**		na	-
**18a**	Phe	na	-
**18b**		na	-
**19a**	Pro-OMe	na	-
**19b**		na	-
**20a**	Pro	na	-
**20b**		na	-
**21a**	Asp(OtBu)-OMe	na	-
**21b**		na	-
**22a**	Asp	na	-
**22b**		na	-
**23a**	His-OMe	na	-
**23b**		na	-
**24b**	His	na	-
**25a**	Val-OMe	na	-
**25b**		na	-
**26a**	Val	na	-
**26b**		na	-

[a] activity measured at 25 µM; na = not active. n=1.

Table S2. Piperidine derivatives and phenotypic observation

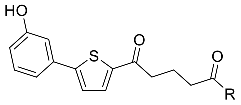

27a-30a

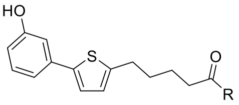

27b-30b

Compound	R	Activity [µM][a]	Phenotypes
**27a**	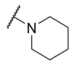	na	-
**27b**		na	-

**28a**	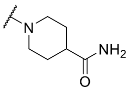	na	-
**28b**		na	-
**29a**	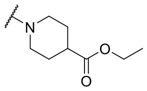	na	-
**29b**		na	-
**30a**	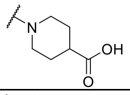	na	-
**30b**		na	-

[a] activity measured at 25 µM; na = not active. n = 1.

Experimental Section

General :

NMR Spectra (^{1}H- and ^{13}C-) were measured with Jeol ECA-400 or ECX-500 spectrometers and the chemical shifts (δ) are shown in ppm relative to the central residual solvent signal. Mass Spectra were measured with a Q-Trap 2000 (Applied Biosystems) and high-resolution mass spectra were recorded with a Micromass VG-Autospec spectrometer. Melting points were identified with a type Mel-Temp® II (*Laboratory Devices Inc., USA*) device. Reagents and solvents were purchased from abcr, Alfa Aesar, Merck, Sigma Aldrich, and Thermo Fisher Scientific. Flash chromatography was performed with the use of Macherey-Nagel silica gel (0.040–0.063 mm) and for analytical thin-layer chromatography (TLC) Merck silica gel 60 F254 plates were used.

5-(5-(3-Hydroxyphenyl)thiophene-2-yl)-*N*,*N*-dimethyl-5-oxopentaneamide (6a)

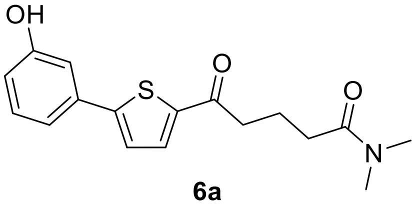

6a
[317.40]

The title compound was synthesised as described in the **general procedure 1A** with **2a** (200 mg, 0.69 mmol, 1.0 eq.), dimethylamine, 11% in iPrOH (3 mL, 0.69 mmol, 1.0 eq.), NEt_3 (0.3 mL, 2.07 mmol, 3.0 eq.), HOBt (140 mg, 1.04 mmol, 1.5 eq.) and EDC•HCl (199 mg, 1.04 mmol, 1.5 eq.) in DCM (50 mL). The crude product was purified *via* column chromatography (DCM:MeOH 20:1). 27.7 mg (0.06 mmol, 13%) of the desired product **6a** as yellow solid were obtained. R_f = 0.19 (DCM:MeOH 20:1). Melting point: 195-197 °C. ^{1}H-NMR: (DMSO-D_6, 400 MHz), δ [ppm] = 9.70 (s, 1H), 7.92 (d, ^3J = 3.9 Hz, 1H), 7.55 (d, ^3J = 4.1 Hz, 1H), 7.26 (t, ^3J = 7.8 Hz, 1H), 7.18 (d, ^3J = 7.8 Hz, 1H), 7.11 (s, 1H), 6.82 (d, ^3J = 8.0 Hz, 1H), 3.00-2.95 (m, 5H), 2.82 (s, 3H), 2.37 (t, ^3J = 7.2 Hz, 2H), 1.84 (quin, ^3J = 7.2 Hz, 2H). ^{13}C-NMR: (DMSO-D_6, 400 MHz), δ [ppm] = 193.0 (1C), 171.5 (1C), 158.0 (1C), 151.3 (1C), 142.2 (1C), 134.3 (1C), 133.9 (1C), 130.4 (1C), 124.9 (1C), 116.9 (1C), 116.3 (1C), 112.6 (1C), 37.6 (1C), 36.6 (1C), 34.8 (1C), 31.6 (1C), 19.9 (1C). IR: ν [cm^{-1}] = 3070 (w), 2935 (w), 2892 (w),

1657 (vs), 1587 (s), 1495 (m), 1438 (vs), 1404 (s), 1340 (s), 1269 (s), 1227 (s), 1206 (s), 1142 (m), 1058 (m), 995 (m), 945 (m), 874 (s), 846 (m), 818 (m), 782 (vs), 754 (s), 691 (m), 642 (w), 620 (w), 584 (w), 493 (m), 479 (m). MS (ESI+): m/z (%) = 318 (80, $[M+H]^+$), 340 (100, $[M+Na]^+$). HRMS (ESI+): calculated for $C_{17}H_{20}NO_3S$: 318.1158; found: 318.1159.

5-(5-(3-Hydroxyphenyl)thiophene-2-yl)-5-oxo-*N*,*N*-dipropylpentaneamide (7a)

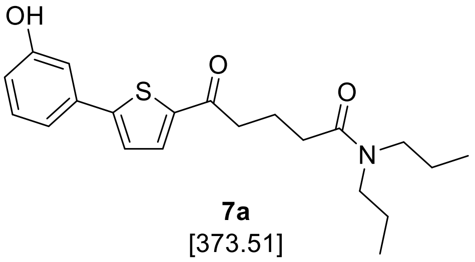

7a
[373.51]

The title compound was synthesised as described in the **general procedure 1A** with **2a** (300 mg, 1.03 mmol, 1.0 eq.), dipropylamine (0.14 mL, 1.03 mmol, 1.0 eq.), NEt_3 (0.43 mL, 3.09 mmol, 3.0 eq.), HOBt (209 mg, 1.55 mmol, 1.5 eq.) and EDC•HCl (297 mg, 1.55 mmol, 1.5 eq.) in DCM (50 mL). The crude product was purified *via* column chromatography (cyclohexane:EtOAc 1:1). 143 mg (0.38 mmol, 37%) of the desired product **7a** as yellow solid were obtained. R_f = 0.20 (cyclohexane:EtOAc 1:1). Smp.: 170-172 °C. ^{1}H-NMR (DMSO-D$_6$, 400 MHz), δ [ppm] = 9.70 (s, 1H), 7.91 (d, ^3J = 3.9 Hz, 1H), 7.55 (d, ^3J = 4.1 Hz, 1H), 7.26 (t, ^3J = 7.9 Hz, 1H), 7.18 (d, ^3J = 7.8 Hz, 1H), 7.10 (s, 1H), 6.83-6.80 (m, 1H), 3.18 (t, ^3J = 7.7 Hz, 4H), 2.98 (t, ^3J = 7.2 Hz, 2H), 2.38 (t, ^3J = 7.2 Hz, 2H), 1.85 (quin, ^3J = 7.3 Hz, 2H), 1.55-1.41 (m, 4H), 0.86-0.79 (m, 6H). ^{13}C-NMR: (DMSO-D$_6$, 400 MHz), δ [ppm] = 193.0 (1C), 171.0 (1C), 158.0 (1C), 151.3 (1C), 142.2 (1C), 134.3 (1C), 133.9 (1C), 130.4 (1C), 124.9 (1C), 116.8 (1C), 116.3 (1C), 112.6 (1C), 48.7 (1C), 46.6 (1C), 37.5 (1C), 31.3 (1C), 20.8 (1C), 20.6 (1C), 20.4 (1C), 11.3 (1C), 11.0 (1C). IR: ν [cm^{-1}] = 3101 (w), 2955 (m), 2878 (w), 1658 (vs), 1591 (s), 1579 (s), 1446 (vs), 1381 (m), 1267 (s), 1230 (s), 1197 (m), 1146 (m), 1082 (m), 938 (m), 865 (s), 777 (s), 750 (vs), 681 (m), 599 (w), 477 (m), 459 (m). MS (ESI+): m/z (%) = 374 (100, $[M+H]^+$). HRMS (ESI+): calculated for $C_{21}H_{28}NO_3S$: 374.1784; found: 374.1788.

5-(5-(3-Hydroxyphenyl)thiophene-2-yl)-*N*,*N*-dipropylpentaneamide (7b)

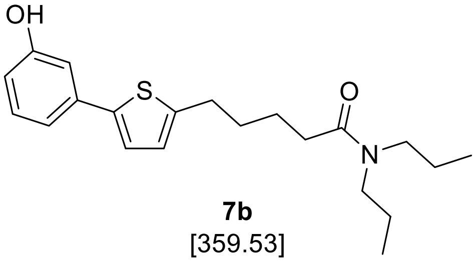

7b
[359.53]

The title compound was synthesised as described in the **general procedure 1A** with **2b** (200 mg, 0.72 mmol, 1.0 eq.), dipropylamine (0.10 mL, 0.72 mmol, 1.0 eq.), NEt_3 (0.30 mL, 2.16 mmol, 3.0 eq.), HOBt (146 mg, 1.08 mmol, 1.5 eq.) and EDC•HCl (208 mg, 1.08 mmol, 1.5 eq.) in DCM (50 mL). The crude product was purified *via* column chromatography (cyclohexane:EtOAc 3:1). 202 mg (0.56 mmol, 78%) of the desired product **7b** as colourless solid were obtained. R_f = 0.20 (cyclohexane:EtOAc 3:1). Melting point: 95 °C. ^{1}H-NMR: (DMSO-D_6, 400 MHz), δ [ppm] = 9.51 (s, 1H), 7.21 (d, ^3J = 3.6 Hz, 1H), 7.17 (t, ^3J = 7.9 Hz, 1H), 7.00 (ddd, ^3J = 7.8 Hz, ^4J = 1.6 Hz, ^4J = 0.9 Hz, 1H), 6.95 (t, ^4J = 3.9 Hz, 1H), 6.81 (d, ^3J = 3.6 Hz, 1H), 6.67 (ddd, ^3J = 8.1 Hz, ^4J = 2.4 Hz, ^4J = 0.9 Hz, 1H), 3.19-3.15 (m, 4H), 2.79 (t, ^3J = 7.1 Hz, 2H), 2.30 (t, ^3J = 7.1 Hz, 2H), 1.68-1.55 (m, 4H), 1.52-1.39 (m, 4H), 0.84 (t, ^3J = 7.4 Hz, 3H), 0.79 (t, ^3J = 7.4 Hz, 3H). ^{13}C-NMR: (DMSO-D_6, 400 MHz), δ [ppm] = 171.3 (1C), 157.8 (1C), 144.6 (1C), 140.9 (1C), 135.2 (1C), 130.7 (1C), 125.6 (1C), 123.1 (1C), 115.8 (1C), 114.3 (1C), 111.7 (1C), 48.7 (1C), 46.6 (1C), 31.8 (1C), 30.7 (1C), 29.4 (1C), 24.5 (1C), 21.9 (1C), 20.6 (1C), 11.3 (1C), 11.1 (1C). IR: v [cm^{-1}] = 3190 (m), 2965 (m), 2953 (m), 2928 (m), 2873 (m), 2836 (m), 1620 (s), 1591 (vs), 1546 (m), 1513 (w), 1475 (m), 1447 (vs), 1412 (m), 1382 (m), 1371 (m), 1320 (m), 1305 (m), 1290 (m), 1268 (m), 1241 (m), 1228 (s), 1216 (m), 1198 (m), 1162 (m), 1147 (m),1113 (m), 1099 (m), 1082 (m), 1073 (m), 1047 (m), 982 (m), 914 (w), 900 (m), 863 (m), 855 (m), 811 (s), 786 (m), 749 (m), 731 (m), 710 (m), 683 (s), 659 (m), 626 (m), 596 (m), 549 (w), 494 (w), 442 (m), 415 (m). MS (ESI+): m/z (%) = 360 (100, $[M+H]^+$), 382 (13, $[M+Na]^+$). HRMS (ESI+): calculated for $C_{21}H_{30}NO_2S$: 360.1992; found: 360.1993.

N,N-Dibutyl-5-(5-(3-hydroxyphenyl)thiophene-2-yl)-5-oxopentaneamide (8a)

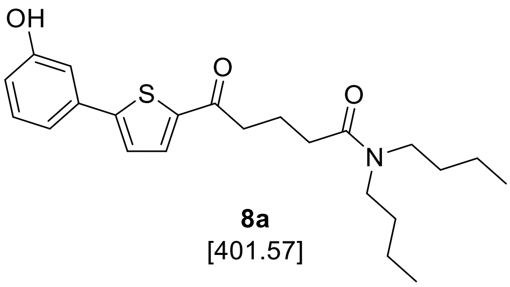

8a
[401.57]

The title compound was synthesised as described in the **general procedure 1A** with **2a** (300 mg, 1.03 mmol, 1.0 eq.), dibutylamine (0.2 mL, 1.03 mmol, 1.0 eq.), NEt_3 (0.43 mL, 3.09 mmol, 3.0 eq.), HOBt (209 mg, 1.55 mmol, 1.5 eq.) and EDC•HCl (297 mg, 1.55 mmol, 1.5 eq.) in DCM (50 mL). The crude product was purified *via* column chromatography (cyclohexane:EtOAc 1:1). 250 mg (0.62 mmol, 60%) of the desired product **8a** as yellow solid were obtained. R_f = 0.28 (cyclohexane:EtOAc 1:1). Smp.: 102-105 °C. ^{1}H-NMR: (DMSO-D_6, 400 MHz), δ [ppm] = 9.70 (s, 1H), 7.91 (d, ^3J = 3.9 Hz, 1H), 7.55 (d, ^3J = 3.9 Hz, 1H), 7.26 (t, ^3J = 7.8 Hz, 1H), 7.18 (d, ^3J = 7.8 Hz, 1H), 7.11 (s, 1H), 6.81 (d, ^3J = 8.0 Hz, 1H), 3.21 (q, ^3J = 8.2 Hz, 4H), 2.97 (t, ^3J = 7.2 Hz, 2H), 2.34 (t, ^3J = 7.2 Hz, 2H), 1.85 (quin, ^3J = 7.2 Hz, 2H), 1.49-1.38 (m, 4H), 1.29-1.18 (m, 4H), 0.87 (t, ^3J = 7.3 Hz, 6H). ^{13}C-NMR: (DMSO-D_6, 400 MHz), δ [ppm] = 193.0 (1C), 170.9 (1C), 158.0 (1C), 151.3 (1C), 142.3 (1C), 134.3 (1C), 133.9 (1C), 130.4 (1C), 124.9 (1C), 116.8 (1C), 116.3 (1C), 112.6 (1C), 46.8 (1C), 46.7 (1C), 37.4 (1C), 31.2 (1C), 30.8 (1C), 29.6 (1C), 20.4 (1C), 19.7 (1C), 19.5 (1C), 13.8 (1C), 13.7 (1C). IR: ν [cm^{-1}] = 3175 (w), 2953 (m), 2866 (m), 1702 (w), 1655 (s), 1605 (vs), 1591 (vs), 1444 (vs), 1369 (m), 1312 (m), 1264 (m), 1227 (s), 1197 (m), 1085 (m), 1056 (m), 1000 (w), 929 (m), 866 (s), 783 (m), 733 (s), 687 (m), 592 (w), 535 (w), 459 (w). MS (ESI+): m/z (%) = 402 (100, $[M+H]^+$), 424 (20, $[M+Na]^+$). HRMS (ESI+): calculated for $C_{23}H_{32}NO_3S$: 402.2097; found: 402.2095.

N,N-Dibutyl-5-(5-(3-hydroxyphenyl)thiophene-2-yl)pentaneamide (8b)

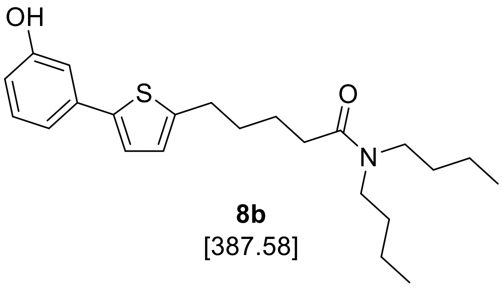

8b
[387.58]

The title compound was synthesised as described in the **general procedure 1A** with **2b** (200 mg, 0.72 mmol, 1.0 eq.), dibutylamine (0.12 mL, 0.72 mmol, 1.0 eq.), NEt_3 (0.30 mL, 2.16 mmol, 3.0 eq.), HOBt (146 mg, 1.08 mmol, 1.5 eq.) and EDC•HCl (208 mg, 1.08 mmol, 1.5 eq.) in DCM (50 mL). The crude product was purified *via* column chromatography (cyclohexane:EtOAc 3:1). 202 mg (0.56 mmol, 78%) of the desired product **8b** as pale-yellow solid were obtained. R_f = 0.23 (cyclohexane:EtOAc 3:1). Melting point: 80 °C. ^{1}H-NMR: (DMSO-D$_6$, 400 MHz), δ [ppm] = 9.51 (s, 1H), 7.21 (d, ^3J = 3.6 Hz, 1H), 7.17 (t, ^3J = 7.9 Hz, 1H), 7.00 (ddd, ^3J = 7.7 Hz, ^4J = 1.6 Hz, ^4J = 0.9 Hz, 1H), 6.95 (t, ^4J = 2.0 Hz, 1H), 6.81 (d, ^3J = 3.6 Hz, 1H), 6.67 (ddd, ^3J = 8.1 Hz, ^4J = 2.4 Hz, ^4J = 0.8 Hz, 1H), 3.20 (t, ^3J = 7.4 Hz, 4H), 2.79 (t, ^3J = 7.2 Hz, 2H), 2.29 (t, ^3J = 7.1 Hz, 2H), 1.68-1.53 (m, 4H), 1.50-1.38 (m, 4H), 1.30-1.17 (m, 4H), 0.88 (t, ^3J = 7.3 Hz, 3H), 0.86 (t, ^3J = 7.4 Hz, 3H). ^{13}C-NMR: (DMSO-D$_6$, 400 MHz), δ [ppm] = 171.2 (1C), 157.8 (1C), 144.6 (1C), 140.9 (1C), 135.2 (1C), 130.1 (1C), 125.6 (1C), 123.1 (1C), 115.8 (1C), 114.3 (1C), 111.7 (1C), 46.8 (1C), 44.6 (1C), 31.8 (1C), 30.9 (1C), 30.7 (1C), 29.6 (1C), 29.4 (1C), 24.5 (1C), 19.7 (1C), 19.5 (1C), 13.8 (1C), 13.7 (1C). IR: v [cm^{-1}] = 3187 (w), 2953 (m), 2928 (m), 2866 (m), 2836 (m), 1619 (vs), 1592 (vs), 1546 (w), 1513 (w), 1473 (m), 1448 (vs), 1414 (m), 1372 (m), 1306 (m), 1288 (m), 1252 (m), 1228 (s), 1213 (m), 1199 (m), 1162 (m), 1148 (m), 1115 (m), 1082 (w), 1074 (m), 987 (m), 976 (m), 863 (m), 856 (m), 824 (w), 813 (m), 787 (m), 732 (m), 709 (m), 684 (s), 655 (m), 626 (w), 596 (m), 546 (w), 494 (w), 443 (m), 407 (w). MS (ESI+): m/z (%) = 388 (100, [M+H]$^+$), 410 (22, [M+Na]$^+$). HRMS (ESI+): calculated for $C_{23}H_{34}NO_2S$: 388.2305; found: 388.2305.

5-(5-(3-Hydroxyphenyl)thiophene-2-yl)-5-oxo-*N,N*-dipentylpentaneamide (9a)

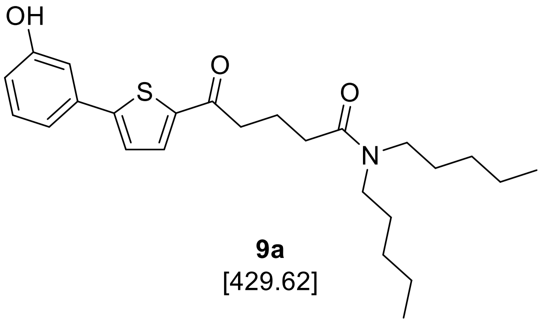

9a
[429.62]

The title compound was synthesised as described in the **general procedure 1A** with **2a** (300 mg, 1.03 mmol, 1.0 eq.), dipentylamine (0.2 mL, 1.03 mmol, 1.0 eq.), NEt_3 (0.43 mL, 3.09 mmol, 3.0 eq.), HOBt (209 mg, 1.55 mmol, 1.5 eq.) and EDC•HCl

(297 mg, 1.55 mmol, 1.5 eq.) in DCM (50 mL). The crude product was purified *via* column chromatography (cyclohexane:EtOAc 1:1). 143 mg (0.33 mmol, 32%) of the desired product **9a** as pale-yellow solid were obtained. R_f = 0.31 (cyclohexane:EtOAc 1:1). Melting point: 92-94 °C. ^{1}H-NMR: (DMSO-D$_6$, 400 MHz), δ [ppm] = 9.69 (s, 1H), 7.90 (d, ^3J = 3.9 Hz, 1H), 7.54 (d, ^3J = 3.9 Hz, 1H), 7.26 (t, ^3J = 7.9 Hz, 1H), 7.18 (d, ^3J = 7.8 Hz, 1H), 7.10 (s, 1H), 6.82 (d, ^3J = 8.0 Hz, 1H), 3.22-2.95 (m, 6H), 2.39-2.32 (m, 2H), 1.88-1.83 (m, 2H), 1.70-1.39 (m, 3H), 1.33-1.14 (m, 6H), 1.08-0.98 (m, 1H), 0.87-0.76 (m, 8H). ^{13}C-NMR: (DMSO-D$_6$, 100 MHz), δ [ppm] = 193.0 (1C), 171.4 (1C), 158.0 (1C), 151.4 (1C), 142.3 (1C), 134.3 (1C), 134.0 (1C), 130.4 (1C), 124.9 (1C), 116.8 (1C), 116.3 (1C), 112.6 (1C), 52.8, 50.2, 47.3, 47.0, 45.3, 44.9, 37.4, 33.8, 32.7, 31.5, 31.3, 31.2, 28.7, 28.7, 28.5, 28.4, 28.3, 28.1, 27.0, 26.6, 26.5, 26.3, 22.0, 21.9, 20.5, 20.4, 16.8, 16.5, 13.9, 13.9, 11.3, 11.2. In the aliphatic region multiple signal sets are visible. Therefore it is not possible to assign the signals to a single carbon atom.IR: ν [cm^{-1}] = 3248 (m), 3184 (m), 2957 (m), 2853 (m), 1689 (m), 1650 (vs), 1618 (vs), 1592 (vs), 1446 (vs), 1376 (m), 1306 (m), 1227 (s), 1087 (m), 1058 (m), 936 (m), 866 (s), 786 (vs), 751 (s), 728 (s), 592 (w), 535 (m), 464 (m). MS (ESI+): m/z (%) = 430 (100, [M+H]$^+$), 452 (30, [M+Na]$^+$).HRMS (ESI+): calculated for $C_{25}H_{36}NO_3S$: 430.2410; found: 430.2411.

5-(5-(3-Hydroxyphenyl)thiophene-2-yl)-*N*,*N*-dipentylpentaneamide (9b)

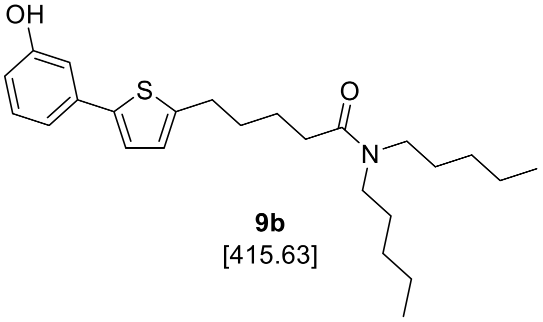

9b
[415.63]

The title compound was synthesised as described in the **general procedure 1A** with **2b** (200 mg, 0.72 mmol, 1.0 eq.), dipentylamine (0.15 mL, 0.72 mmol, 1.0 eq.), NEt_3 (0.30 mL, 2.16 mmol, 3.0 eq.), HOBt (146 mg, 1.08 mmol, 1.5 eq.) and EDC•HCl (208 mg, 1.08 mmol, 1.5 eq.) in DCM (50 mL). The crude product was purified *via* column chromatography (cyclohexane:EtOAc 3:1) and recrystallised with EtOH. 61 mg (0.15 mmol, 21%) of the desired product **9b** as yellow solid were obtained. R_f = 0.27 (cyclohexan:EtOAc 3:1). Melting point: 99 °C. ^{1}H-NMR: (DMSO-D$_6$, 400 MHz), δ [ppm]

= 9.49 (s, 1H), 7.21 (d, ^3J = 3.6 Hz, 1H), 7.17 (t, ^3J = 7.9 Hz, 1H), 7.00 (d, ^3J = 8.3 Hz, 1H), 6.95 (t, ^4J = 2.1 Hz, 1H), 6.81 (d, ^3J = 3.6 Hz, 1H), 6.67 (ddd, ^3J = 8.1 Hz, ^4J = 2.4 Hz, ^4J = 0.9 Hz, 1H), 3.22-3.00 (m, 4H), 2.79 (t, ^3J = 7.1 Hz, 2H), 2.23-2.27 (m, 2H), 1.67-1.55 (m, 4H), 1.51-1.36 (m, 4H), 1.34-1.13 (m, 4H), 1.10-0.99 (m, 2H), 0.88-0.81 (m, 6H), 0.77-0.75 (m, 2H). ^{13}C-NMR: (DMSO-D$_6$, 400 MHz), δ [ppm] = 171.6/171.1 (1C), 157.7 (1C), 144.5 (1C), 140.8 (1C), 135.1 (1C), 130.0 (1C), 125.5 (1C), 123.0 (1C), 115.8 (1C), 114.3 (1C), 111.7 (1C), 52.8, 50.1, 47.3, 47.0, 45.2, 44.8, 33.7, 32.6, 32.0, 31.8, 31.8, 30.6, 29.3, 28.6, 28.6, 28.4, 28.3, 28.1, 27.0, 26.6, 26.4, 26.3, 24.5, 21.9, 21.8, 16.5, 13.8, 13.8, 11.3, 11.1. In the aliphatic region multiple signal sets are visible. Therefore it is not possible to assign the signals to a single carbon atom.IR: ν [cm^{-1}] = 73254 (w), 2947 (w), 2926 (w), 2866 (w), 1616 (s), 1579 (m), 1483 (m), 1461 (m), 1432 (w), 1413 (w), 1375 (w), 1349 (w), 1307 (m), 1259 (w), 1217 (m), 1194 (w), 1160 (w), 1149 (w), 1107 (w), 1075 (w), 1050 (w), 995 (w), 875 (m), 842 (m), 817 (w), 805 (m), 777 (vs), 728 (m), 692 (m), 649 (w), 614 (w), 547 (w), 533 (w), 511 (w), 497 (w), 475 (w), 447 (m), 406 (w). MS (ESI+): m/z (%) = 416 (85, [M+H]$^+$), 438 (15, [M+Na]$^+$). HRMS (ESI+): calculated for $C_{25}H_{38}NO_2S$: 416.2618; found: 416.2616.

N-(Benzyloxy)-5-(5-(3-hydroxyphenyl)thiophene-2-yl)pentaneamide (47b)

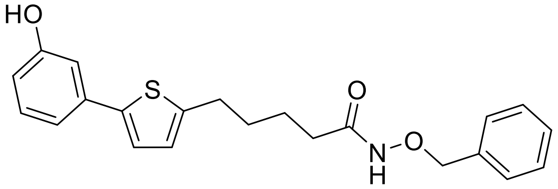

47b
[381.49]

As described in the **general procedure 1B** with **2b** (100 mg, 0.36 mmol, 1.0 eq.), oxalylchloride (0.04 mL, 0.54 mmol, 1.5 eq.), DMF (kat. amount) in DCM (1.0 mL) and O-benzyllhydroxylamine (0.08 mL, 0.54 mmol, 1.5 eq.), NEt_3 (0.08 mL, 0.54 mmol, 1.5 eq.) in DCM (1.0 mL). The crude product was purified *via* column chromatography (cyclohexane:EtOAc 4:1 → 1:1). 98.0 mg (0.26 mmol, 72%) of the desired product **47b** as colourless solid were obtained. R_f = 0.29 (cyclohexane:EtOAc 1:1). Melting point: 117 °C. ^{1}H-NMR: (DMSO-D$_6$, 400 MHz), δ [ppm] = 10.93 (s, 1H), 9.50 (s, 1H), 7.37-7.34 (m, 5H), 7.22 (d, ^3J = 3.5 Hz, 1H), 7.17 (t, ^3J = 7.9 Hz, 1H), 7.01 (d, ^3J = 7.7 Hz, 1H), 6.96 (t, ^4J = 1.6 Hz, 1H), 6.81 (d, ^3J = 3.3 Hz, 1H), 6.67 (d, ^3J = 8.2 Hz, 1H), 4.78 (s, 2H), 2.77 (t, ^3J = 5.9 Hz, 2H), 1.99 (brs, 2H), 1.58 (brs, 4H).

^{13}C-NMR: (DMSO-D_6, 400 MHz), δ [ppm] = 169.1 (1C), 157.7 (1C), 144.3 (1C), 140.9 (1C), 135.1 (1C), 130.0 (2C), 128.7 (1C), 128.2 (2C), 128.1 (1C), 125.6 (1C), 123.0 (1C), 115.8 (1C), 114.4 (1C), 114.3 (1C), 111.7 (1C), 59.7 (1C), 31.9 (1C), 34.4 (1C), 29.0 (1C), 24.3 (1C). IR: ν [cm^{-1}] = 3248 (m), 3064 (m), 2982 (w), 2948 (m), 2931 (m), 2836 (m), 1736 (m), 1662 (m), 1635 (m), 1594 (vs), 1546 (w), 1475 (m), 1459 (m), 1442 (s), 1427 (m), 1372 (m), 1310 (w), 1292 (m), 1238 (m), 1221 (s), 1196 (m), 1164 (m), 1084 (w), 1052 (m), 1026 (m), 982 (m), 915 (m), 854 (m), 803 (m), 779 (m), 744 (s), 698 (s), 685 (m), 624 (w), 605 (w), 518 (w), 495 (m), 467 (m), 440 (m), 407 (w). MS (ESI+): m/z (%) = 399 (20, $[M+NH_4]^+$), 404 (10, $[M+Na]^+$). HRMS (ESI+): calculated for $C_{22}H_{24}NO_3S$: 382.1471; found: 382.1486 and calculated for $C_{22}H_{23}NNaO_3S$: 404.1291; found: 404.1305.

N-Hydroxy-5-(5-(3-hydroxyphenyl)thiophene-2-yl)pentaneamide (48b)

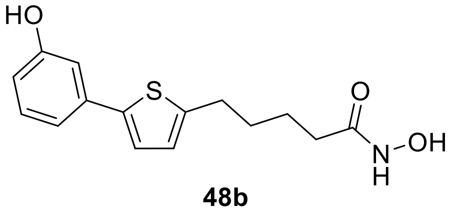

48b
[291.37]

The title compound was synthesised as described in the **general procedure 5** with **47b** (90.0 mg, 0.24 mmol, 1.0 eq.) and Pd/C 10 wt.% (2.6 mg, 0.24 mmol, 0.1 eq.) in MeOH (10 mL). The crude product was purified *via* HPLC (MeCN:H_2O). 18.0 mg (0.06 mmol, 25%) of the desired product **48b** as colourless solid were obtained. HPLC: >99% (46.8% MeCN). Melting point: 176 °C. ^{1}H-NMR: (DMSO-D_6, 400 MHz), δ [ppm] = 10.33 (s, 1H), 9.49 (s, 1H), 8.64 (s, 1H), 7.22 (d, ^3J = 3.5 Hz, 1H), 7.17 (t, ^3J = 7.9 Hz, 1H), 7.01 (d, ^3J = 7.7 Hz, 1H), 6.96 (t, ^4J = 1.6 Hz, 1H), 6.81 (d, ^3J = 3.4 Hz, 1H), 6.67 (d, ^3J = 8.1 Hz, 1H), 2.78 (t, ^3J = 7.1 Hz, 2H), 1.99 (t, ^3J = 6.8 Hz, 2H), 1.64-1.55 (m, 4H). ^{13}C-NMR: (DMSO-D_6, 400 MHz), δ [ppm] = 168.9 (1C), 157.7 (1C), 144.3 (1C), 140.8 (1C), 135.1 (1C), 130.0 (1C), 125.6 (1C), 123.0 (1C), 115.8 (1C), 114.3 (1C), 111.7 (1C), 31.9 (1C), 30.6 (1C), 29.1 (1C), 24.5 (1C). IR: ν [cm^{-1}] = 3340 (m), 3206 (w), 3046 (w), 2995 (w), 2914 (m), 2855 (m), 2800 (m), 2725 (w), 2672 (w), 2608 (w), 1656 (vs), 1587 (s), 1496 (m), 1473 (s), 1459 (s), 1415 (m), 1377 (w), 1316 (w), 1286 (w), 1250 (w), 1220 (s), 1193 (m), 1163 (m), 1143 (w), 1083 (w), 1055 (w), 1024 (w), 987 (w), 970 (m), 876 (w), 868 (w), 854 (m), 808 (m), 775 (s), 724 (w), 685 (m), 622

(w), 592 (w), 494 (m), 446 (s). MS (ESI+): m/z (%) = 292 (60, $[M+H]^+$), 309 (10, $[M+NH_4]^+$). HRMS (ESI+): calculated for $C_{15}H_{18}NO_3S$: 292.1002; found: 292.1001.

N-Ethyl-5-(5-(3-hydroxyphenyl)thiophene-2-yl)-5-oxopentaneamide (10a)

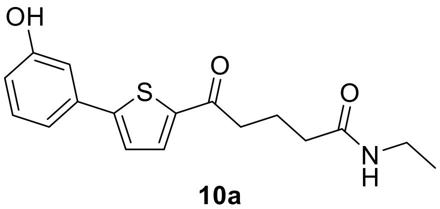

10a
[317.40]

The title compound was synthesised as described in the **general procedure 1A** with **2a** (200 mg, 0.69 mmol, 1.0 eq.), ethylamine (0.04 mL, 0.69 mmol, 1.0 eq.), NEt_3 (0.30 mL, 2.07 mmol, 3.0 eq.), HOBt (140 mg, 1.04 mmol, 1.5 eq.) and EDC•HCl (197 mg, 1.04 mmol, 1.5 eq.) in DCM (50 mL). The crude product was purified *via* column chromatography (cyclohexane:EtOAc 1:2). 76 mg (0.24 mmol, 35%) of the desired product **10a** as yellow solid were obtained. R_f = 0.17 (cyclohexane:EtOAc 1:2). Melting point: 190 °C. ^{1}H-NMR: (DMSO-D_6, 400 MHz), δ [ppm] = 9.70 (s, 1H), 7.90 (d, ^3J = 4.0 Hz, 1H), 7.76 (t, ^4J = 4.3 Hz, 1H), 7.55 (d, ^3J = 4.0 Hz, 1H), 7.26 (t, ^3J = 7.9 Hz, 1H), 7.20-7.17 (m, 1H), 7.11 (brs, 1H), 6.83-6.80 (m, 1H), 3.09-3.02 (m, 2H), 2.94 (t, ^3J = 7.2 Hz, 2H), 2.13 (t, ^3J = 7.4 Hz, 2H), 1.88-1.81 (m, 4H), 1.00 (t, ^3J = 7.2 Hz, 3H). ^{13}C-NMR: (DMSO-D_6, 400 MHz), δ [ppm] = 192.7 (1C), 171.2 (1C), 157.9 (1C), 151.3 (1C), 142.2 (1C), 134.2 (1C), 133.9 (1C), 130.4 (1C), 124.8 (1C), 116.8 (1C), 116.3 (1C), 112.6 (1C), 37.4 (1C), 34.4 (1C), 33.2 (1C), 20.4 (1C), 14.7 (1C). IR: v [cm^{-1}] = 3365 (m), 3177 (w), 2959 (w), 2940 (w), 1653 (m), 1636 (vs), 1610 (w), 1577 (m), 1551 (m), 1534 (m), 1446 (s), 1417 (w), 1375 (w), 1320 (m), 1303 (m), 1285 (m), 1241 (w), 1217 (m), 1201 (m), 1180 (m), 1152 (w), 1106 (w), 1075 (m), 1052 (w), 1035 (w), 994 (w), 922 (m), 870 (w), 845 (m), 811 (m), 776 (s), 753 (m), 721 (m), 687 (m), 657 (m), 589 (m), 536 (w), 468 (m), 438 (w), 401 (m). MS (ESI+): m/z (%) = 318 (100, $[M+H]^+$), 340 (50, $[M+Na]^+$). HRMS (ESI+): calculated for $C_{17}H_{20}NO_3S$: 318.1158; found: 318.1173.

N-Ethyl-5-(5-(3-hydroxyphenyl)thiophene-2-yl)pentaneamide (10b)

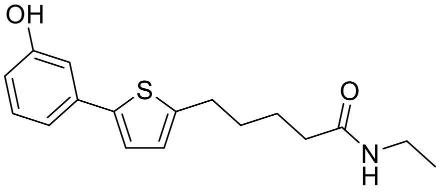

10b
[303.42]

The title compound was synthesised as described in the **general procedure 1A** with **2b** (100 mg, 0.36 mmol, 1.0 eq.), etylamine (0.10 mL, 0.36 mmol, 1.0 eq.), NEt_3 (0.20 mL, 1.08 mmol, 3.0 eq.), HOBt (37.0 mg, 0.54 mmol, 1.5 eq.) and EDC•HCl (104 mg, 0.54 mmol, 1.5 eq.) in DCM (25 mL). The crude product was purified *via* column chromatography (cyclohexane:EtOAc 1:1). 43 mg (0.14 mmol, 39%) of the desired product **10b** as yellow solid were obtained. R_f = 0.14 (cyclohexane:EtOAc 1:1). Melting point: 115 °C. ^{1}H-NMR: (DMSO-D_6, 400 MHz), δ [ppm] = 9.50 (s, 1H), 7.74 (brs, 1H), 7.22 (d, ^3J = 3.5 Hz, 1H), 7.17 (t, ^3J = 7.9 Hz, 1H), 7.01 (dd, ^3J = 7.6 Hz, ^4J = 1.0 Hz, 1H), 6.95 (d, ^4J = 1.5 Hz, 1H), 6.81 (d, ^3J = 3.5 Hz, 1H), 6.67 (d, ^3J = 8.1 Hz, 1H), 3.05 (quin, ^3J = 6.8 Hz, 2H), 2.78 (t, ^3J = 6.9 Hz, 2H), 2.08 (t, ^3J = 6.8 Hz, 2H), 1.64-1.53 (m, 4H), 1.00 (t, ^3J = 7.2 Hz, 3H). ^{13}C-NMR: (DMSO-D_6, 400 MHz), δ [ppm] = 171.7 (1C), 157.8 (1C), 144.5 (1C), 140.9 (1C), 135.2 (1C), 130.1 (1C), 125.7 (1C), 123.1 (1C), 115.9 (1C), 114.3 (1C), 111.7 (1C), 35.1 (1C), 33.2 (1C), 30.7 (1C), 29.2 (1C), 24.7 (1C), 14.9 (1C). IR: ν [cm^{-1}] = 3371 (w), 3279 (w), 2928 (m), 2881 (w), 2855 (w), 1612 (m), 1590 (s), 1541 (s), 1507 (m), 1461 (m), 1445 (s), 1421 (m), 1378 (m), 1320 (m), 1278 (m), 1266 (m), 1245 (w), 1227 (m), 1214 (m), 1161 (m), 1150 (m), 1076 (w), 1064 (w), 1046 (w), 1017 (w), 995 (w), 945 (w), 856 (m), 841 (m), 802 (m), 772 (vs), 741 (w), 727 (w), 682 (m), 623 (m), 599 (m), 572 (m), 530 (m), 496 (m), 464 (w), 443 (w), 417 (m). MS (ESI+): m/z (%) = 304 (100, $[M+H]^+$), 321 (31, $[M+NH_4]^+$, 326 (30, $[M+Na]^+$). HRMS (ESI+): calculated for $C_{17}H_{22}NO_2S$: 304.1366; found: 304.1372 and calculated for $C_{17}H_{21}NNaO_2S$: 326.1185; found: 326.1193.

N-Butyl-5-(5-(3-hydroxyphenyl)thiophene-2-yl)-5-oxopentaneamide (11a)

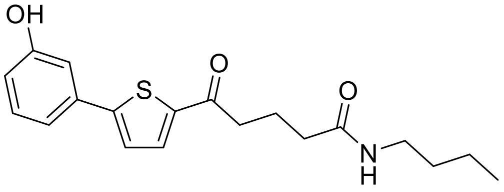

11a
[345.46]

The title compound was synthesised as described in the **general procedure 1A** with **2a** (200 mg, 0.70 mmol, 1.0 eq.), butylamine (0.04 mL, 0.70 mmol, 1.0 eq.), NEt_3 (0.30 mL, 2.10 mmol, 3.0 eq.), HOBt (140 mg, 1.04 mmol, 1.5 eq.) and EDC•HCl (199 mg, 1.04 mmol, 1.5 eq.) in DCM (50 mL). The crude product was purified *via* column chromatography (cyclohexane:EtOAc 1:1). 112 mg (0.32 mmol, 46%) of the desired product **11a** as pale-yellow solid were obtained. R_f = 0.15 (cyclohexane:EtOAc 1:1). Melting point: 134 °C. ^{1}H-NMR: (DMSO-D_6, 400 MHz), δ [ppm] = 9.71 (s, 1H), 7.89 (d, ^3J = 4.0 Hz, 1H), 7.74 (t, ^4J = 5.3 Hz, 1H), 7.55 (d, ^3J = 4.0 Hz, 1H), 7.26 (t, ^3J = 7.9 Hz, 1H), 7.18 (d, ^3J = 7.9 Hz, 1H), 7.11 (brs, 1H), 6.83-6.80 (m, 1H), 3.06-3.01 (m, 2H), 2.93 (t, ^3J = 7.2 Hz, 2H), 2.14 (t, ^3J = 7.4 Hz, 2H), 1.88-1.81 (m, 2H), 1.40-1.33 (m, 2H), 1.31-1.21 (m, 2H), 0.86 (t, ^3J = 7.3 Hz, 3H). ^{13}C-NMR: (DMSO-D_6, 400 MHz), δ [ppm] = 192.7 (1C), 171.3 (1C), 158.0 (1C), 151.3 (1C), 142.2 (1C), 134.2 (1C), 133.9 (1C), 130.4 (1C), 124.8 (1C), 116.8 (1C), 116.3 (1C), 112.6 (1C), 38.0 (1C), 37.4 (1C), 34.4 (1C), 31.2 (1C), 20.5 (1C), 19.5 (1C), 13.6 (1C). IR: ν [cm^{-1}] = 3441 (w), 3143 (w), 3099 (w), 3062 (w), 2953 (m), 2929 (m), 2900 (w), 2872 (w), 1654 (vs), 1591 (m), 1520 (m), 1441 (s), 1417 (m), 1370 (w), 1337 (w), 1316 (w), 1265 (m), 1221 (m), 1194 (m), 1161 (m), 1079 (w), 1054 (m), 1028 (w), 994 (w), 930 (w), 873 (m), 844 (m), 816 (m), 779 (m), 744 (s), 686 (m), 635 (w), 601 (w), 581 (w), 533 (w), 501 (w), 473 (m), 433 (m), 404 (w). MS (ESI+): m/z (%) = 346 (100, $[M+H]^+$). HRMS (ESI+): calculated for $C_{19}H_{23}NO_3S$: 346.1471; found: 346.1485.

N-Butyl-5-(5-(3-hydroxyphenyl)thiophene-2-yl)pentaneamide (11b)

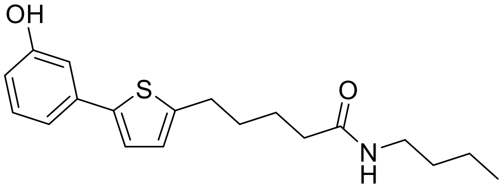

11b
[331.47]

The title compound was synthesised as described in the **general procedure 1A** with **2b** (200 mg, 0.72 mmol, 1.0 eq.), butylamine (0.04 mL, 0.72 mmol, 1.0 eq.), NEt_3 (0.30 mL, 2.16 mmol, 3.0 eq.), HOBt (146 mg, 1.08 mmol, 1.5 eq.) and EDC•HCl (208 mg, 1.08 mmol, 1.5 eq.) in DCM (50 mL). The crude product was purified *via* column chromatography (cyclohexane:EtOAc 1:1). 218 mg (0.66 mmol, 92%) of the desired product **11b** as yellow solid were obtained. R_f = 0.43 (cyclohexane:EtOAc 1:1). Melting point:100 °C. ^{1}H-NMR: (DMSO-D_6, 400 MHz), δ [ppm] = 9.49 (s, 1H), 7.72 (t, ^3J = 4.8 Hz, 1H), 7.21 (d, ^3J = 3.6 Hz, 1H), 7.17 (t, ^3J = 7.9 Hz, 1H), 7.00 (ddd, ^3J = 7.7 Hz, ^4J = 1.7 Hz, ^4J = 0.9 Hz, 1H), 6.95 (t, ^4J = 2.1 Hz, 1H), 6.81 (d, ^3J = 3.6 Hz, 1H), 6.67 (ddd, ^3J = 8.1 Hz, ^4J = 2.4 Hz, ^4J = 0.9 Hz, 1H), 3.02 (q, ^3J = 6.8 Hz, 2H), 2.78 (t, ^3J = 6.9 Hz, 2H), 2.09 (t, ^3J = 6.9 Hz, 2H), 1.62-1.54 (m, 4H), 1.40-1.31 (m, 2H), 1.29-1.22 (m, 2H), 0.86 (t, ^3J = 7.2 Hz, 3H). ^{13}C-NMR: (DMSO-D_6, 400 MHz), δ [ppm] = 171.7 (1C), 157.8 (1C), 144.5 (1C), 140.9 (1C), 135.2 (1C), 130.1 (1C), 125.6 (1C), 123.1 (1C), 115.8 (1C), 114.3 (1C), 111.7 (1C), 38.0 (1C), 35.0 (1C), 31.3 (1C), 30.7 (1C), 29.2 (1C), 24.8 (1C), 19.6 (1C), 13.7 (1C). IR: ν [cm^{-1}] = 3362 (w), 3227 (w), 2938 (m), 2899 (w), 2857 (w), 2836 (w), 1618 (m), 1594 (vs), 1542 (s), 1508 (w), 1474 (w), 1460 (w), 1441 (s), 1416 (w), 1377 (w), 1361 (w), 1325 (w), 1291 (m), 1264 (w), 1224 (s), 1197 (m), 1163 (m), 1140 (w), 1115 (w), 1086 (w), 987 (w), 975 (w), 855 (m), 814 (s), 808 (m), 778 (s), 732 (m), 683 (m), 624 (m), 598 (m), 581 (m), 533 (w), 493 (m), 481 (w), 459 (w), 442 (m). MS (ESI+): m/z (%) = 332 (100, $[M+H]^+$), 349 (20, $[M+NH_4]^+$, 354 (30, $[M+Na]^+$). HRMS (ESI+): calculated for $C_{19}H_{26}NO_2S$: 332.1679; found: 332.1678.

_N_-Benzyl-5-(5-(3-hydroxyphenyl)thiophene-2-yl)-_N_-methyl-5-oxopentaneamide (12a)

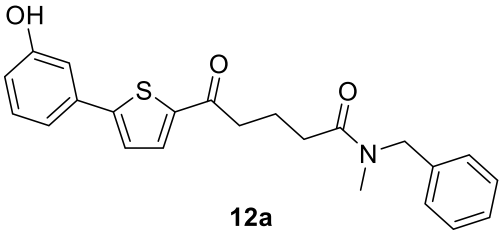

12a
[393.50]

The title compound was synthesised as described in the **general procedure 1A** with **2a** (200 mg, 0.69 mmol, 1.0 eq.), benzylmethylamine (0.04 mL, 0.69 mmol, 1.0 eq.), NEt_3 (0.30 mL, 2.07 mmol, 3.0 eq.), HOBt (140 mg, 1.04 mmol, 1.5 eq.) and EDC•HCl (197 mg, 1.04 mmol, 1.5 eq.) in DCM (50 mL). The crude product was purified *via* column chromatography (cyclohexane:EtOAc 1:1). 211 mg (0.54 mmol, 78%) of the desired product **12a** as yellow solid were obtained. R_f = 0.2 (cyclohexane:EtOAc 1:1). Melting point:157 °C. ^{1}H-NMR: (DMSO-D$_6$, 400 MHz), δ [ppm] = 9.70 (s, 1H), 7.92-7.90 (m, 1H), 7.56-7.54 (m, 1H), 7.37-7.17 (m, 7H), 7.11 (brs, 1H), 6.82 (d, ^3J = 7.1 Hz, 1H), 4.57/4.52 (s, 2H), 3.03-2.95 (m, 2H), 2.90/2.81 (s, 3H), 2.47-2.42 (t, ^3J = 6.8 Hz, 2H), 1.94-1.85 (m, 4H). ^{13}C-NMR: (DMSO-D$_6$, 100 MHz), δ [ppm] = 199.9 (1C), 171.8 (1C), 157.9 (1C), 151.3 (1C), 142.2 (1C), 138.0/137.7 (1C), 134.3/133.9 (1C), 130.4 (1C), 128.7/128.5 (2C), 127.5/127.2 (2C), 127.0 (1C), 126.5 (1C), 124.9 (1C), 116.9 (1C), 116.3 (1C), 112.6 (1C), 52.3/49.8 (1C), 37.5 (1C), 34.6/33.4 (1C), 31.6/31.4 (1C), 20.1/20.0 (1C). IR: ν [cm^{-1}] = 3210 (w), 3087 (w), 3073 (w), 3055 (w), 3031 (w), 2972 (w), 2943 (w), 2903 (w), 1647 (s), 1608 (vs), 1579 (s), 1531 (w), 1494 (w), 1441 (s), 1412 (m), 1373 (w), 1352 (w), 1321 (m), 1289 (m), 1262 (m), 1240 (m), 1223 (m), 1200 (m), 1190 (m), 1180 (m), 1163 (m), 1152 (m), 1116 (m), 1080 (m), 1064 (m), 1029 (w), 995 (w), 947 (w), 886 (w), 872 (m), 844 (w), 817 (w), 804 (m), 770 (vs), 750 (m), 726 (s), 701 (m), 683 (m), 625 (m), 593 (w), 561 (w), 538 (w), 459 (w), 438 (m), 422 (m), 415 (m). MS (ESI+): m/z (%) = 394 (100, [M+H]$^+$), 316 (27, [M+Na]$^+$). HRMS (ESI+): calculated for $C_{23}H_{24}NO_3S$: 394.1471; found: 394.1469.

N-Benzyl-5-(5-(3-hydroxyphenyl)thiophene-2-yl)-N-methylpentaneamide (12b)

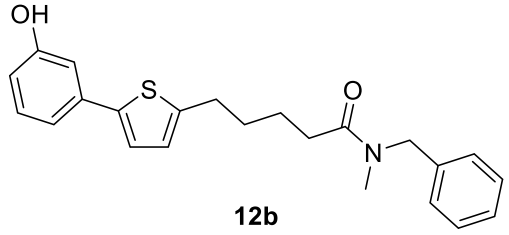

12b
[379.52]

The title compound was synthesised as described in the **general procedure 1A** with **2b** (200 mg, 0.72 mmol, 1.0 eq.), benzylmethylamine (0.06 mL, 0.72 mmol, 1.0 eq.), NEt_3 (0.30 mL, 2.16 mmol, 3.0 eq.), HOBt (146 mg, 1.08 mmol, 1.5 eq.) and EDC•HCl (208 mg, 1.08 mmol, 1.5 eq.) in DCM (50 mL). The crude product was purified *via* column chromatography (cyclohexane:EtOAc 1:1) and recrystallised from EtOAc. 116 mg (0.31 mmol, 43%) of the desired product **12b** as yellow solid were obtained. R_f = 0.40 (cyclohexane:EtOAc 1:1). Melting point: 117 °C. ^{1}H-NMR: (DMSO-D_6, 400 MHz), δ [ppm] = 9.50 (s, 1H), 7.39-7.15 (m, 7H), 7.02-6.99 (m, 1H), 6.96-6.95 (m, 1H), 6.83-6.77 (m, 1H), 6.67 (dd, ^3J = 7.8 Hz, ^4J = 2.2 Hz, 1H), 4.57/4.50 (s, 2H), 2.84-2.73 (m, 2H), 2.90/2.80 (s, 3H), 2.44-2.36 (m, 2H), 1.71-1.60 (m, 4H). ^{13}C-NMR: (DMSO-D_6, 100 MHz), δ [ppm] = 172.1 (1C), 157.8 (1C), 144.6 (1C), 140.9 (1C), 138.0/137.7 (1C), 135.2 (1C), 130.1 (1C), 128.7/128.5 (2C), 127.4/127.2 (2C), 126.9/126.5 (1C), 125.7 (1C), 123.1 (1C), 115.9 (1C), 114.5/114.3 (1C), 111.7 (1C), 52.4/49.9 (1C), 34.7/33.5 (1C), 33.1/31.7 (1C), 30.8/30.7 (1C), 29.4/29.2 (1C), 24.3/24.1 (1C). IR: ν [cm^{-1}] = 3179 (w), 3060 (w), 3029 (w), 2950 (w), 2927 (w), 2865 (w), 2836 (w), 1624 (vs), 1593 (s), 1546 (w), 1476 (m), 1445 (m), 1419 (m),1374 (w), 1354 (w), 1313 (w), 1291 (m), 1241 (m), 1229 (m), 1198 (w), 1185 (w), 1163 (w), 1114 (w), 1085 (w), 1071 (w), 1046 (w), 1027 (w), 988 (w), 978 (w), 914 (w), 861 (m), 813 (s), 785 (m), 747 (m), 738 (m), 712 (m), 696 (s), 684 (s), 645 (w), 625 (w), 597 (m), 561 (w), 539 (w), 494 (w), 463 (w), 443 (w), 410 (m). MS (ESI+): m/z (%) = 380 (100, $[M+H]^+$). HRMS (ESI+): calculated for $C_{23}H_{26}NO_2S$: 380.1679; found: 380.1677 and calculated for $C_{23}H_{25}NNaO_2S$: 402.1498; found: 402.1502.

Methyl N-(5-(5-(3-hydroxyphenyl)thiophene-2-yl)-5-oxopentanoyl)glycinate (13a)

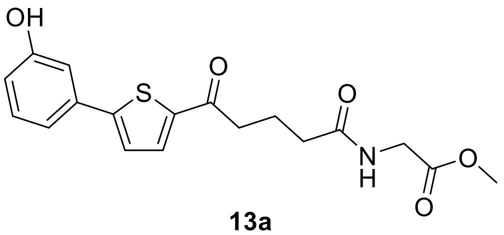

13a
[361.41]

The title compound was synthesised as described in the **general procedure 1A** with **2a** (200 mg, 0.69 mmol, 1.0 eq.), glycine methyl ester•HCl (87.0 mg, 0.69 mmol, 1.0 eq.), NEt_3 (0.30 mL, 2.10 mmol, 3.0 eq.), HOBt (140 mg, 1.04 mmol, 1.5 eq.) and EDC•HCl (199 mg, 1.04 mmol, 1.5 eq.) in DCM (50 mL). The crude product was purified *via* column chromatography (cyclohexane:EtOAc 1:2) and recrystallised with EtOAc. 197 mg (0.55 mmol, 80%) of the desired product **13a** as yellow solid were obtained. R_f = 0.22 (cyclohexane:EtOAc 1:3). Melting point:135 °C. ^{1}H-NMR: (DMSO-D_6, 400 MHz), δ [ppm] = 9.70 (s, 1H), 8.28 (t, ^3J = 5.7 Hz), 7.91 (d, ^3J = 4.0 Hz, 1H), 7.55 (d, ^3J = 4.0 Hz, 1H), 7.26 (t, ^3J = 7.9 Hz, 1H), 7.11 (dd, ^3J = 8.0 Hz, ^4J = 1.3 Hz, 1H), 7.11 (brs, 1H), 6.81 (ddd, ^3J = 8.1 Hz, ^4J = 2.3 Hz, ^4J = 1.1 Hz,1H), 3.83 (d, ^3J = 5.9 Hz, 2H), 3.63 (s, 3H), 2.79 (t, ^3J = 7.3 Hz, 2H), 2.23 (t, ^3J = 7.3 Hz, 2H), 1.86 (quin, ^3J = 7.1 Hz, 2H). ^{13}C-NMR: (DMSO-D_6, 100 MHz), δ [ppm] = 192.7 (1C), 172.4 (1C), 170.6 (1C), 158.0 (1C), 151.4 (1C), 142.2 (1C), 134.3 (1C), 133.9 (1C), 130.4 (1C), 124.9 (1C), 116.9 (1C), 116.3 (1C), 112.6 (1C), 51.7 (1C), 40.6 (1C), 37.2 (1C), 34.0 (1C), 20.3 (1C). IR: ν [cm^{-1}] = 3394 (m), 3296 (w), 3241 (w), 3226 (w), 2945 (w), 2903 (w), 1735 (m), 1681 (m), 1654 (w), 1629 (s), 1595 (m), 1529 (m), 1459 (s), 1435 (m), 1409 (w), 1400 (w), 1376 (m), 1323 (w), 1289 (w), 1213 (s), 1203 (vs), 1164 (m), 1112 (w), 1092 (m), 1062 (w), 1037 (w), 1004 (w), 994 (w), 967 (w), 945 (w), 863 (m), 810 (m), 790 (m), 756 (w), 712 (w), 683 (m), 662 (m), 645 (m), 608 (w), 584 (w), 560 (m), 545 (w), 534 (w), 507 (w), 494 (w), 462 (w), 455 (w), 436 (m), 410 (w). MS (ESI+): m/z (%) = 362 (100, $[M+H]^+$), 379 (14, $[M+NH_4]^+$, 384 (14, $[M+Na]^+$). HRMS (ESI+): calculated for $C_{18}H_{20}NO_5S$: 362.1057; found: 362.1055 and calculated for $C_{18}H_{19}NNaO_5S$: 384.0876; found: 384.0800.

5-(5-(3-Hydroxyphenyl)thiophene-2-yl)-5-oxopentanoyl)glycine (14a)

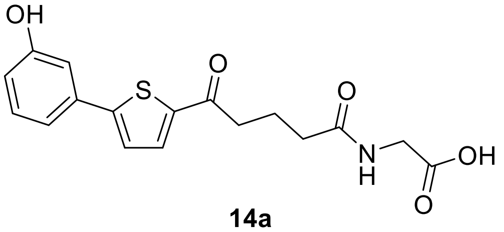

14a
[347.39]

The title compound was synthesised as described in the **general procedure 2A** with **13a** (120 mg, 0.33 mmol, 1.0 eq.) and KOH (55 mg, 0.99 mmol, 3.0 eq.) in MeOH (2 mL). The crude product was washed with EtOAc. 59 mg (0.19 mmol, 58%) of the desired product **14a** as yellow solid were obtained. Melting point: 206 °C. ^{1}H-NMR: (DMSO-D$_6$, 400 MHz), δ [ppm] = 12.49 (s, 1H), 9.70 (s, 1H), 8.16 (t, ^3J = 5.4 Hz), 7.90 (d, ^3J = 3.7 Hz, 1H), 7.54 (d, ^3J = 3.9 Hz, 1H), 7.26 (t, ^3J = 7.7 Hz, 1H), 7.18 (d, ^3J = 8.2 Hz, 1H), 7.11 (s, 1H), 6.81 (d, ^3J = 6.7 Hz, 1H), 3.74 (d, ^3J = 5.5 Hz, 2H), 2.98 (t, ^3J = 6.8 Hz, 2H), 2.22 (t, ^3J = 7.2 Hz, 2H), 1.90-1.83 (m, 2H). ^{13}C-NMR: (DMSO-D$_6$, 100 MHz), δ [ppm] = 192.8 (1C), 172.2 (1C), 171.5 (1C), 158.0 (1C), 151.4 (1C), 142.5/142.3 (1C), 134.3/133.9 (1C), 130.5 (1C), 124.8 (1C), 116.9 (1C), 116.3 (1C), 114.5 (1C), 112.6 (1C), 40.6 (1C), 37.2 (1C), 34.1 (1C), 20.4 (1C). IR: ν [cm^{-1}] = 3427 (w), 3258 (w), 2476 (w), 1712 (m), 1648 (s), 1606 (s), 1579 (vs), 1540 (m), 1532 (m), 1495 (w), 1444 (s), 1416 (m), 1406 (m), 1325 (m), 1299 (w), 1265 (m), 1245 (m), 1224 (m), 1210 (m), 1188 (m), 1109 (m), 1085 (w), 1061 (w), 1032 (w), 994 (w), 934 (m), 904 (m), 884 (m), 846 (m), 809 (m), 773 (vs), 755 (m), 685 (m), 668 (s), 643 (m), 585 (m), 551 (m), 537 (m), 502 (w), 479 (w), 433 (w). MS (ESI+): m/z (%) = 348 (100, [M+H]$^+$), 365 (5, [M+NH$_4$]$^+$, 370 (26, [M+Na]$^+$). HRMS (ESI+): calculated for $C_{17}H_{17}NO_5S$: 348.0900; found: 348.0900 and calculated for $C_{17}H_{17}NNaO_5S$: 370.0720; found: 370.0721.

Methyl *N*-(5-(5-(3-hydroxyphenyl)thiophen-2-yl)pentanoyl)glycinate (13b)

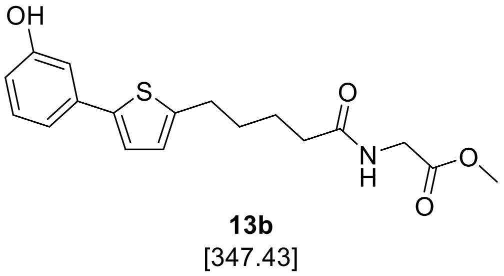

13b
[347.43]

The title compound was synthesised as described in the **general procedure 1A** with **2b** (200 mg, 0.72 mmol, 1.0 eq.), glycine methyl ester•HCl (91.0 mg, 0.72 mmol, 1.0 eq.), NEt_3 (0.30 mL, 2.16 mmol, 3.0 eq.), HOBt (146 mg, 1.08 mmol, 1.5 eq.) and EDC•HCl (208 mg, 1.08 mmol, 1.5 eq.) in DCM (50 mL). The crude product was purified *via* column chromatography (cyclohexane:EtOAc 1:2) and recrystallised from EtOAc. 190 mg (0.55 mmol, 76%) of the desired product **13b** as yellow solid were obtained. R_f = 0.29 (cyclohexane:EtOAc 1:2). Melting point: 131 °C. ^{1}H-NMR: (DMSO-D_6, 400 MHz), δ [ppm] = 9.50 (s, 1H), 8.24 (t, ^3J = 6.4 Hz), 7.22 (d, ^3J = 3.6 Hz, 1H), 7.17 (t, ^3J = 7.9 Hz, 1H), 7.01 (ddd, ^3J = 7.9 Hz, ^4J = 1.6 Hz, ^4J = 0.8 Hz, 1H), 6.95 (t, ^4J = 1.9 Hz, 1H), 6.82 (d, ^3J = 3.6 Hz, 1H), 6.67 (ddd, ^3J = 8.1 Hz, ^4J = 2.3 Hz, ^4J = 0.9 Hz,1H), 3.81 (d, ^3J = 5.9 Hz, 2H), 3.61 (s, 3H), 2.79 (t, ^3J = 7.1 Hz, 2H), 2.18 (t, ^3J = 6.9 Hz, 2H), 1.67-1.54 (m, 4H). ^{13}C-NMR: (DMSO-D_6, 100 MHz), δ [ppm] = 172.6 (1C), 170.5 (1C), 157.8 (1C), 144.5 (1C), 140.9 (1C), 135.2 (1C), 130.1 (1C), 125.7 (1C), 123.1 (1C), 115.8 (1C), 114.3 (1C), 111.7 (1C), 51.6 (1C), 40.5 (1C), 34.6 (1C), 30.5 (1C), 29.2 (1C), 24.6 (1C). IR: ν [cm^{-1}] = 3390 (w), 3211 (w), 3189 (w), 3170 (w), 2941 (w), 2910 (w), 2848 (w), 2175 (w), 2023 (w), 1743 (s), 1624 (m), 1595 (m), 1542 (m), 1508 (m), 1475 (w), 1458 (w), 1443 (m), 1433 (s), 1409 (w), 1395 (m), 1359 (m), 1322 (w), 1292 (w), 1259 (w), 1225 (s), 1210 (m), 1197 (m), 1162 (m), 1123 (m), 1084 (w), 1065 (w), 1048 (w), 1017 (w), 987 (w), 972 (m), 855 (m), 808 (m), 777 (m), 734 (m), 685 (m), 668 (w), 618 (m), 591 (m), 564 (w), 539 (s), 522 (vs), 493 (s), 484 (m), 457 (w), 442 (m), 423 (w), 411 (m). MS (ESI+): m/z (%) = 348 (100, $[M+H]^+$), 365 (35, $[M+NH_4]^+$, 370 (10, $[M+Na]^+$). HRMS (ESI+): calculated for $C_{18}H_{22}NO_4S$: 348.1264; found: 348.1260 and calculated for $C_{18}H_{21}NNaO_4S$: 370.1083; found: 370.1086.

N-(5-(5-(3-Hydroxyphenyl)thiophene-2-yl)pentanoyl)glycine (14b)

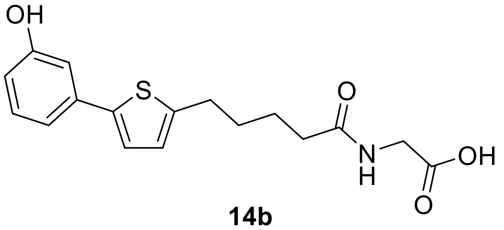

14b
[333.40]

The title compound was synthesised as described in the **general procedure 2A** with **13b** (120 mg, 0.33 mmol, 1.0 eq.) and KOH (55 mg, 0.99 mmol, 3.0 eq.) in MeOH

(2 mL). The crude product was recrystallised in EtOAc. 59 mg (0.18 mmol, 55%) of the desired product **14b** as beige solid were obtained. Melting point: 155 °C. ^{1}H-NMR: (DMSO-D$_6$, 400 MHz), δ [ppm] = 12.45 (s, 1H), 9.50 (s, 1H), 8.11 (t, ^{3}J = 5.9 Hz), 7.21 (d, ^{3}J = 3.6 Hz, 1H), 7.17 (t, ^{3}J = 7.9 Hz, 1H), 7.01 (ddd, ^{3}J = 8.1 Hz, ^{4}J = 1.0 Hz, ^{4}J = 1.0 Hz, 1H), 6.95 (t, ^{4}J = 2.1 Hz, 1H), 6.82 (d, ^{3}J = 3.6 Hz, 1H), 6.67 (dd, ^{3}J = 8.1 Hz, ^{4}J = 2.3 Hz, 1H), 3.72 (d, ^{3}J = 5.9 Hz, 2H), 2.79 (t, ^{3}J = 7.1 Hz, 2H), 2.17 (t, ^{3}J = 7.0 Hz, 2H), 1.68-1.54 (m, 4H). ^{13}C-NMR: (DMSO-D$_6$, 100 MHz), δ [ppm] = 172.3 (1C), 171.4 (1C), 157.7 (1C), 144.4 (1C), 140.8 (1C), 135.2 (1C), 130.1 (1C), 125.6 (1C), 123.0 (1C), 115.8 (1C), 114.3 (1C), 111.7 (1C), 40.5 (1C), 34.7 (1C), 30.5 (1C), 29.1 (1C), 24.6 (1C). IR: ν [cm^{-1}] = 3357 (w), 3318 (w), 2911 (w), 2856 (w), 1724 (s), 1645 (m), 1611 (m), 1579 (m), 1546 (m), 1500 (m), 1473 (m), 1459 (m), 1440 (m), 1427 (m), 1414 (m), 1348 (m), 1311 (m), 1236 (s), 1212 (vs), 1181 (m), 1164 (m), 1106 (w), 1069 (w), 1052 (w), 1039 (w), 1013 (w), 995 (w), 985 (w), 909 (w), 892 (w), 858 (m), 841 (s), 800 (w), 790 (w), 773 (vs), 731 (w), 683 (m), 655 (m), 627 (m), 603 (w), 584 (w), 568 (w), 547 (w), 533 (m), 495 (s), 477 (m), 458 (w), 441 (s), 423 (w), 404 (m). MS (ESI+): m/z (%) = 334 (100, [M+H]$^+$), 351 (50, [M+NH$_4$]$^+$, 356 (17, [M+Na]$^+$). HRMS (ESI+): calculated for $C_{17}H_{20}NO_4S$: 334.1108; found: 334.1111 and calculated for $C_{17}H_{19}NNaO_4S$: 356.0927; found: 356.0937.

Methyl *N*-(5-(5-(3-hydroxyphenyl)thiophene-2-yl)-5-oxopentanoyl)-L-alaninate (15a)

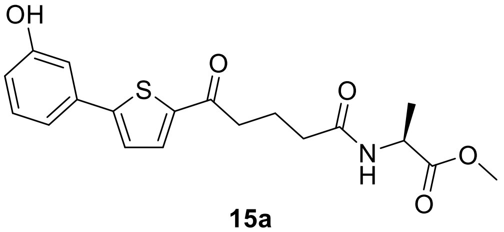

15a
[375.44]

The title compound was synthesised as described in the **general procedure 1A** with **2a** (200 mg, 0.69 mmol, 1.0 eq.), L-alanine methyl ester•HCl (96 mg, 0.69 mmol, 1.0 eq.), NEt$_3$ (0.30 mL, 2.10 mmol, 3.0 eq.), HOBt (140 mg, 1.04 mmol, 1.5 eq.) and EDC•HCl (199 mg, 1.04 mmol, 1.5 eq.) in DCM (50 mL). The crude product was purified *via* column chromatography (cyclohexane:EtOAc 1:2). 186 mg (0.50 mmol, 72%) of the desired product **15a** as pale solid were obtained. R$_f$ = 0.23 (cyclohexane:EtOAc 1:2). Melting point: 125 °C. ^{1}H-NMR: (DMSO-D$_6$, 400 MHz), δ

[ppm] = 9.70 (s, 1H), 8.26 (d, ^3J = 7.4 Hz), 7.90 (d, ^3J = 4.0 Hz, 1H), 7.56 (d, ^3J = 4.0 Hz, 1H), 7.26 (t, ^3J = 7.8 Hz, 1H), 7.19 (d, ^3J = 7.9 Hz, 1H), 7.11 (brs, 1H), 6.82 (dd, ^3J = 8.1 Hz, ^4J = 2.1 Hz, 1H), 4.29-4.22 (m, 1H), 3.62 (s, 3H), 2.96 (t, ^3J = 7.2 Hz, 2H), 2.20 (t, ^3J = 7.4 Hz, 2H), 1.89-1.82 (m, 2H), 1.26 (d, ^3J = 7.3 Hz, 3H). ^{13}C-NMR: (DMSO-D$_6$, 100 MHz), δ [ppm] = 192.7 (1C), 173.3 (1C), 171.7 (1C), 157.9 (1C), 151.3 (1C), 142.2 (1C), 134.2 (1C), 133.9 (1C), 130.4 (1C), 124.8 (1C), 116.8 (1C), 116.3 (1C), 112.6 (1C), 51.7 (1C), 47.5 (1C), 37.2 (1C), 33.9 (1C), 20.3 (1C), 16.84 (1C). IR: v [cm^{-1}] = 3409 (w), 3248 (w), 2953 (w), 2899 (w), 1739 (m), 1654 (vs), 1592 (m), 1515 (m), 1441 (s), 1416 (m), 1382 (w), 1336 (m), 1316 (w), 1300 (w), 1274 (w), 1265 (w), 1218 (s), 1195 (m), 1179 (m), 1160 (m), 1148 (m), 1076 (w), 1051 (m), 994 (w), 978 (w), 932 (w), 867 (w), 844 (w), 817 (w), 792 (w), 782 (m), 747 (m), 687 (m), 647 (w), 603 (w), 582 (w), 549 (w), 511 (w), 471 (m), 449 (w), 435 (w), 421 (w). MS (ESI+): m/z (%) = 376 (100, [M+H]$^+$), 393 (7, [M+NH$_4$]$^+$, 397 (13, [M+Na]$^+$). HRMS (ESI+): m/z calculated for $C_{19}H_{22}NO_5S$: 376.1213; found: 376.1214 and calculated for $C_{19}H_{21}NNaO_5S$: 398.1033; found: 398.1041.

N-(5-(5-(3-Hydroxyphenyl)thiophene-2-yl)-5-oxopentanoyl)-L-alanine (16a)

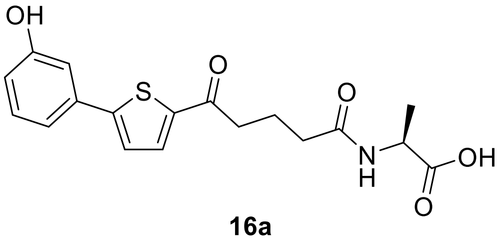

16a
[361.41]

The title compound was synthesised as described in the **general procedure 2A** with **15a** (120 mg, 0.32 mmol, 1.0 eq.) and KOH (54 mg, 0.96 mmol, 3.0 eq.) in MeOH (2 mL). The crude product was washed with EtOAc. 52 mg (0.14 mmol, 44%) of the desired product **16a** as yellow solid were obtained. Melting point:155 °C. ^{1}H-NMR: (DMSO-D$_6$, 400 MHz), δ [ppm] = 12.42 (s, 1H), 9.71 (s, 1H), 8.13 (d, ^3J = 7.1 Hz, 1H), 7.90 (d, ^3J = 3.7 Hz, 1H), 7.54 (d, ^3J = 3.7 Hz, 1H), 7.28-7.11 (m, 3H), 6.82 (d, ^3J = 7.4 Hz, 1H), 4.23-4.16 (m, 1H), 2.96 (brs, 2H), 2.20 (brs, 2H), 1.85 (brs, 2H), 1.25 (d, ^3J = 7.2 Hz). ^{13}C-NMR: (DMSO-D$_6$, 100 MHz), δ [ppm] = 192.8 (1C), 174.4 (1C), 171.6 (1C), 158.0 (1C), 151.4 (1C), 142.3 (1C), 134.3/133.9 (1C), 130.5 (1C), 124.9 (1C), 116.9 (1C), 116.3 (1C), 114.5 (1C), 112.6 (1C), 47.5 (1C), 37.2 (1C), 34.1 (1C),

20.4 (1C), 17.1 (1C). IR: ν [cm^{-1}] = 3287 (m), 3082 (w), 2943 (w), 1727 (m), 1630 (vs), 1595 (s), 1530 (m), 1455 (s), 1407 (s), 1373 (m), 1319 (m), 1288 (m), 1221 (s), 1163 (s), 1093 (w), 1056 (w), 1003 (w), 995 (w), 928 (w), 864 (w), 806 (m), 782 (m), 753 (w), 683 (m), 646 (w), 584 (w), 533 (w), 509 (w), 434 (w). MS (ESI+): m/z (%) = 362 (100, $[M+H]^+$), 379 (10, $[M+NH_4]^+$. HRMS (ESI-): calculated for $C_{18}H_{18}NO_5S$: 360.0911; found: 360.0907.

N-Methyl (5-(5-(3-hydroxyphenyl)thiophene-2-yl)pentanoyl)-L-alaninate (15b)

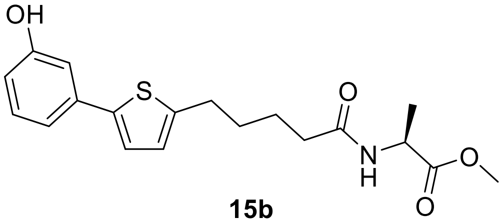

15b
[361.46]

The title compound was synthesised as described in the **general procedure 1A** with **2b** (200 mg, 0.72 mmol, 1.0 eq.), L-alanine methyl ester•HCl (100 mg, 0.72 mmol, 1.0 eq.), NEt_3 (0.30 mL, 2.16 mmol, 3.0 eq.), HOBt (146 mg, 1.08 mmol, 1.5 eq.) and EDC•HCl (208 mg, 1.08 mmol, 1.5 eq.) in DCM (25 mL). The crude product was purified *via* column chromatography (cyclohexane:EtOAc 1:1). 196 mg (0.54 mmol, 75%) of the desired product **15b** as yellow solid were obtained. R_f = 0.24 (cyclohexane:EtOAc 1:1). Melting point: 79 °C. ^{1}H-NMR: (DMSO-D$_6$, 400 MHz), δ [ppm] = 9.50 (s, 1H), 8.22 (d, ^3J = 6.6 Hz, 1H), 7.22 (d, ^3J = 3.6 Hz, 1H), 7.17 (t, ^3J = 7.9 Hz, 1H), 7.02-7.00 (m, 1H), 6.95 (t, ^4J = 1.9 Hz, 1H), 6.82 (d, ^3J = 3.6 Hz, 1H), 6.68-6.65 (m, 1H), 4.29-4.21 (m, 1H), 3.60 (s, 3H), 2.79 (t, ^3J = 7.0 Hz, 2H), 2.15 (t, ^3J = 7.0 Hz, 2H), 1.64-1.55 (m, 4H), 1.26 (d, ^3J = 7.3 Hz, 3H). ^{13}C-NMR: (DMSO-D$_6$, 100 MHz), δ [ppm] = 173.2 (1C), 171.9 (1C), 157.8 (1C), 144.4 (1C), 140.8 (1C), 135.2 (1C), 130.0 (1C), 125.6 (1C), 123.0 (1C), 115.8 (1C), 114.3 (1C), 111.7 (1C), 51.7 (1C), 47.4 (1C), 34.5 (1C), 30.5 (1C), 29.1 (1C), 24.5 (1C), 16.9 (1C). IR: ν [cm^{-1}] = 3335 (w), 3168 (w), 2943 (m), 2870 (w), 1746 (m), 1735 (m), 1655 (m), 1638 (m), 1595 (m), 1539 (s), 1476 (m), 1459 (m), 1445 (m), 1434 (m), 1418 (m), 1377 (m), 1345 (m), 1310 (m), 1287 (m), 1258 (m), 1238 (m), 1219 (s), 1209 (vs), 1162 (m), 1077 (m), 1059 (m), 1045 (m), 987 (m), 952 (m), 910 (m), 854 (m), 807 (s), 778 (s), 733 (m), 689 (s), 624 (m), 549 (m), 507 (m), 443 (m), 406 (m). MS (ESI+): m/z (%) = 362 (100, $[M+H]^+$), 379 (21,

[M+NH_4]$^+$, 384 (5, [M+Na]$^+$). HRMS (ESI+) calculated for $C_{19}H_{24}NO_4S$: 3362.1421; found: 362.1427 and calculated for $C_{19}H_{23}NNaO_4S$: 384.1240; found: 384.1248.

N-(5-(5-(3-Hydroxyphenyl)thiophene-2-yl)pentanoyl)-L-alanine (16b)

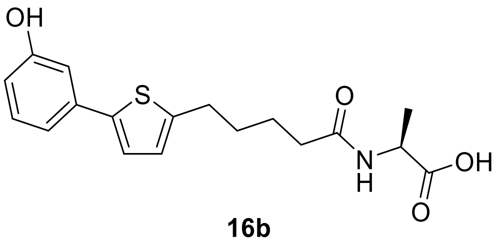

16b
[347.43]

The title compound was synthesised as described in **general procedure 2B** with **15b** (70 mg, 0.19 mmol, 1.0 eq.) und 2M NaOH (1.0 mL) in EtOH (2 mL). The crude product was purified *via* column chromatography (EtOAc + 0.1% formic acid). 39 mg (0.11 mmol, 58%) of the desired product **16b** as colourless solid were obtained. R_f = 0.24 (EtOAc + 0.1% formic acid). Melting point: 118 °C. ^{1}H-NMR: (DMSO-D_6, 500 MHz), δ [ppm] = 12.41 (s, 1H), 9.49 (s, 1H), 8.07 (d, ^3J = 7.3 Hz, 1H), 7.21 (d, ^3J = 3.6 Hz, 1H), 7.17 (t, ^3J = 7.9 Hz, 1H), 7.01 (ddd, ^3J = 7.7 Hz, ^4J = 1.7 Hz, ^4J = 1.0 Hz, 1H), 6.95 (t, ^4J = 2.2 Hz, 1H), 6.81 (d, ^3J = 3.6 Hz, 1H), 6.67 (ddd, ^3J = 8.1 Hz, ^4J = 2.4 Hz, ^4J = 0.9 Hz, 1H), 4.22-4.16 (m, 1H), 2.78 (t, ^3J = 7.2 Hz, 2H), 2.15 (t, ^3J = 7.1 Hz, 2H), 1.66-1.54 (m, 4H), 1.25 (d, ^3J = 7.3 Hz, 3H). ^{13}C-NMR: (DMSO-D_6, 125 MHz), δ [ppm] = 174.2 (1C), 171.8 (1C), 157.7 (1C), 144.5 (1C), 140.8 (1C), 135.2 (1C), 130.0 (1C), 125.5 (1C), 123.0 (1C), 115.8 (1C), 114.3 (1C), 111.7 (1C), 47.3 (1C), 34.6 (1C), 30.5 (1C), 29.1 (1C), 24.6 (1C), 17.2 (1C). IR: ν [cm^{-1}] = 3330 (m), 2937 (w), 2551 (w), 1722 (s), 1635 (s), 1614 (m), 1583 (m), 1552 (s), 1505 (m), 1482 (m), 1456 (m), 1415 (m), 1385 (w), 1288 (m), 1225 (s), 1185 (s), 1185 (m), 1166 (s), 1055 (w), 986 (m), 942 (m), 932 (m), 851 (m), 798 (s), 772 (vs), 740 (w), 679 (m), 612 (m), 572 (m), 534 (w), 470 (w), 612 (m), 572 (m), 534 (w), 470 (w), 440 (m), 418 (w), 409 (w). MS (ESI+): m/z (%) = 384 (100, [M+H]$^+$), 365 (100, [M+NH_4]$^+$. HRMS (ESI-) calculated for $C_{18}H_{20}NO_4S$: 346.1119; found: 346.1115.

Methyl *N*-(5-(5-(3-hydroxyphenyl)thiophene-2-yl)-5-oxopentanoyl)-L-phenylalaninat (17a)

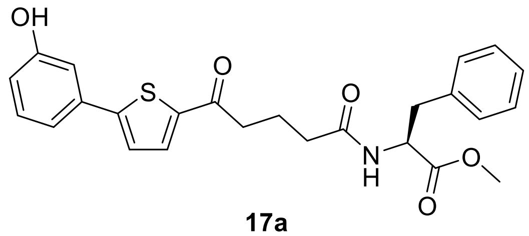

17a
[451.54]

The title compound was synthesised as described in the **general procedure 1A** with **2a** (200 mg, 0.69 mmol, 1.0 eq.), L-phenylalanine methyl ester•HCl (149 mg, 0.69 mmol, 1.0 eq.), NEt_3 (0.30 mL, 2.10 mmol, 3.0 eq.), HOBt (140 mg, 1.04 mmol, 1.5 eq.) and EDC•HCl (199 mg, 1.04 mmol, 1.5 eq.) in DCM (50 mL). The crude product was purified *via* column chromatography (cyclohexane:EtOAc 1:1). 189 mg (0.42 mmol, 61%) of the desired product **17a** as yellow solid were obtained. R_f = 0.23 (cyclohexane:EtOAc 1:1). Melting point: 142 °C. ^{1}H-NMR: (DMSO-D_6, 400 MHz), δ [ppm] = 9.70 (s, 1H), 8.31 (d, ^3J = 7.9 Hz, 1H), 7.82 (d, ^3J = 4.0 Hz, 1H), 7.55 (d, ^3J = 4.0 Hz, 1H), 7.28-7.15 (m, 7H), 7.11 (t, ^4J = 2.1 Hz, 1H), 6.82 (ddd, ^3J = 8.0 Hz, ^4J = 2.4 Hz, ^4J = 1.0 Hz, 1H), 4.52-4.47 (m, 1H), 3.60 (s, 3H), 3.06-2.87 (m, 2H), 2.84 (t, ^3J = 7.2 Hz, 2H), 2.19-2.09 (m, 2H), 1.78 (quin, ^3J = 6.6 Hz, 2H). ^{13}C-NMR: (DMSO-D_6, 100 MHz), δ [ppm] = 192.6 (1C), 172.2 (1C), 171.8 (1C), 157.9 (1C), 151.3 (1C), 142.1 (1C), 137.3 (1C), 134.2 (1C), 133.9 (1C), 130.4 (1C), 129.0 (2C), 128.2 (2C), 126.4 (1C), 124.8 (1C), 116.8 (1C), 116.3 (1C), 112.6 (1C), 53.4 (1C), 51.8 (1C), 37.1 (1C), 36.6 (1C), 34.0 (1C), 20.2 (1C). IR: ν [cm^{-1}] = 3314 (w), 2957 (w), 1750 (s), 1731 (m), 1651 (vs), 1632 (vs), 1595 (m), 1514 (s), 1454 (s), 1440 (s), 1409 (w), 1372 (w), 1314 (w), 1286 (w), 1273 (w), 1247 (w), 1223 (w), 1200 (s), 1173 (s), 1122 (w), 1087 (w), 1051 (w), 1004 (w), 994 (w), 985 (w), 928 (m), 866 (m), 841 (w), 807 (m), 785 (m), 751 (m), 725 (m), 699 (m), 685 (w), 637 (w), 586 (w), 565 (w), 549 (w), 531 (w), 523 (w), 509 (w), 491 (w), 465 (m), 434 (m), 403 (m). MS (ESI+): m/z (%) = 452 (21, $[M+H]^+$). HRMS (ESI+): calculated for $C_{25}H_{26}NO_5S$: 452.1526; found: 452.1532 and calculated for $C_{25}H_{25}NNaO_5S$: 474.1346; found: 474.1362.

N-(5-(5-(3-Hydroxyphenyl)thiophene-2-yl)-5-oxopentanoyl)-L-phenylalanine (18a)

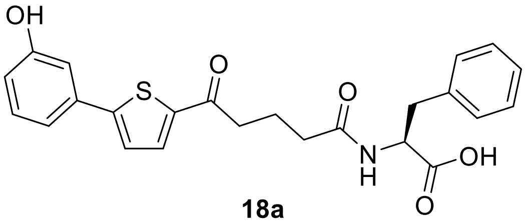

18a
[437.51]

The title compound was synthesised as described in the **general procedure 2A** with **17a** (120 mg, 0.27 mmol, 1.0 eq.) and KOH (15 mg, 0.27 mmol, 3.0 eq.) in MeOH (2 mL). 84 mg (0.19 mmol, 70%) of the desired product **18a** as yellow solid were obtained. Melting point: 144 °C. ^{1}H-NMR: (DMSO-D$_6$, 400 MHz), δ [ppm] = 12.64 (s, 1H), 9.70 (s, 1H), 8.16 (d, ^3J = 8.2 Hz), 7.81 (d, ^3J = 4.0 Hz, 1H), 7.55 (d, ^3J = 4.0 Hz, 1H), 7.28-7.14 (m, 7H), 7.11 (t, ^3J = 1.9 Hz, 1H), 6.82 (ddd, ^3J = 7.4 Hz, ^4J = 2.3 Hz, ^4J = 0.9 Hz, 1H), 4.48-4.42 (m, 1H), 3.07 (dd, ^3J = 4.8 Hz, ^2J = 13.9 Hz, 1H), 2.88-2.81 (m, 3H), 2.18-2.08 (m, 2H), 1.77 (quin, ^3J = 7.3 Hz, 2H). ^{13}C-NMR: (DMSO-D$_6$, 100 MHz), δ [ppm] = 192.6 (1C), 173.2 (1C), 171.7 (1C), 157.9 (1C), 151.3 (1C), 142.1 (1C), 137.8 (1C), 134.1 (1C), 133.8 (1C), 130.4 (1C), 129.0 (2C), 128.1 (2C), 126.3 (1C), 124.8 (1C), 116.8 (1C), 116.3 (1C), 112.6 (1C), 53.3 (1C), 37.1 (1C), 36.7 (1C), 34.1 (1C), 20.3 (1C). IR: ν [cm^{-1}] = 3341 (w), 3288 (w), 3262 (w), 3230 (w), 3185 (w), 3082 (w), 3065 (w), 3028 (w), 2921 (w), 1718 (m), 1630 (vs), 1594 (m), 1528 (m), 1497 (w), 1455 (s), 1438 (m), 1407 (m), 1380 (w), 1317 (m), 1286 (m), 1239 (m), 1222 (s), 1165 (m), 1114 (w), 1093 (w), 1056 (w), 1032 (w), 1003 (w), 995 (w), 925 (w), 865 (m), 807 (s), 783 (m), 749 (m), 698 (s), 685 (s), 640 (m), 587 (m), 537 (m), 509 (m), 488 (m), 475 (m), 463 (m), 434 (m), 409 (m), 403 (m). MS (ESI+): m/z (%) = 438 (100, $[M+H]^+$), 455 (10, $[M+NH_4]^+$. HRMS (ESI-): calculated for $C_{24}H_{24}NO_5S$: 438.1370; found: 438.1362 and calculated for $C_{24}H_{23}NNaO_5S$: 460.1189; found: 460.1187.

_N_-Methyl (5-(5-(3-hydroxyphenyl)thiophene-2-yl)pentanoyl)-L-phenylalaninate (17b)

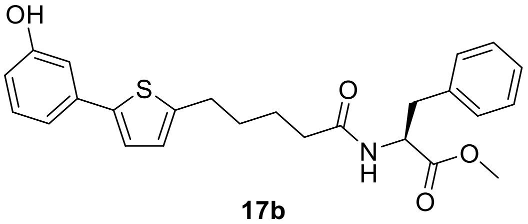

17b
[437.55]

The title compound was synthesised as described in **general procedure 1A** with **2b** (200 mg, 0.72 mmol, 1.0 eq.), L-phenylalanine methyl ester•HCl (155 mg, 0.72 mmol, 1.0 eq.), NEt_3 (0.30 mL, 2.16 mmol, 3.0 eq.), HOBt (146 mg, 1.08 mmol, 1.5 eq.) and EDC•HCl (208 mg, 1.08 mmol, 1.5 eq.) in DCM (50 mL). The crude product was purified *via* column chromatography (cyclohexane:EtOAc 2:1 → 1:1). 220 mg (0.50 mmol, 69%) of the desired product **17b** as yellow oil were obtained. DC: R_f = 0.36 (Cyclohexan:EtOAc 1:1). ^{1}H-NMR: (DMSO-D_6, 400 MHz), δ [ppm] = 9.50 (s, 1H), 8.26 (d, ^3J = 7.9 Hz, 1H), 7.27-7.15 (m, 7H), 7.01 (d, ^3J = 7.7 Hz, 1H), 6.96-6.95 (m, 1H), 6.79 (d, ^3J = 3.6 Hz, 1H), 6.67 (ddd, ^3J = 8.0 Hz, ^4J = 2.3 Hz, ^4J = 0.9 Hz,1H), 4.50-4.45 (m, 1H), 3.59 (s, 3H), 3.03 (dd, ^3J = 5.4 Hz, ^2J = 13.6 Hz, 1H), 2.87 (dd, ^3J = 9.6 Hz, ^2J = 13.7 Hz, 1H), 2.73 (t, ^3J = 6.0 Hz, 2H), 2.10 (t, ^3J = 6.5 Hz, 2H), 1.50-1.48 (m, 4H). The spectrum shows double signals (14%). ^{13}C-NMR: (DMSO-D_6, 125 MHz), δ [ppm] = 172.1 (1C), 172.0 (1C), 157.7 (1C), 144.4 (1C), 140.8 (1C), 137.3 (1C), 135.2 (1C), 130.0 (1C), 129.0 (2C), 128.1 (2C), 126.4 (1C), 125.5 (1C), 123.0 (1C), 115.8 (1C), 114.3 (1C), 111.7 (1C), 53.3 (1C), 51.7 (1C), 36.6 (1C), 34.6 (1C), 30.3 (1C), 29.1 (1C), 24.5 (1C). The spectrum shows also double signals. IR: ν [cm^{-1}] = 3288 (w), 3064 (w), 3029 (w), 2931 (w), 2858 (w), 1740 (m), 1646 (s), 1596 (m), 1580 (m), 1527 (m), 1497 (m), 1438 (s), 1362 (m), 1281 (m), 1215 (vs), 1178 (s), 1160 (s), 1129 (m), 1081 (w), 1060 (w), 1031 (w), 990 (m), 858 (m), 842 (m), 803 (m), 778 (s), 743 (m), 698 (s), 624 (m), 603 (w), 567 (w), 533 (w), 516 (w), 492 (m), 445 (w). MS (ESI+): m/z (%) = 438 (100, $[M+H]^+$), 455 (85, $[M+NH_4]^+$. HRMS (ESI+): calculated for $C_{25}H_{28}NO_4S$: 438.1734; found: 438.1739 and calculated for $C_{25}H_{27}NNaO_4S$: 460.1553; found: 460.1561.

_N_-(5-(5-(3-Hydroxyphenyl)thiophene-2-yl)pentanoyl)-L-phenylalanine (18b)

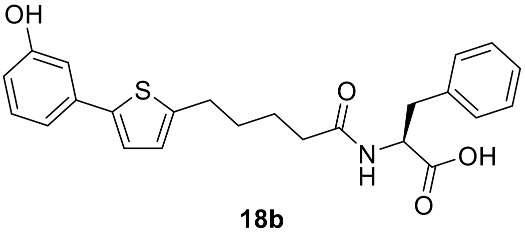

18b
[423.53]

The title compound was synthesised as described in **general procedure 2B** with **17b** (100 mg, 0.28 mmol, 1.0 eq.) and 2M NaOH (1.0 mL) in EtOH (2 mL). The crude product was purified *via* column chromatography (cyclohexane:EtOAc 1:1 + 0.1% formic acid). 49 mg (0.12 mmol, 43%) of the desired product **18b** as beige solid were obtained. R_f = 0.47 (EtOAc + 0.1% formic acid). Melting point: 72 °C. ^{1}H-NMR: (DMSO-D_6, 400 MHz), δ [ppm] = 12.64 (1H), 9.50 (s, 1H), 8.09 (d, ^3J = 8.3 Hz, 1H), 7.26-7.15 (m, 7H), 7.01 (d, ^3J = 8.9 Hz, 1H), 6.96 (s, 1H), 6.76 (d, ^3J = 3.3 Hz, 1H), 6.67 (d, ^3J = 8.2 Hz, 1H), 4.44-4.40 (m, 1H), 3.02 (dd, ^3J = 5.0 Hz, ^2J = 14.1 Hz), 2.83 (dd, ^3J = 10.0 Hz, ^2J = 14.0 Hz), 2.72 (t, ^3J = 6.9 Hz, 2H), 2.09 (t, ^3J = 6.5 Hz, 2H), 1.50-1.4 (m, 4H). ^{13}C-NMR: (DMSO-D_6, 100 MHz), δ [ppm] = 173.1 (1C), 171.9 (1C), 157.7 (1C), 144.4 (1C), 140.8 (1C), 137.7 (1C), 135.2 (1C), 130.0 (1C), 129.0 (2C), 128.1 (2C), 126.3 (1C), 125.5 (1C), 123.0 (1C), 115.8 (1C), 114.3 (1C), 111.7 (1C), 53.2 (1C), 36.7 (1C), 34.7 (1C), 30.3 (1C), 29.1 (1C), 24.5 (1C). IR: v [cm^{-1}] = 3315 (m), 3299 (m), 3272 (m), 3249 (m), 3183 (m), 3066 (m), 3030 (m), 2926 (m), 2859 (m), 2807 (m), 1717 (s), 1648 (m), 1636 (m), 1594 (s), 1579 (s), 1541 (s), 1523 (s), 1508 (s), 1497 (s), 1474 (m), 1446 (s), 1340 (m), 1314 (m), 1288 (m), 1218 (vs), 1178 (s), 1162 (s), 1082 (w), 1059 (w), 989 (w), 855 (m), 840 (m), 802 (m), 777 (s), 738 (m), 697 (s), 534 (w), 487 (w). MS (ESI+): m/z (%) = 424 (50, $[M+H]^+$), 446 (100, $[M+Na]^+$. HRMS (ESI+): calculated for $C_{24}H_{26}NO_4S$: 424.1577; found: 424.1571 and calculated for $C_{24}H_{25}NNaO_4S$: 446.1396; found: 446.1393.

Methyl *N*-(5-(5-(3-hydroxyphenyl)thiophene-2-yl)-5-oxopentanoyl)-L-prolinate (19a)

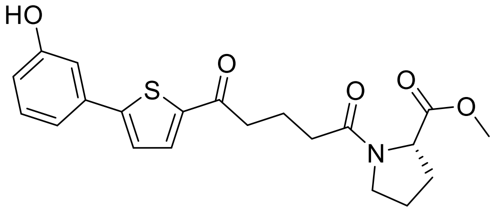

19a
[401.48]

The title compound was synthesised as described in the **general procedure 1A** with **2a** (200 mg, 0.69 mmol, 1.0 eq.), L-proline methyl ester•HCl (108 mg, 0.69 mmol, 1.0 eq.), NEt_3 (0.30 mL, 2.10 mmol, 3.0 eq.), HOBt (140 mg, 1.04 mmol, 1.5 eq.) and EDC•HCl (199 mg, 1.04 mmol, 1.5 eq.) in DCM (50 mL). The crude product was purified *via* column chromatography (Cyclohexan:EtOAc 1:4) and recrystallised of EtOAc. 217 mg (0.54 mmol, 78%) of the desired product **19a** as yellow solid were obtained. R_f = 0.31 (cyclohexane:EtOAc 1:1). Melting point:xx. ^{1}H-NMR: (DMSO-D_6, 400 MHz), δ [ppm] = 9.69 (s, 1H), 7.91 (d, ^3J = 4.0 Hz, 1H), 7.55 (d, ^3J = 4.0 Hz, 1H), 7.26 (t, ^3J = 7.9 Hz, 1H), 7.20-7.18 (m, 1H), 7.11 (t, ^4J = 1.9 Hz, 1H), 6.81 (ddd, ^3J = 8.1 Hz, ^4J = 2.3 Hz, ^4J = 0.9 Hz, 1H), 4.59/4.30 (dd, ^3J = 8.7 Hz, ^4J = 4.3 Hz, 1H), 3.67/3.62 (s, 3H), 3.54-3.38 (m, 2H), 3.00-2.93 (m, 2H), 2.41-2.31 (m, 2H), 2.21-2.01 (m, 1H), 1.93-1.80 (m, 5H). Double signals due to the cis-trans isomerism of proline.^{13}C-NMR: (DMSO-D_6, 100 MHz), δ [ppm] = 192.8 (1C), 172.6 (1C), 170.4 (1C), 157.9 (1C), 151.3 (1C), 142.2 (1C), 134.2 (1C), 133.8 (1C), 130.4 (1C), 124.8 (1C), 116.8 (1C), 116.3 (1C), 112.5 (1C), 58.5/58.1 (1C), 52.3/51.7 (1C), 46.5/45.9 (1C), 37.3/37.2 (1C), 32.5/32.3 (1C), 30.7/28.7 (1C), 24.4/22.1 (1C), 19.6/19.5 (1C). Double signals due to the cis-trans isomerism of proline. **IR:** ν [cm^{-1}] = 3160 (w), 2953 (w), 2896 (w), 1758 (m), 1742 (m), 1655 (m), 1615 (m), 1592 (m), 1536 (w), 1509 (w), 1444 (vs), 1412 (m), 1359 (w), 1338 (m), 1260 (w), 1223 (m), 1199 (m), 1180 (s), 1167 (s), 1100 (w), 1071 (w), 1056 (w), 1016 (w), 994 (w), 955 (w), 935 (w), 867 (m), 845 (m), 835 (w), 809 (w), 788 (w), 746 (m), 712 (w), 688 (m), 588 (w), 553 (w), 475 (w), 437 (w), 436 (w), 406 (w). MS (ESI+): m/z (%) = 402 (100, $[M+H]^+$). HRMS (ESI+) calculated for $C_{21}H_{24}NO_5S$: 402.1370; found: 402.1382.

N-(5-(5-(3-Hydroxyphenyl)thiophene-2-yl)-5-oxopentanoyl)-L-proline (20a)

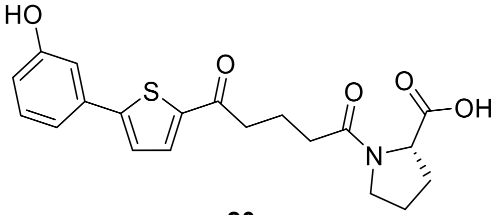

20a
[387.45]

The title compound was synthesised as described in the **general procedure 2A** with **19a** (140 mg, 0.35 mmol, 1.0 eq.) and KOH (59 mg, 1.05 mmol, 3.0 eq.) in MeOH (3 mL). 72 mg (0.19 mmol, 54%) of the desired product **20a** as yellow solid were obtained. Melting point: 184 °C. ^{1}H-NMR: (DMSO-D$_6$, 400 MHz), δ [ppm] = 12.42 (1H), 9.69 (s, 1H), 7.91 (d, ^{3}J = 3.5 Hz, 1H), 7.54 (d, ^{3}J = 3.5 Hz, 1H), 7.27 (t, ^{3}J = 7.2 Hz, 1H), 7.19 (d, ^{3}J = 8.5 Hz, 1H), 7.10 (s, 1H), 6.81 (d, ^{3}J = 7.0 Hz, 1H), 4.23 (d, ^{3}J = 7.2 Hz), 3.49 (brs, 2H), 3.01-2.94 (m, 2H), 2.36 (s, 2H), 2.19-2.07 (m, 2H), 1.91-1.84 (m, 4H). Double signals due to the cis/trans isomerism of proline. ^{13}C-NMR: (DMSO-D$_6$, 100 MHz), δ [ppm] = 192.9 (1C), 173.6 (1C), 170.3 (1C), 158.0 (1C), 151.3 (1C), 142.2 (1C), 134.3 (1C), 133.9 (1C), 130.4 (1C), 124.8 (1C), 116.9 (1C), 116.3 (1C), 112.6 (1C), 58.3 (1C), 46.5 (1C), 37.3 (1C), 32.6 (1C), 28.9 (1C), 24.4 (1C), 19.6 (1C). IR: ν [cm^{-1}] = 2956 (w), 2362 (w), 2162 (w), 2020 (w), 1977 (w), 1748 (w), 1733 (m), 1717 (m), 1700 (m), 1648 (m), 1670 (m), 1653 (m), 1624 (vs), 1594 (s), 1578 (s), 1559 (s), 1541 (m), 1533 (m), 1522 (m), 1507 (m), 1489 (m), 1473 (s), 1457 (s), 1437 (m), 1406 (w), 1387 (w), 1374 (w), 1363 (w), 1339 (m), 1319 (w), 1289 (w), 1268 (w), 1224 (m), 1194 (m), 1166 (m), 866 (m), 808 (m), 783 (m), 754 (w), 685 (m), 667 (w), 658 (w), 649 (w), 639 (w), 629 (w), 585 (w), 557 (w), 543 (w), 471 (w), 447 (w), 433 (m), 421 (m), 405 (m). MS (ESI+): m/z (%) = 388 (100, [M+H]$^+$). HRMS (ESI+): calculated for $C_{20}H_{22}NO_5S$: 388.1213; found: 388.1228.

Methyl N-(5-(5-(3-hydroxyphenyl)thiophene-2-yl)pentanoyl)-L-prolinate (19b)

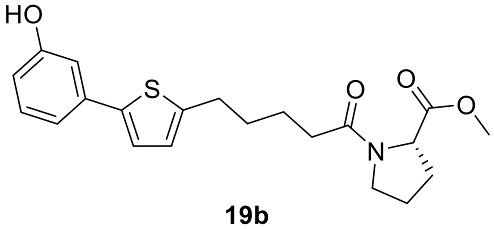

19b
[387.49]

The title compound was synthesised as described in the **general procedure 1A** with **2b** (200 mg, 0.72 mmol, 1.0 eq.), L-proline methyl ester•HCl (113 mg, 0.72 mmol, 1.0 eq.), NEt_3 (0.30 mL, 2.16 mmol, 3.0 eq.), HOBt (146 mg, 1.08 mmol, 1.5 eq.) and EDC•HCl (208 mg, 1.08 mmol, 1.5 eq.) in DCM (50 mL). The crude product was purified *via* column chromatography (cyclohexane:EtOAc 1:1). 146 mg (0.38 mmol, 53%) of the desired product **19b** as colourless solid were obtained. R_f = 0.20 (cyclohexane:EtOAc 1:1). Melting point: 130 °C. ^{1}H-NMR: (DMSO-D_6, 400 MHz), δ [ppm] = 9.50 (s, 1H), 7.22 (d, ^3J = 3.6 Hz, 1H), 7.17 (t, ^3J = 7.9 Hz, 1H), 7.01 (ddd, ^3J = 7.8 Hz, ^4J = 1.6 Hz, ^4J = 0.8 Hz, 1H), 6.95 (t, ^4J = 2.2 Hz, 1H), 6.82 (d, ^3J = 3.6 Hz, 1H), 6.67 (ddd, ^3J = 8.0 Hz, ^4J = 2.4 Hz, ^4J = 0.8 Hz,1H), 4.29-4.26 (m, 1H), 3.68/3.60 (s, 3H), 3.55-3.49 (m, 2H), 2.80 (t, ^3J = 7.4 Hz, 2H), 2.32 (t, ^3J = 6.9 Hz, 2H), 2.19-2.10 (m, 1H), 1.93-1.77 (m, 3H), 1.69-1.53 (m, 4H). Double signals due to the cis-trans isomerism of proline. ^{13}C-NMR: (DMSO-D_6, 100 MHz), δ [ppm] = 172.7 (1C), 170.7 (1C), 157.8 (1C), 144.5 (1C), 140.9 (1C), 135.2 (1C), 130.1 (1C), 125.7 (1C), 123.1 (1C), 115.9 (1C), 114.3 (1C), 111.7 (1C), 58.2 (1C), 51.7 (1C), 46.5 (1C), 33.9 (1C), 30.6 (1C), 29.3 (1C), 28.8 (1C), 24.4 (1C), 23.7 (1C). IR: ν [cm^{-1}] = 3139 (m), 2950 (m), 2871 (m), 1744 (s), 1625 (s), 1590 (s), 1474 (w), 1440 (vs), 1411 (m), 1368 (m), 1351 (w), 1335 (w), 1293 (w), 1278 (m), 1231 (s), 1214 (m), 1192 (s), 1172 (s), 1154 (s), 1098 (w), 1079 (w), 1057 (w), 1040 (w), 1010 (w), 994 (w), 986 (w), 948 (w), 914 (w), 875 (m), 856 (w), 845 (m), 808 (m), 780 (m), 756 (m), 730 (m), 693 (m), 680 (m), 631 (m), 605 (w), 554 (w), 502 (w), 446 (w). MS (ESI+): m/z (%) = 338 (100, $[M+H]^+$), 405 (50, $[M+NH_4]^+$. HRMS (ESI+): calculated for $C_{21}H_{26}NO_4S$: 388.1577; found: 388.1580 and calculated for $C_{21}H_{25}NNaO_4S$: 410.1396; found: 410.1413.

N-(5-(5-(3-Hydroxyphenyl)thiophene-2-yl)pentanoyl)-L-proline (20b)

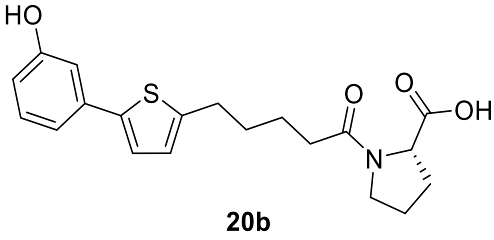

20b
[373.47]

The title compound was synthesised as described in the **general procedure 2B** with **19b** (100 mg, 0.33 mmol, 1.0 eq.) and 2M NaOH (1.0 mL) in EtOH (2 mL). 91 mg (0.24 mmol, 73%) of the desired product **20b** as colourless solid were obtained. Melting

point: 123 °C. ^{1}H-NMR: (DMSO-D$_6$, 400 MHz), δ [ppm] = 12.39 (s, 1H), 9.49 (s, 1H), 7.21 (d, ^{3}J = 3.6 Hz, 1H), 7.17 (t, ^{3}J = 7.9 Hz, 1H), 7.01 (ddd, ^{3}J = 7.7 Hz, ^{4}J = 1.7 Hz, ^{4}J = 0.9 Hz, 1H), 6.95 (t, ^{4}J = 2.0 Hz, 1H), 6.82 (d, ^{3}J = 3.6 Hz, 1H), 6.67 (dd, ^{3}J = 8.1 Hz, ^{4}J = 2.4 Hz, ^{4}J = 0.9 Hz, 1H), 4.48-4.46/4.22-4.20 (m, 1H), 3.54-3.35 (m, 2H), 2.81-2.75 (m, 2H), 2.32-2.02 (m, 3H), 1.92-1.79 (m, 3H), 1.70-1.54 (m, 4H). Double signals due to the cis-trans isomerism of proline.^{13}C-NMR: (DMSO-D$_6$, 100 MHz), δ [ppm] = 174.5/174.1 (1C), 171.2/171.0 (1C), 158.3 (1C), 145.1 (1C), 141.4 (1C), 135.7 (1C), 130.6 (1C), 126.1 (1C), 123.6 (1C), 116.4 (1C), 114.8 (1C), 112.3 (1C), 59.2/58.8 (1C), 47.0/46.4 (1C), 33.7/33.4 (1C), 31.4/31.1 (1C), 29.8 (1C), 29.4 (1C), 24.8/22.7 (1C), 24.3/24.2 (1C). Double signals due to the cis-trans isomerism of proline. IR: ν [cm^{-1}] = 3195 (m), 2947 (w), 2921 (m), 2886 (w), 1737 (m), 1635 (m), 1613 (m), 1589 (m), 1551 (w), 1509 (w), 1475 (w), 1448 (s), 1430 (m), 1407 (m), 1340 (m), 1316 (m), 1294 (m), 1223 (m), 1193 (m), 1161 (s), 1099 (w), 1086 (w), 986 (m), 909 (w), 866 (m), 854 (m), 829 (w), 799 (w), 778 (vs), 740 (m), 690 (m), 640 (m), 625 (m), 603 (w), 567 (w), 555 (w), 447 (m), 429 (w). MS (ESI+): m/z (%) = 374 (100, [M+H]$^+$), 396 (50, [M+Na]$^+$). HRMS (ESI+) calculated for $C_{20}H_{24}NO_4S$: 374.1421; found: 374.1410 and calculated for $20_7H_{23}NNaO_4S$: 396.1240; found: 396.1236.

4-*tert*-Butyl 1-methyl *N*-(5-(5-(3-hydroxyphenyl)thiophene-2-yl)-5-oxopentanoyl)-L-aspartate (21a)

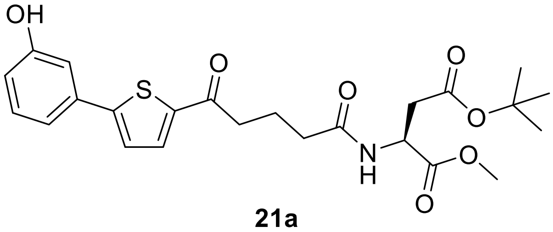

21a
[475.56]

The title compound was synthesised as described in the **general procedure 1A** with **2a** (200 mg, 0.69 mmol, 1.0 eq.), L-Asp(OtBu)-OMe•HCl (165 mg, 0.69 mmol, 1.0 eq.), NEt_3 (0.30 mL, 2.10 mmol, 3.0 eq.), HOBt (141 mg, 1.04 mmol, 1.5 eq.) and EDC•HCl (198 mg, 1.04 mmol, 1.5 eq.) in DCM (50 mL). The crude product was purified *via* column chromatography (cyclohexane:EtOAc 1:2) and recrystallized from EtOAc. 262 mg (0.55 mmol, 80%) of the desired product **21a** as yellow solid were obtained. R_f = 0.38 (cyclohexane:EtOAc 1:2). Melting point: 92 C. ^{1}H-NMR: (DMSO-D$_6$, 400 MHz), δ [ppm] = 9.70 (s, 1H), 8.32 (d, ^{3}J = 7.9 Hz, 1H), 7.89 (d, ^{3}J = 4.0 Hz, 1H),

7.55 (d, ^3J = 4.0 Hz, 1H), 7.26 (t, ^3J = 7.9 Hz, 1H), 7.18 (d, ^3J = 8.4 Hz, 1H), 7.11 (t, ^3J = 1.8 Hz, 1H), 6.81 (ddd, ^3J = 8.1 Hz, ^4J = 2.4 Hz, ^4J = 1.0 Hz), 4.64-4.59 (m, 1H), 3.62 (s, 3H), 2.95 (t, ^3J = 7.2 Hz, 2H), 2.69 (dd, ^3J = 6.1 Hz, ^2J = 16.0 Hz, 1H), 2.56 (dd, ^3J = 7.6 Hz, ^2J = 16.0 Hz, 1H), 2.20 (t, ^3J = 7.4 Hz, 2H), 1.85 (quin, ^3J = 7.3 Hz, 2H), 1.38 (s, 9H). ^{13}C-NMR: (DMSO-D$_6$, 100 MHz), δ [ppm] = 192.7 (1C), 171.8 (1C), 171.4 (1C), 169.1 (1C), 158.0 (1C), 151.4 (1C), 142.2 (1C), 134.3 (1C), 133.9 (1C), 130.4 (1C), 124.9 (1C), 116.9 (1C), 116.3 (1C), 112.6 (1C), 80.5 (1C), 52.1 (1C), 48.7 (1C), 37.1 (1C), 34.1 (1C), 27.6 (3C), 21.8 (1C), 20.4 (1C). IR: ν [cm^{-1}] = 3303 (w), 3292 (w), 3213 (w), 2977 (w), 1731 (s), 1652 (s), 1634 (s), 1596 (m), 1580 (m), 1527 (m), 1456 (m), 1438 (m), 1367 (m), 1287 (m), 1222 (m), 1208 (m), 1152 (vs), 1092 (w), 1050 (w), 1004 (w), 995 (w), 972 (w), 927 (w), 863 (w), 845 (w), 807 (m), 781 (m), 754 (w), 684 (m), 586 (w), 510 (w), 435 (m), 406 (m). MS (ESI+): m/z (%) = 476 (58, [M+H]$^+$). HRMS (ESI+):calculated for $C_{24}H_{30}NO_7S$: 476.1737; found: 476.1730.

***N*-(5-(5-(3-Hydroxyphenyl)thiophene-2-yl)-5-oxopentanoyl)-L-aspartic acid (22a)**

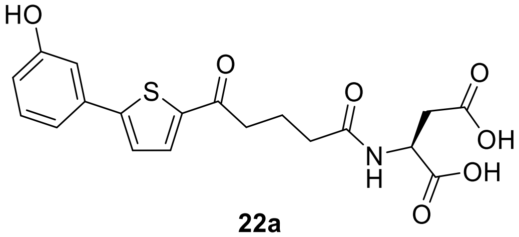

22a
[405.42]

The title compound was synthesised as described in the **general procedure 2A** with **21a** (166 mg, 0.41 mmol, 1.0 eq.) and KOH (69 mg, 1.23 mmol, 3.0 eq.) in MeOH (5 mL). 28 mg (0.07 mmol, 17%) of the desired product **22a** as yellow solid were obtained. Melting point: 154 C. ^{1}H-NMR: (DMSO-D$_6$, 400 MHz), δ [ppm] = 12.50 (s, 2H), 9.71 (s, 1H), 8.19 (d, ^3J = 7.9 Hz, 1H), 7.89 (d, ^3J = 3.9 Hz, 1H), 7.54 (d, ^3J = 3.7 Hz, 1H), 7.26 (t, ^3J = 7.8 Hz, 1H), 7.18 (d, ^3J = 7.9 Hz, 1H), 7.11 (s, 1H), 6.81 (d, ^3J = 7.9 Hz, 1H), 4.56-4.51 (m, 1H), 2.95 (t, ^3J = 7.3 Hz, 2H), 2.70 (dd, ^3J = 5.4 Hz, ^4J = 16.6 Hz, 1H), 2.60-2.53 (m, 1H), 2.19 (t, ^3J = 7.2 Hz, 2H), 1.84 (quin, ^3J = 6.9 Hz, 2H). ^{13}C-NMR: (DMSO-D$_6$, 400 MHz), δ [ppm] = 193.3 (1C), 173.2 (1C), 173.1 (1C), 172.24 (1C), 158.5 (1C), 151.9 (1C), 142.8 (1C), 134.8 (1C), 134.4 (1C), 130.9 (1C), 125.4 (1C), 117.4 (1C), 116.8 (1C), 113.1 (1C), 49.1 (1C), 37.7 (1C), 36.5 (1C), 34.7 (1C), 20.9 (1C). IR: ν [cm^{-1}] = 3204 (w), 2969 (w), 1717 (m), 1654 (m), 1631 (vs), 1595

(m), 1581 (m), 1541 (m), 1520 (m), 1456 (m), 1437 (m), 1407 (w), 1375 (w), 1283 (m), 1267 (m), 1238 (m), 1223 (m), 1209 (s), 1193 (m), 1166 (m), 1094 (w), 1058 (w), 1004 (w), 994 (w), 928 (w), 861 (w), 809 (m), 783 (m), 748 (w), 686 (m), 648 (w), 640 (w), 583 (w), 567 (w), 531 (w), 514 (w), 501 (w), 476 (w), 465 (w), 454 (w), 433 (w), 422 (m). MS (ESI-): m/z (%) = 404 (100, [M-H$^+$]). HRMS (ESI+) calculated for $C_{19}H_{20}NO_7S$: 406.0955; found: 406.0964.

4-*tert*-Butyl 1-methyl *N*-(5-(5-(3-hydroxyphenyl)thiophene-2-yl)pentanoyl)-L-aspartate (21b)

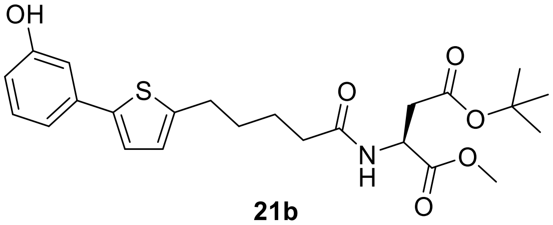

21b
[461.57]

The title compound was synthesised as described in the **general procedure 1A** with **2b** (200 mg, 0.72 mmol, 1.0 eq.), L-Asp(OtBu)-OMe•HCl (173 mg, 0.72 mmol, 1.0 eq.), NEt_3 (0.30 mL, 2.16 mmol, 3.0 eq.), HOBt (146 mg, 1.08 mmol, 1.5 eq.) and EDC•HCl (208 mg, 1.08 mmol, 1.5 eq.) in DCM (25 mL). The crude product was purified *via* column chromatography (cyclohexane:EtOAc 1:1). 205 mg (0.44 mmol, 61%) of the desired product **21b** as yellow oil were obtained. R_f = 0.35 (cyclohexane:EtOAc 1:1). ^{1}H-NMR: (DMSO-D$_6$, 500 MHz), δ [ppm] = 9.49 (s, 1H), 8.27 (d, ^3J = 8.0 Hz, 1H), 7.21 (d, ^3J = 3.6 Hz, 1H), 7.17 (t, ^3J = 7.9 Hz, 1H), 7.02-6.99 (m, 1H), 6.95 (t, ^4J = 2.1 Hz, 1H), 6.81 (d, ^3J = 3.6 Hz, 1H), 6.67 (ddd, ^3J = 8.1 Hz, ^4J = 2.4 Hz, ^4J = 0.9 Hz, 1H), 4.62-4.58 (m, 1H), 3.61 (s, 3H), 2.78 (t, ^3J = 7.0 Hz, 2H), 2.68 (dd, ^3J = 6.1 Hz, ^2J = 16.0 Hz, 1H), 2.55 (dd, ^3J = 7.6 Hz, ^2J = 16.0 Hz, 1H), 2.15 (t, ^3J = 7.0 Hz, 2H), 1.64-1.54 (m, 4H), 1.37 (s, 9H). ^{13}C-NMR: (DMSO-D$_6$, 125 MHz), δ [ppm] = 171.9 (1C), 171.3 (1C), 169.0 (1C), 157.7 (1C), 144.4 (1C), 140.8 (1C), 135.2 (1C), 130.0 (1C), 125.5 (1C), 123.0 (1C), 115.8 (1C), 114.3 (1C), 111.7 (1C), 80.4 (1C), 52.0 (1C), 48.6 (1C), 37.1 (1C), 34.6 (1C), 30.4 (1C), 29.1 (1C), 27.6 (3C), 24.6 (1C). IR: ν [cm^{-1}] = 3302 (w), 3068 (w), 2977 (w), 2933 (w), 2861 (w), 1728 (s), 1649 (m), 1596 (m), 1581 (m), 1524 (m), 1474 (m), 1438 (m), 1392 (m), 1366 (m), 1290 (m), 1218 (s), 1151 (vs), 1052 (w), 994 (w), 857 (w), 842 (m), 803 (m), 778 (m), 753 (w), 688 (m), 625 (w), 604 (w), 534 (w), 470 (w), 442 (w). MS (ESI+): m/z (%) = 462 (10, [M+H]$^+$), 484 (100,

[M+Na]⁺). HRMS (ESI+) calculated for $C_{24}H_{32}NO_6S$: 462.1945; found: 462.1940 and calculated for $C_{24}H_{31}NNaO_6S$: 484.1764; found: 484.1775.

***N*-(5-(5-(3-Hydroxyphenyl)thiophene-2-yl)pentanoyl)-L-aspartate (22b)**

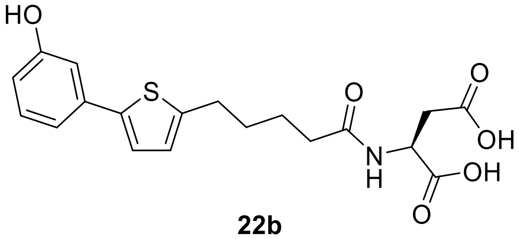

22b
[391.44]

The title compound was synthesised as described in the **general procedure 2B** with **21b** (242 mg, 0.52 mmol, 1.0 eq.) and 2M NaOH (4.0 mL) in EtOH (4 mL). The crude product was purified *via* column chromatography (EtOAc + 0.1% formic acid). 88 mg (0.22 mmol, 42%) of the desired product **22b** as colourless solid were obtained. R_f = 0.10 (EtOAc + 0.1% formic acid). Melting point: 153 °C. ^{1}H-NMR: (DMSO-D$_6$, 400 MHz), δ [ppm] = 12.50 (s, 2H), 9.49 (s, 1H), 8.12 (d, ^3J = 8.0 Hz, 1H), 7.21 (d, ^3J = 3.6 Hz, 1H), 7.17 (t, ^3J = 7.9 Hz, 1H), 7.01 (ddd, ^3J = 7.7 Hz, ^4J = 1.7 Hz, ^4J = 0.9 Hz, 1H), 6.95 (t, ^4J = 2.1 Hz, 1H), 6.81 (d, ^3J = 3.6 Hz, 1H), 6.67 (ddd, ^3J = 8.1 Hz, ^4J = 2.4 Hz, ^4J = 0.9 Hz, 1H), 4.54-4.50 (m, 1H), 2.78 (t, ^3J = 7.2 Hz, 2H), 2.68 (dd, ^3J = 5.8 Hz, ^2J = 16.4 Hz, 1H), 2.55 (dd, ^3J = 7.3 Hz, ^2J = 16.4 Hz, 1H), 2.15 (t, ^3J = 7.1 Hz, 2H), 1.65-1.53 (m, 4H). The spectrum shows double signals (5%). ^{13}C-NMR: (DMSO-D$_6$, 125 MHz), δ [ppm] = 172.5 (1C), 171.9 (1C), 171.6 (1C), 157.7 (1C), 144.3 (1C), 140.8 (1C), 135.2 (1C), 130.0 (1C), 125.5 (1C), 123.0 (1C), 115.8 (1C), 114.3 (1C), 111.7 (1C), 48.5 (1C), 36.1 (1C), 34.6 (1C), 30.4 (1C), 29.1 (1C), 24.6 (1C). IR: ν [cm^{-1}] = 3361 (w), 3068 (w), 3018 (w), 2947 (w), 2925 (m), 2864 (w), 2755 (w), 1713 (s), 1657 (s), 1593 (m), 1544 (s), 1475 (m), 1457 (m), 1447 (m), 1413 (m), 1337 (w), 1314 (w), 1254 (s), 1213 (vs), 1191 (m), 1168 (vs), 1087 /w), 985 (w), 971 (w), 900 (m), 872 (m), 851 (m), 796 (m), 772 (s), 730 (m), 684 (m), 634 (m), 614 (m), 583 (m), 556 (m), 537 (m), 485 (w), 476 (w), 441 (m), 423 (w). MS (ESI+): m/z (%) = 392 (10, [M+H]⁺), 414 (100, [M+Na]⁺). HRMS (ESI+) calculated for $C_{19}H_{22}NO_6S$: 392.1162; found: 392.1156 and calculated for: $C_{19}H_{21}NNaO_6S$: 414.0982; found: 414.0976.

N-Methyl (5-(5-(3-hydroxyphenyl)thiophene-2-yl)-5-oxopentanoyl)-L-histidinate (23a)

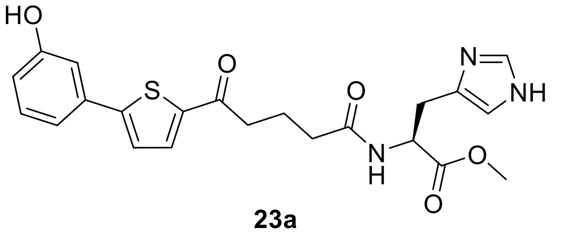

23a
[441.50]

The title compound was synthesised as described in the **general procedure 1A** with **2a** (200 mg, 0.69 mmol, 1.0 eq.), L-His-OMe•HCl (167 mg, 0.69 mmol, 1.0 eq.), NEt_3 (0.30 mL, 2.10 mmol, 3.0 eq.), HOBt (141 mg, 1.04 mmol, 1.5 eq.) and EDC•HCl (198 mg, 1.04 mmol, 1.5 eq.) in DCM (50 mL). The crude product was purified *via* column chromatography (DCM:MeOH 10:1). 298 mg (0.67 mmol, 97%) of the desired product **23a** as yellow solid were obtained. R_f = 0.23 (DCM:MeOH 10:1+1%formic acid). Melting point:153 C. ^{1}H-NMR: (DMSO-D_6, 400 MHz), δ [ppm] = 9.70 (s, 1H), 8.22 (d, ^3J = 7.6 Hz, 1H), 7.86 (d, ^3J = 3.9 Hz, 1H), 7.55 (d, ^3J = 3.9 Hz, 1H), 7.53 (s, 1H), 7.26 (t, ^3J = 7.9 Hz, 1H), 7.19 (d, ^3J = 7.7 Hz, 1H), 7.11 (s, 1H), 6.83-6.80 (m, 2H), 4.52-4.47 (m, 1H), 3.59 (s, 3H), 2.95-2.82 (m, 4H), 2.18 (t, ^3J = 7.0 Hz, 2H), 1.82 (quin, ^3J = 7.5 Hz, 2H). ^{13}C-NMR: (DMSO-D_6, 100 MHz), δ [ppm] = 192.6 (1C), 172.2 (1C), 171.8 (2C), 157.9 (2C), 151.3 (1C), 142.2 (1C), 134.8 (1C), 134.2 (1C), 133.9 (1C), 130.4 (1C), 124.8 (1C), 116.8 (1C), 116.3 (1C), 112.6 (1C), 52.3 (1C), 51.7 (2C), 37.1 (1C), 34.1 (1C), 20.3 (1C).IR: v [cm^{-1}] = 3294 (w), 3272 (w), 3238 (w), 3218 (w), 3197 (w), 2954 (w), 2175 (w), 1734 (m), 1654 (m), 1632 (vs), 1596 (m), 1578 (m), 1527 (m), 1457 (m), 1438 (ss), 1407 (m), 1375 (m), 1315 (m), 1289 (m), 1262 (m), 1240 (m), 1224 (s), 1207 (m), 1166 (m), 1094 (m), 1058 (w), 1004 (w), 995 (w), 929 (w), 867 (w), 847 (w), 808 (s), 782 (m), 751 (m), 722 (w), 711 (w), 685 (s), 661 (m), 626 (m), 586 (m), 555 (m), 535 (m), 511 (m), 501 (m), 472 (m), 461 (m), 451 (m), 434 (m), 415 (m), 404 (m). MS (ESI+): m/z (%) = 442 (100, $[M+H]^+$). HRMS (ESI+): calculated for $C_{22}H_{24}N_3O_5S$): 442.1431; found: 442.1449.

Methyl *N*-(5-(5-(3-hydroxyphenyl)thiophene-2-yl)pentanoyl)-L-histidinate (23b)

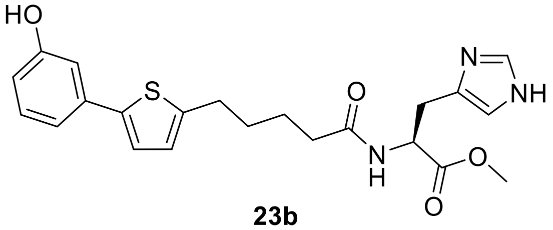

23b
[427.52]

The title compound was synthesised as described in the **general procedure 1A** with **2b** (200 mg, 0.72 mmol, 1.0 eq.), L-His-OMe•HCl (174 mg, 0.72 mmol, 1.0 eq.), NEt_3 (0.30 mL, 2.16 mmol, 3.0 eq.), HOBt (146 mg, 1.08 mmol, 1.5 eq.) and EDC•HCl (208 mg, 1.08 mmol, 1.5 eq.) in DCM (25 mL). The crude product was purified *via* column chromatography (DCM:MeOH 20:1). 98 mg (0.23 mmol, 32%) of the desired product **23b** as colourless solid were obtained. R_f = 0.10 (DCM:MeOH 20:1). Melting point: 73 °C. ^{1}H-NMR: (DMSO-D_6, 500 MHz), δ [ppm] = 9.53 (s, 1H), 8.18 (d, ^3J = 7.5 Hz, 0.5H), 7.91 (d, ^3J = 9.1 Hz, 0.5H), 7.62 (s, 1H), 7.44 (t, ^3J = 7.6 Hz, 0.5H), 7.34 (t, ^3J = 7.9 Hz, 0.5H), 7.22 (d, ^3J = 3.9 Hz, 1H), 7.17 (t, ^3J = 7.8 Hz, 1H), 7.01 (d, ^3J = 7.7 Hz, 1H), 7.00 (brs, 1H), 6.82 (s, 1H), 6.80 (d, ^3J = 4.3 Hz, 1H), 6.67 (dd, ^3J = 8.1 Hz, ^4J = 2.3 Hz, 1H), 4.51-4.47 (m, 1H), 3.58 (s, 3H), 2.93 (dd, ^3J = 5.9 Hz, ^2J = 14.3 Hz, 1H), 2.84 (dd, ^3J = 8.4 Hz, ^2J = 14.7 Hz, 1H), 2.76 (t, ^3J = 6.7 Hz, 2H), 2.13 (t, ^3J = 6.6 Hz, 2H), 1.59-1.49 (m, 4H). ^{13}C-NMR: (DMSO-D_6, 125 MHz), δ [ppm] = 172.1 (1C), 172.0 (1C), 157.8 (1C), 144.4 (1C), 142.8/140.8 (1C), 135.1 (1C), 134.7 (1C), 130.0 (1C), 127.7/125.5 (1C), 123.0 (1C), 126.4/124.0 (1C), 118.9 (1C), 115.8 (1C), 114.3 (1C), 111.7 (1C), 52.2 (1C), 51.7 (1C), 34.6 (1C), 30.3 (1C), 29.1 (1C), 28.7 (1C), 24.5 (1C).IR: ν [cm^{-1}] = 3140 (m), 3054 (m), 2924 (m), 2854 (m), 1735 (m), 1646 (m), 1593 (m), 1579 (m), 1541 (m), 1507 (m), 1436 (s), 1395 (m), 1379 (m), 1284 (m), 1215 (s), 1178 (s), 1124 (m), 1099 (m), 992 (w), 992 (w), 856 (w), 841 (m), 805 (m), 778 (s), 743 (s), 689 (m), 656 (w), 626 (m), 598 (m), 568 (m), 513 (w), 484 (m), 462 (w), 443 (m), 435 (m).. MS (ESI+): m/z (%) = 428 (100, $[M+H]^+$). HRMS (ESI+) calculated for $C_{22}H_{26}N_3O_4S$: 428.1639; found: 428.1650 and calculated for $C_{22}H_{25}N_3NaO_4S$: 450.1458; found: 450.1465.

N-(5-(5-(3-Hydroxyphenyl)thiophene-2-yl)-5-oxopentanoyl)-L-histidine (24b)

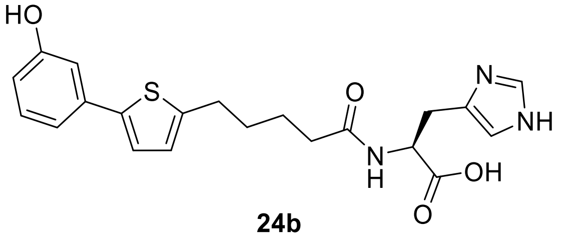

24b
[413.49]

The title compound was synthesised as described in the **general procedure 2A** with **23b** (258 mg, 0.60 mmol, 1.0 eq.) and KOH (453 mg, 8.01 mmol, 13.0 eq.) in MeOH (5 mL). 37 mg (0.18 mmol, 15%) of the desired product **24b** as colourless solid were obtained. Melting point: 189 C. ^{1}H-NMR: (DMSO-D$_6$, 400 MHz), δ [ppm] = 8.03 (d, ^{3}J = 7.9 Hz, 1H), 7.55 (s, 1H), 7.21 (d, ^{3}J = 3.5 Hz, 1H), 7.17 (t, ^{3}J = 7.9 Hz, 1H), 7.01 (d, ^{3}J = 8.2 Hz, 1H), 6.95 (t, ^{4}J = 2.1 Hz, 1H), 6.80 (s, 2H), 6.67 (dd, ^{3}J = 7.8 Hz, ^{3}J = 2.1 Hz, 1H), 4.44-4.39 (m, 1H), 2.93 (dd, ^{3}J = 4.8 Hz, ^{2}J = 14.7 Hz, 1H), 2.85-2.79 (m, 1H), 2.76 (t, ^{3}J = 6.9 Hz, 2H), 2.13 (t, ^{3}J = 6.9 Hz, 2H), 1.61-1.48 (m, 4H). ^{13}C-NMR: (DMSO-D$_6$, 100 MHz), δ [ppm] = 173.2 (1C), 171.9 (1C), 157.8 (1C), 157.8 (1C), 144.5 (1C), 140.8 (1C), 135.2 (1C), 134.7 (1C), 130.1 (1C), 125.6 (1C), 123.1 (1C), 115.9 (1C), 114.5 (1C), 114.3 (1C), 111.7 (1C), 52.2 (1C), 35.5 (1C), 34.7 (1C), 30.4 (1C), 29.2 (1C), 24.6 (1C). IR: ν [cm^{-1}] = 3239 (s), 1628 (m), 1591 (vs), 1549 (m), 1449 (m), 1477 (m), 1460 (m), 1445 (m), 1432 (s), 1396 (m), 1354 (m), 1313 (m), 1290 (m), 1249 (m), 1232 (m), 1217 (m), 1194 (m), 1161 (m), 1096 (w), 1047 (w), 1016 (w), 987 (w), 873 (m), 853 (m), 823 (m), 802 (s), 777 (m), 734 (m), 675 (s), 624 (s), 597 (m), 575 (m), 558 (s), 505 (m), 492 (m), 482 (m), 463 (m), 443 (m), 419 (m), 405 (m). MS (ESI+): m/z (%) = 414 (100, [M+H]$^{+}$). HRMS (ESI+) calculated for $C_{21}H_{24}N_3O_4S$: 414.1482; found: 414.1498.

Methyl *N*-(5-(5-(3-hydroxyphenyl)thiophene-2-yl)-5-oxopentanoyl)-L-valinate (25a)

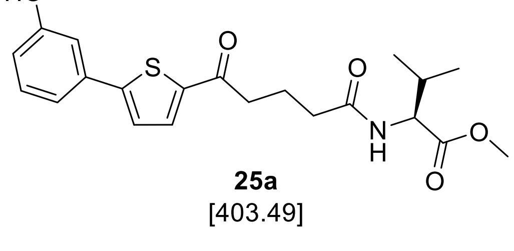

25a
[403.49]

The title compound was synthesised as described in the **general procedure 1A** with **2a** (200 mg, 0.69 mmol, 1.0 eq.), L-Val-OMe•HCl (116 mg, 0.69 mmol, 1.0 eq.), NEt_3 (0.30 mL, 2.10 mmol, 3.0 eq.), HOBt (141 mg, 1.04 mmol, 1.5 eq.) and EDC•HCl (198 mg, 1.04 mmol, 1.5 eq.) in DCM (50 mL). The crude product was purified *via* column chromatography (cyclohexane:EtOAc 1:2) and recrystallised of EtOAc. 127 mg (0.31 mmol, 45%) of the desired product **25a** as yellow solid were obtained. R_f = 0.58 (cyclohexane:EtOAc 1:2). Melting point: 101 C. ^{1}H-NMR: (DMSO-D_6, 400 MHz), δ [ppm] = 9.70 (s, 1H), 8.11 (d, ^3J = 8.1 Hz), 7.89 (d, ^3J = 4.0 Hz, 1H), 7.55 (d, ^3J = 4.0 Hz, 1H), 7.26 (t, ^3J = 7.9 Hz), 7.18 (d, ^3J = 7.7 Hz, 1H), 7.11 (t, ^4J = 1.9 Hz, 1H), 6.81 (ddd, ^3J = 8.0 Hz, ^4J = 2.3 Hz, ^4J = 1.0 Hz, 1H), 4.17 (t, ^3J = 7.6 Hz, 1H), 3.63 (s, 3H), 2.94 (t, ^3J = 7.2 Hz, 2H), 2.26 (t, ^3J = 7.3 Hz, 2H), 2.06-1.97 (m, 1H), 1.89-1.82 (m, 2H), 0.88 (d, ^3J = 6.9 Hz, 3H), 0.86 (d, ^3J = 6.9 Hz, 3H). ^{13}C-NMR: (DMSO-D_6, 400 MHz), δ [ppm] = 192.6 (1C), 172.2 (1C), 172.2 (1C), 158.0 (1C), 151.3 (1C), 142.2 (1C), 134.2 (1C), 133.9 (1C), 130.4 (1C), 124.8 (1C), 116.8 (1C), 116.3 (1C), 112.6 (1C), 57.4 (1C), 51.3 (1C), 37.3 (1C), 33.9 (2C), 29.7 (1C), 20.5 (1C), 18.9 (1C), 18.3 (1C). IR: v [cm^{-1}] = 3294 (m), 2961 (w), 2875 (m), 1741 (m), 1653 (m), 1631 (vs), 1596 (m), 1581 (m), 1527 (m), 1456 (m), 1436 (m), 1408 (w), 1372 (m), 1316 (m), 1287 (m), 1263 (m), 1241 (m), 1223 (s), 1201 (m), 1165 (m), 1151 (m), 1094 (w), 1058 (w), 1027 (w), 1004 (w), 995 (m), 928 (w), 866 (m), 808 (m), 781 (m), 748 (m), 685 (m), 585 (w), 536 (w), 510 (w), 434 (w). MS (ESI+): m/z (%) = 404 (40, $[M+H]^+$). HRMS (ESI+): calculated for $C_{21}H_{26}NO_5S$: 404.1526; found: 404.1518.

***N*-(5-(5-(3-Hydroxyphenyl)thiophene-2-yl)-5-oxopentanoyl)-L-valine (26a)**

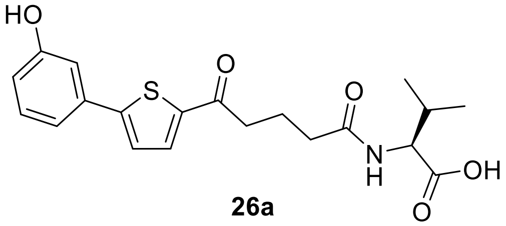

26a
[389.47]

The title compound was synthesised as described in the **general procedure 2A** with **25a** (166 mg, 0.41 mmol, 1.0 eq.) and KOH (69 mg, 1.23 mmol, 3.0 eq.) in MeOH (5 mL). 82 mg (0.21 mmol, 81%) of the desired product **26a** as yellow solid were obtained. Melting point: 143 °C. ^{1}H-NMR: (DMSO-D_6, 400 MHz), δ [ppm] = 12.49 (s, 1H), 9.71 (s, 1H), 7.96 (d, ^3J = 7.9 Hz), 7.89 (d, ^3J = 3.8 Hz, 1H), 7.54 (d, ^3J = 3.5 Hz,

1H), 7.26 (t, ^3J = 7.9 Hz, 1H), 7.18 (d, ^3J = 7.3 Hz, 1H), 7.11 (s, 1H), 6.82 (d, ^3J = 7.6 Hz, 1H), 4.17-4.13 (m, 1H), 2.95 (t, ^3J = 7.5 Hz, 2H), 2.26 (t, ^3J = 7.3 Hz, 2H), 2.08-2.01 (m, 1H), 1.86 (quin, ^3J = 6.8 Hz, 2H), 0.88 (d, ^3J = 6.6 Hz, 6H). ^{13}C-NMR: (DMSO-D$_6$, 100 MHz), δ [ppm] = 192.8 (1C), 173.3 (1C), 172.2 (1C), 158.0 (1C), 151.4 (1C), 142.3 (1C), 134.3 (1C), 130.5 (1C), 125.0 (1C), 124.9 (1C), 116.9 (1C), 114.5 (1C), 112.6 (1C), 57.2 (1C), 37.3 (1C), 34.0 (1C), 29.7 (2C), 20.6 (1C), 19.2 (1C), 18.1 (1C). IR: ν [cm^{-1}] = 3281 (w), 3216 (w), 3076 (w), 3011 (w), 2965 (w), 2937 (w), 2886 (w), 2834 (w), 2564 (w), 1717 (m), 1653 (m), 1630 (vs), 1595 (m), 1582 (m), 1527 (m), 1458 (s), 1437 (m), 1409 (w), 1373 (w), 1316 (w), 1289 (w), 1261 (w), 1240 (w), 1224 (m), 1165 (w), 1093 (w), 995 (w), 931 (w), 865 (w), 808 (w), 783 (m), 745 (m), 730 (w), 686 (m), 659 (w), 647 (w), 605 (w), 585 (w), 548 (w), 536 (w), 512 (w), 436 (w), 421 (w). MS (ESI+): m/z (%) = 390 (10, [M+H]$^+$), 412 (100, [M+Na]$^+$). HRMS (ESI+) calculated for $C_{20}H_{24}NO_5S$: 390.1370; found: 390.1376.

Methyl *N*-(5-(5-(3-hydroxyphenyl)thiophene-2-yl)pentanoyl)-L-valinate (25b)

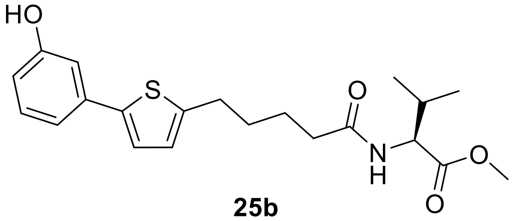

25b
[389.51]

The title compound was synthesised as described in the **general procedure 1A** with **2b** (200 mg, 0.72 mmol, 1.0 eq.), L-Val-OMe•HCl (121 mg, 0.72 mmol, 1.0 eq.), NEt_3 (0.30 mL, 2.16 mmol, 3.0 eq.), HOBt (146 mg, 1.08 mmol, 1.5 eq.) and EDC•HCl (208 mg, 1.08 mmol, 1.5 eq.) in DCM (50 mL). The crude product was purified *via* column chromatography (cyclohexane:EtOAc 1:1) and recrystallised of cyclohexane/EtOAc. 239 mg (0.61 mmol, 85%) of the desired product **25b** as yellow solid were obtained. R_f = 0.46 (cyclohexane:EtOAc 1:1). Melting point: 79 C. ^{1}H-NMR: (DMSO-D$_6$, 400 MHz), δ [ppm] = 9.49 (s, 1H), 8.08 (d, ^3J = 8.1 Hz), 7.21 (d, ^3J = 3.5 Hz, 1H), 7.17 (t, ^3J = 7.9 Hz, 1H), 7.00 (d, ^3J = 7.7 Hz, 1H), 6.95 (s, 1H), 6.81 (d, ^3J = 3.5 Hz, 1H), 6.67 (dd, ^3J = 8.1 Hz, ^4J = 2.3 Hz, 1H), 4.19-4.15 (m, 1H), 3.62 (s, 3H), 2.79 (t, ^3J = 6.9 Hz, 2H), 2.26-2.17 (m, 2H), 2.05-1.97 (m, 1H), 1.65-1.54 (m, 2H), 0.88 (d, ^3J = 7.2 Hz, 3H), 0.85 (d, ^3J = 7.5 Hz, 3H). ^{13}C-NMR: (DMSO-D$_6$, 400 MHz), δ [ppm] = 172.5 (1C), 172.3 (1C), 158.0 (1C), 144.5 (1C), 140.9 (1C), 135.2 (1C), 130.1

(1C), 125.6 (1C), 123.1 (1C), 115.8 (1C), 114.3 (1C), 111.7 (1C), 57.3 (1C), 51.6 (1C), 34.4 (1C), 30.6 (1C), 29.8 (1C), 29.1 (1C), 24.7 (1C), 19.0 (1C), 18.3 (1C). IR: ν [cm^{-1}] = 3319 (w), 3183 (w), 3078 (w), 2944 (m), 2871 (w), 1737 (m), 1648 (m), 1593 (m), 1529 (m), 1474 (m), 1461 (m), 1434 (s), 1416 (m), 1389 (w), 1370 (w), 1339 (w), 1313 (w), 1289 (m), 1271 (w), 1252 (w), 1231 (m), 1203 (vs), 1157 (s), 1087 (w), 1046 (w), 1016 (w), 996 (w), 984 (w), 968 (m), 944 (w), 910 (w), 852 (m), 806 (m), 779 (s), 732 (m), 690 (m), 624 (w), 598 (w), 573 (w), 560 (m), 534 (w), 507 (w), 443 (w). MS (ESI+): m/z (%) = 390 (100, $[M+H]^+$). HRMS (ESI+):calculated for $C_{21}H_{28}NO_4S$: 390.1734; found: 390.1735.

***N*-(5-(5-(3-Hydroxyphenyl)thiophene-2-yl)pentanoyl)-L-valine (26b)**

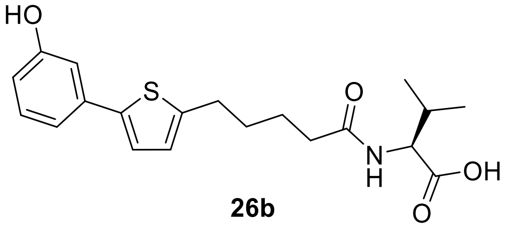

26b
[375.48]

The title compound was synthesised as described in the **general procedure 2B** with **25b** (155 mg, 0.40 mmol, 1.0 eq.) and 2M NaOH (2.0 mL) in EtOH (3 mL). The crude product was purified *via* column chromatography (cyclohexane:EtOAc 1:1 + 0.1% formic acid). 109 mg (0.29 mmol, 73%) of the desired product **26b** as brown oil were obtained. R_f = 0.31 (cyclohexane:EtOAc 1:1 + 0.1% formic acid). ^{1}H-NMR: (DMSO-D_6, 400 MHz), δ [ppm] = 12.45 (s, 1H), 9.50 (s, 1H), 7.92 (d, ^3J = 8.5 Hz, 1H), 7.21 (d, ^3J = 3.6 Hz, 1H), 7.17 (t, ^3J = 7.9 Hz, 1H), 7.00 (ddd, ^3J = 7.7 Hz, ^4J = 1.6 Hz, ^4J = 0.9 Hz, 1H), 6.95 (t, ^4J = 2.1 Hz, 1H), 6.81 (d, ^3J = 3.6 Hz, 1H), 6.67 (ddd, ^3J = 8.1 Hz, ^4J = 2.4 Hz, ^4J = 0.9 Hz, 1H), 4.14 (dd, ^3J = 8.6 Hz, ^3J = 5.9 Hz, 1H), 2.79 (t, ^3J = 7.1 Hz, 2H), 2.27-2.16 (m, 2H), 2.03 (dq, ^3J = 6.8 Hz, ^3J = 13.5 Hz, 1H), 1.65-1.54 (m, 2H), 0.88 (d, ^3J = 6.9 Hz, 3H), 0.87 (d, ^3J = 6.6 Hz, 3H). The spectrum shows double signals (6%). ^{13}C-NMR: (DMSO-D_6, 125 MHz), δ [ppm] = 173.1 (1C), 172.3 (1C), 157.7 (1C), 144.5 (1C), 140.8 (1C), 135.2 (1C), 130.0 (1C), 125.5 (1C), 123.0 (1C), 115.8 (1C), 114.3 (1C), 111.7 (1C), 57.0 (1C), 34.5 (1C), 30.5 (1C), 29.7 (1C), 29.1 (1C), 24.8 (1C), 19.1 (1C), 18.0 (1C). IR: ν [cm^{-1}] = 3310 (w), 3067 (w), 2964 (w), 2933 (w), 2873 (w), 1716 (s), 1611 (s), 1595 (s), 1580 (s), 1530 (vs), 1447 (s), 1392 (m), 1372 (m), 1215 (vs), 1183 (s), 1160 (s), 1043 (m), 991 (m), 857 (m), 841

(m), 802 (m), 777 (vs), 732 (m), 687 (m), 624 (m), 535 (m), 444 (m). MS (ESI+): m/z (%) = 376 (25, $[M+H]^+$), 398 (100, $[M+Na]^+$). HRMS (ESI+): calculated for $C_{20}H_{26}NO_4S$: 376.1577; found: 376.1572 and calculated for $C_{20}H_{25}NNaO_4S$: 398.1396; found: 398.1394.

1-(5-(3-Hydroxyphenyl)thiophene-2-yl)-5-(piperidine-1-yl)pentane-1,5-dione (27a)

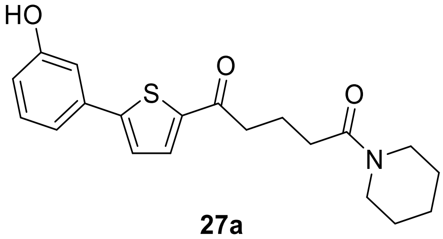

27a
[357.47]

The title compound was synthesised as described in the **general procedure 1A** with **2a** (300 mg, 1.03 mmol, 1.0 eq.), piperidine (0.1 mL, 1.03 mmol, 1.0 eq.), NEt_3 (0.43 mL, 3.09 mmol, 3.0 eq.), HOBt (209 mg, 1.55 mmol, 1.5 eq.) and EDC•HCl (297 mg, 1.55 mmol, 1.5 eq.) in DCM (50 mL). The crude product was purified *via* column chromatography (cyclohexane:EtOAc 1:3). 167 mg (0.44 mmol, 43%) of the desired product **27a** as colourless solid was obtained. R_f = 0.20 (cyclohexane:EtOAc 1:3). Melting point: 174-175 °C. ^{1}H-NMR: (DMSO-D_6, 400 MHz), δ [ppm] = 9.71 (s, 1H), 7.91 (d, ^3J = 4.1 Hz, 1H), 7.55 (d, ^3J = 4.1 Hz, 1H), 7.26 (t, ^3J = 7.9 Hz, 1H), 7.17-7.19 (m, 1H), 7.11 (s, 1H), 6.81 (d, ^3J = 8.9 Hz, 1H), 3.41-3.37 (m, 2H), 2.98 (t, ^3J = 7.2 Hz, 2H), 2.36 (t, ^3J = 7.3 Hz, 2H), 1.84 (quin, ^3J = 7.3 Hz, 2H), 1.59-1.40 (m, 8H). ^{13}C-NMR: (DMSO-D_6, 100 MHz), δ [ppm] = 192.9 (1C), 169.8 (1C), 157.9 (1C), 151.3 (1C), 142.2 (1C), 134.3 (1C), 133.9 (1C), 130.4 (1C), 124.9 (1C), 116.8 (1C), 116.3 (1C), 112.6 (1C), 45.8 (1C), 41.8 (1C), 37.5 (1C), 31.5 (1C), 26.0 (1C), 25.3 (1C), 24.0 (1C), 20.1 (1C). IR: ν [cm^{-1}] = 3101 (w), 2933 (w), 1652 (vs), 1586 (s), 1444 (vs), 1331 (w), 1274 (m), 1222 (s), 1199 (s), 1064 (w), 996 (w), 929 (w), 866 (m), 847 (m), 815 (m), 749 (s), 687 (m), 573 (w), 536 (w), 471 (w). MS (ESI-): m/z (%) = 356 (100, $[M-H]^-$). HRMS (ESI+) calculated for $C_{21}H_{25}N_2O_4S$: 358.1471; found: 358.1472.

5-(5-(3-Hydroxyphenyl)thiophene-2-yl)-1-(piperidine-1-yl)pentane-1-one (27b)

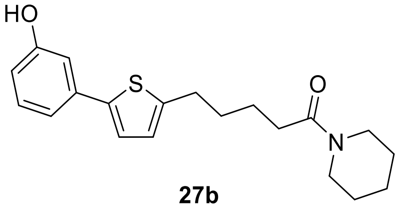

27b
[343.48]

The title compound was synthesised as described in the **general procedure 1A** with **2b** (100 mg, 0.36 mmol, 1.0 eq.), piperidine (0.04 mL, 0.36 mmol, 1.0 eq.), EDC•HCl (104 mg, 0.54 mmol, 1.5 eq.), HOBt (73.0 mg, 0.54 mmol, 1.5 eq.) and NEt_3 (0.15 mL, 1.08 mmol, 3.0 eq.) in DCM (25 mL). The crude product was purified *via* column chromatography (cyclohexane:EtOAc 1:1). 59 mg (0.17 mmol, 47%) of the desired product **27b** as colourless solid was obtained. R_f = 0.24 (cyclohexane:EtOAc 1:1). Melting point: 135 °C. ^{1}H-NMR: (DMSO-D_6, 400 MHz), δ [ppm] = 9.49 (s, 1H), 7.22 (d, ^3J = 3.6 Hz, 1H), 7.17 (m, 1H), 7.01 (d, ^3J = 9.0 Hz, 1H), 6.95 (brs, 1H), 6.82 (s, 1H), 6.67 (d, ^3J = 7.0 Hz,1H), 3.40-3.36 (m, 4H), 2.81-2.78 (m, 2H), 2.33-2.30 (m, 3H), 1.67-1.62 (m, 2H), 1.55 (brs, 3H), 1.44-1.39 (m, 4H). ^{13}C-NMR: (DMSO-D_6, 100 MHz), δ [ppm] = 170.0 (1C), 157.7 (1C), 144.5 (1C), 140.8 (1C), 135.2 (1C), 130.0 (1C), 125.6 (1C), 123.0 (1C), 115.8 (1C), 114.3 (1C), 111.7 (1C), 45.9 (1C), 41.8 (1C), 32.0 (1C), 30.7 (1C), 29.2 (1C), 26.1 (1C), 25.3 (1C), 24.3 (1C), 24.0 (1C). IR: ν [cm^{-1}] = 3102 (m), 3074 (m), 2923 (m), 2854 (m), 1735 (w), 1608 (m), 1584 (vs), 1513 (m), 1470 (s), 1445 (vs), 1414 (m), 1371 (m), 1346 (m), 1331 (m), 1304 (m), 1276 (m), 1253 (m), 1235 (s), 1225 (s), 1214 (s), 1166 (m), 1136 (m), 1126 (m), 1078 (w), 1053 (m), 1010 (m), 992 (m), 948 (w), 911 (w), 864 (m), 840 (m), 801 (m), 767 (vs), 682 (m), 627 (m), 601 (w), 579 (w), 543 (m), 532 (w), 497 (m), 481 (w), 446 (m), 426 (w), 403 (w). MS (ESI+): m/z (%) = 344 (100, $[M+H]^+$), 366 (10, $[M+Na]^+$). HRMS (ESI+) calculated for $C_{22}H_{26}NO_2S$: 344.1679; found: 344.1678.

1-(5-(5-(3-Hydroxyphenyl)thiophene-2-yl)-5-oxopentanoyl)piperidine-4-carboxamide (28a)

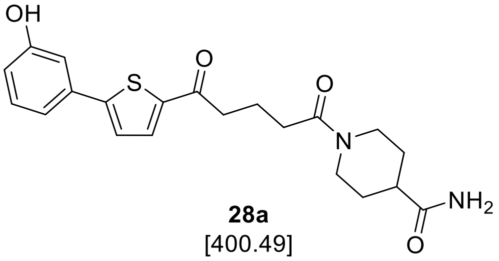

28a
[400.49]

The title compound was synthesised as described in the **general procedure 1A** with **2a** (300 mg, 1.03 mmol, 1.0 eq.), piperidine-4-carboxamide (132 mg, 1.03 mmol, 1.0 eq.), NEt_3 (0.43 mL, 3.09 mmol, 3.0 eq.), HOBt (209 mg, 1.55 mmol, 1.5 eq.) and EDC•HCl (297 mg, 1.55 mmol, 1.5 eq.) in DCM (50 mL). The crude product was purified *via* column chromatography (DCM:MeOH 10:1). 129 mg (0.32 mmol, 31%) of the desired product **28a** as colourless solid was obtained. R_f = 0.14 (DCM:MeOH 10:1). Melting point: 184-185 °C. ^{1}H-NMR: (DMSO-D_6, 400 MHz), δ [ppm] = 9.71 (s, 1H), 7.92 (d, ^3J = 3.7 Hz, 1H), 7.55 (d, ^3J = 3.9 Hz, 1H), 7.28-7.11 (m, 4H), 6.83-6.76 (m, 2H), 4.33-4.36 (m, 1H), 3.83-3.87 (m, 1H), 3.02-2.97 (m, 3H), 2.60-2.32 (m, 4H), 1.84 (quin, ^3J = 7.0 Hz, 2H), 1.74-1.68 (m, 2H), 1.49-1.30 (m, 2H). ^{13}C-NMR: (DMSO-D_6, 100 MHz), δ [ppm] = 192.9 (1C), 176.0 (1C), 169.9 (1C), 158.0 (1C), 151.3 (1C), 142.2 (1C), 134.3 (1C), 133.9 (1C), 130.4 (1C), 124.9 (1C), 116.9 (1C), 116.3 (1C), 112.6 (1C), 44.5 (1C), 41.5 (1C), 40.6 (1C), 37.6 (1C), 31.6 (1C), 28.9 (1C), 28.2 (1C), 20.1 (1C). IR: ν [cm^{-1}] = 3422 (w), 3331 (w), 3210 (w), 2948 (w), 1652 (vs), 1585 (s), 1444 (vs), 1331 (m), 1274 (m), 1199 (s), 1167 (w), 1057 (w), 1025 (m), 917 (w), 873 (s), 822 (m), 774 (vs), 745 (m), 686 (m), 573 (m), 526 (s). MS (ESI+): m/z (%) = 401 (30, $[M+H]^+$). HRMS (ESI+) calculated for $C_{21}H_{25}N_2O_4S$: 401.1530; found: 401.1518.

1-(5-(5-(3-Hydroxyphenyl)thiophene-2-yl)pentanoyl)piperidine-4-carboxamide (28b)

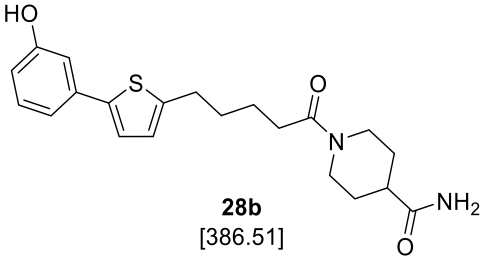

28b
[386.51]

The title compound was synthesised as described in the **general procedure 1A** with **2b** (100 mg, 0.36 mmol, 1.0 eq.), piperidine-4-carboxamide (46.0 mg, 0.36 mmol, 1.0 eq.), EDC•HCl (104 mg, 0.54 mmol, 1.5 eq.), HOBt (73.0 mg, 0.54 mmol, 1.5 eq.) and NEt_3 (0.15 mL, 1.08 mmol, 3.0 eq.) in DCM (25 mL). The crude product was purified *via* column chromatography (DCM:MeOH 20:1) and recrystallised from isopropanol. 52 mg (0.13 mmol, 36%) of the desired product **28b** as yellow solid was obtained. R_f = 0.12 (DCM:MeOH 20:1). Melting point: 199 °C. ^{1}H-NMR: (DMSO-D_6, 400 MHz), δ [ppm] = 9.51 (s, 1H), 7.24 (s, 1H), 7.21 (d, ^3J = 3.5 Hz, 1H), 7.17 (t, ^3J = 7.9 Hz, 1H), 7.01 (d, ^3J = 8.0 Hz, 1H), 6.95 (s, 1H), 6.82 (d, ^3J = 3.2 Hz, 1H), 6.75 (s, 1H), 6.67 (dd, ^3J = 7.6 Hz, ^4J = 1.9 Hz, 1H), 4.34 (d, ^2J = 11.9 Hz, 1H), 3.85 (d, ^2J = 13.2 Hz, 1H), 2.98 (t, ^2J = 12.7 Hz, 1H), 2.80 (t, ^3J = 7.4 Hz, 2H), 2.58-2.55 (m, 1H), 2.35-2.28 (m, 3H), 1.72-1.63 (m, 4H), 1.59-1.54 (m, 2H), 1.49-1.27 (m, 2H). ^{13}C-NMR: (DMSO-D_6, 100 MHz), δ [ppm] = 176.0 (1C), 170.2 (1C), 157.8 (1C), 144.6 (1C), 140.9 (1C), 135.2 (1C), 130.1 (1C), 125.7 (1C), 123.1 (1C), 115.9 (1C), 114.3 (1C), 111.7 (1C), 44.6 (1C), 41.5 (1C), 40.6 (1C), 32.0 (1C), 30.8 (1C), 29.3 (1C), 29.0 (1C), 28.2 (1C), 24.3 (1C). IR: ν [cm^{-1}] = 3412 (m), 3311 (w), 3199 (w), 3072 (w), 2942 (m), 2859 (w), 1663 (s), 1636 (m), 1606 (s), 1585 (vs), 1515 (w), 1474 (m), 1454 (s), 1428 (m), 1416 (m), 1373 (m), 1350 (m), 1332 (m), 1311 (w), 1271 (m), 1242 (s), 1222 (m), 1209 (s), 1173 (m), 1129 (w), 1104 (w), 1080 (w), 1053 (w), 1027 (m), 993 (w), 952 (w), 933 (w), 916 (m), 867 (m), 838 (m), 815 (m), 802 (m), 772 (vs), 684 (m), 661 (w), 602 (m), 542 (m), 517 (w), 499 (w), 488 (w), 468 (w), 459 (w), 446 (m). MS (ESI+): m/z (%) = 387 (100, [M+H]$^+$), 409 (40, [M+Na]$^+$). HRMS (ESI+) calculated for $C_{21}H_{27}N_2O_3S$: 387.1737; found: 387.1736.

Ethyl 1-(5-(5-(3-hydroxyphenyl)thiophene-2-yl)-5-oxopentanoyl)piperidine-4-carboxylate (29a)

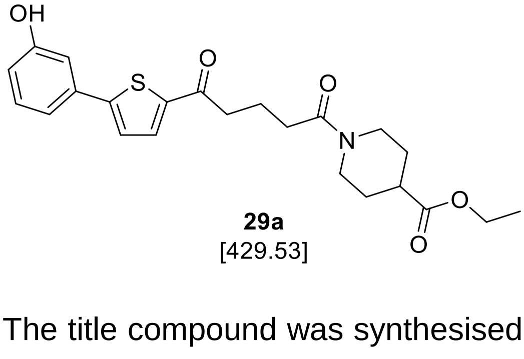

29a
[429.53]

The title compound was synthesised as described in the **general procedure 1A** with **2a** (600 mg, 2.07 mmol, 1.0 eq.), ethyl piperidine-4-carboxylate (0.3 mL, 2.07 mmol,

1.0 eq.), NEt_3 (0.9 mL, 6.20 mmol, 3.0 eq.), HOBt (420 mg, 3.11 mmol, 1.5 eq.) und EDC•HCl (595 mg, 3.11 mmol, 1.5 eq.) in DCM (100 mL). The crude product was purified *via* column chromatography (cyclohexane:EtOAc 1:5). 395 mg (0.92 mmol, 45%) of the desired product **29a** as pale-yellow solid was obtained. R_f = 0.20 (cyclohexane:EtOAc 1:5). Melting point: 157-158 °C. ^{1}H-NMR: (DMSO-D_6, 400 MHz), δ [ppm] = 9.70 (s, 1H), 7.91 (d, ^3J = 4.1 Hz, 1H), 7.55 (d, ^3J = 3.9 Hz, 1H), 7.26 (t, ^3J = 7.9 Hz, 1H), 7.18 (d, ^3J = 7.8 Hz, 1H), 7.11 (s, 1H), 6.82 (dd, ^3J = 8.9 Hz, ^4J = 1.6 Hz, 1H), 4.23 (d, ^2J = 13.5 Hz, 1H), 4.06 (q, ^3J = 7.1 Hz, 2H), 3.79 (d, ^2J = 13.7 Hz, 1H), 3.08 (t, ^2J = 11.4 Hz, 1H), 2.98 (t, ^3J = 7.2 Hz, 2H), 2.72 (t, ^2J = 11.5 Hz, 1H), 2.61-2.55 (m, 1H), 2.36-2.41 (m, 2H), 1.87-1.80 (m, 4H), 1.32-1.52 (m, 2H), 1.78 (t, ^3J = 7.1 Hz, 3H). ^{13}C-NMR: (DMSO-D_6, 100 MHz), δ [ppm] = 192.9 (1C), 173.9 (1C), 170.0 (1C), 158.0 (1C), 151.3 (1C), 142.2 (1C), 134.3 (1C), 133.9 (1C), 130.4 (1C), 124.9 (1C), 116.8 (1C), 116.3 (1C), 112.6 (1C), 60.0 (1C), 44.0 (1C), 40.2 (1C), 40.1 (1C), 37.5 (1C), 31.5 (1C), 28.3 (1C), 27.6 (1C), 20.0 (1C), 14.1 (1C). IR: ν [cm^{-1}] = 3477 (w), 3114 (w), 2974 (w), 1723 (vs), 1650 (vs), 1618 (m), 1590 (s), 1443 (s), 1376 (m), 1312 (w), 1271 (m), 1184 (s), 1040 (m), 936 (w), 872 (m), 845 (m), 765 (s), 687 (m), 528 (w), 503 (s), 471 (m). MS (ESI+): m/z (%) = 430 (100, $[M+H]^+$). HRMS (ESI+) calculated for $C_{23}H_{28}NO_5S$: 430.1683; found: 430.1676.

Ethyl 1-(5-(5-(3-hydroxyphenyl)thiophene-2-yl)pentanoyl)piperidine-4-carboxylate (29b)

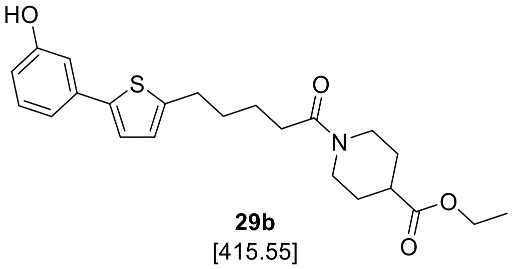

29b
[415.55]

The title compound was synthesised as described in the **general procedure 1A** with **2b** (200 mg, 0.72 mmol, 1.0 eq.), ethyl piperidine-4-carboxylate (0.11 mL, 0.72 mmol, 1.0 eq.), EDC•HCl (208 mg, 1.08 mmol, 1.5 eq.), HOBt (146 mg, 1.08 mmol, 1.5 eq.) and NEt_3 (0.30 mL, 2.16 mmol, 3.0 eq.) in DCM (50 mL). The crude product was purified *via* column chromatography (cyclohexane:EtOAc 1:1). 141 mg (0.34 mmol, 47%) of the desired product **29b** as colourless solid was obtained. R_f = 0.30 (cyclohexane:EtOAc 1:1). Melting point: 103 °C. ^{1}H-NMR: (DMSO-D_6, 400 MHz), δ

[ppm] = 9.50 (s, 1H), 7.21 (d, ^3J = 3.6 Hz, 1H), 7.1 (t, ^3J = 6.0 Hz, 1H), 7.00 (d, ^3J = 7.8 Hz, 1H), 6.95 (t, ^3J = 1.9 Hz, 1H), 6.82 (d, ^3J = 3.7 Hz, 1H), 6.67 (ddd, ^3J = 8.1 Hz, ^4J = 2.4 Hz, ^4J = 0.9 Hz, 1H), 4.21 (d, ^2J = 12.8 Hz, 1H), 4.06 (q, ^3J = 7.1 Hz, 2H), 3.79 (d, ^2J = 13.3 Hz, 1H), 3.06 (t, ^2J = 12.6 Hz, 1H), 2.80 (t, ^3J = 7.2 Hz, 2H), 2.69 (t, ^2J = 11.1 Hz, 1H), 2.60-2.53 (m, 1H), 2.34 (dt, ^3J = 7.3 Hz, ^4J = 1.9 Hz, 2H), 1.81 (t, ^3J = 10.5 Hz, 2H), 1.68-1.30 (m, 6H), 1.17 (t, ^3J = 7.1 Hz, 3H). ^{13}C-NMR: (DMSO-D$_6$, 100 MHz), δ [ppm] = 173.9 (1C), 170.3 (1C), 157.8 (1C), 144.5 (1C), 140.9 (1C), 135.2 (1C), 130.1 (1C), 125.6 (1C), 123.1 (1C), 115.8 (1C), 114.3 (1C), 111.7 (1C), 60.0 (2C), 44.1 (1C), 40.1 (1C), 32.0 (1C), 30.7 (1C), 29.3 (1C), 28.4 (1C), 27.7 (1C), 24.3 (1C), 14.1 (1C). IR: ν [cm^{-1}] = 3084 (w), 3024 (w), 2946 (m), 2932 (m), 2899 (w), 2865 (w), 2740 (w), 1718 (s), 1612 (m), 1592 (vs), 1550 (m), 1515 (w), 1475 (m), 1455 (vs), 1444 (vs), 1413 (m), 1371 (m), 1341 (w), 1294 (s), 1270 (m), 1239 (m), 1226 (s), 1196 (vs), 1173 (s), 1161 (s), 1098 (m), 1039 (m), 1005 (m), 986 (m), 952 (m), 909 (w), 864 (m), 857 (m), 800 (m), 778 (vs), 755 (s), 687 (m), 624 (w), 594 (w), 546 (w), 497 (w), 483 (w), 447 (w). MS (ESI+): m/z (%) = 416 (100, [M+H]$^+$), 438 (15, [M+Na]$^+$). HRMS (ESI+) calculated for $C_{23}H_{30}NO_4S$: 416.1890; found: 416.1890.

1-(5-(5-(3-Hydroxyphenyl)thiophene-2-yl)-5-oxopentanoyl)piperidine-4-carboxylic acid (30a)

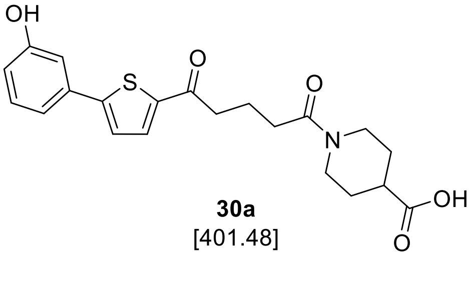

30a
[401.48]

The title compound was synthesised as described in the **general procedure 2A** with **29a** (200 mg, 0.46 mmol, 1.0 eq.) and KOH (78.2 mg, 1.39 mmol, 3.0 eq.) in MeOH (3.0 mL). 104 mg (0.26 mmol, 57%) of the desired product **30a** as colourless solid was obtained. Melting point: 185-190 °C. ^{1}H-NMR: (DMSO-D$_6$, 400 MHz), δ [ppm] = 12.22 (s, 1H), 9.70 (s, 1H), 7.91 (d, ^3J = 3.7 Hz, 1H), 7.54 (d, ^3J = 3.7 Hz, 1H), 7.26 (t, ^3J = 7.9 Hz, 1H), 7.18 (d, ^3J = 7.6 Hz, 1H), 7.11 (s, 1H), 6.81 (d, ^3J = 8.0 Hz, 1H), 4.20-4.24 (m, 1H), 3.77-3.80 (m, 1H), 3.04-3.10 (m, 1H), 2.98 (t, ^3J = 7.1 Hz, 2H), 2.67-2.74 (m, 1H), 2.50-2.45 (m, 1H), 2.38 (t, ^3J = 7.1 Hz, 2H), 1.85-1.80 (m, 4H), 1.51-1.32 (m, 2H). ^{13}C-NMR: (DMSO-D$_6$, 100 MHz), δ [ppm] = 193.4 (1C), 176.2 (1C),

170.5 (1C), 158.5 (1C), 151.8 (1C), 142.7 (1C), 134.8 (1C), 134.4 (1C), 131.0 (1C), 125.4 (1C), 117.4 (1C), 116.8 (1C), 113.1 (1C), 44.7 (1C), 40.9 (1C), 40.6 (1C), 38.1 (1C), 32.1 (1C), 28.9 (1C), 28.3 (1C), 20.6 (1C). IR: v [cm^{-1}] = 3165 (m), 2592 (w), 1734 (vs), 1651 (vs), 1575 (s), 1445 (s), 1369 (m), 1304 (m), 1229 (s), 1173 (vs), 1030 (m), 929 (m), 872 (m), 776 (s), 724 (m), 681 (m), 626 (m), 530 (m). MS (ESI+): m/z (%) = 402 (100, [M+H]$^+$), 424 (30, [M+Na]$^+$). HRMS (ESI+) calculated for $C_{21}H_{24}NO_5S$: 402.1370; found: 402.1365.

1-(5-(5-(3-Hydroxyphenyl)thiophene-2-yl)pentanoyl)piperidine-4-caboxylic acid (30b)

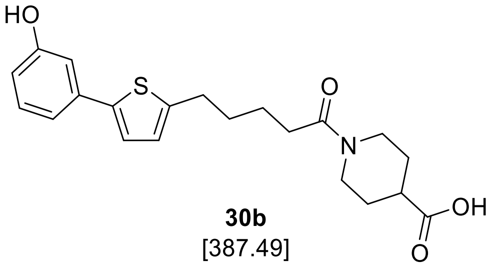

30b
[387.49]

The title compound was synthesised as described in the **general procedure 2A** with **29b** (83 mg, 0.2 mmol, 1.0 eq.) and KOH (11 mg, 0.6 mmol, 3.0 eq.) in MeOH (2 mL). 69 mg (0.18 mmol, 90%) of the desired product **30b** as colourless solid was obtained. Melting point: 186 °C. ^{1}H-NMR: (DMSO-D$_6$, 400 MHz), δ [ppm] = 12.22 (s, 1H), 9.50 (s, 1H), 7.21 (d, ^3J = 3.5 Hz, 1H), 7.17 (t, ^3J = 7.9 Hz, 1H), 7.01 (d, ^3J = 7.7 Hz, 1H), 6.95 (s, 1H), 6.82 (d, ^3J = 3.5 Hz, 1H), 6.67 (dd, ^3J = 7.9 Hz, ^4J = 2.1 Hz, 1H), 4.20 (d, ^2J = 12.6 Hz, 1H), 3.79 (d, ^2J = 15.0 Hz, 1H), 3.06 (t, ^2J = 11.8 Hz, 1H), 2.80 (t, ^3J = 7.2 Hz, 2H), 2.70 (t, ^2J = 11.5 Hz, 1H), 2.35-2.32 (m, 1H), 2.33 (t, ^3J = 7.3 Hz, 2H), 1.80 (t, ^3J = 11.3 Hz, 2H), 1.65 (quin, ^3J = 7.2 Hz, 2H), 1.55 (quin, ^3J = 7.5 Hz, 2H), 1.50-1.29 (m, 2H). ^{13}C-NMR: (DMSO-D$_6$, 100 MHz), δ [ppm] = 175.7 (1C), 170.3 (1C), 157.8 (1C), 144.6 (1C), 140.9 (1C), 135.2 (1C), 130.1 (1C), 125.6 (1C), 123.1 (1C), 115.9 (1C), 114.3 (1C), 111.7 (1C), 44.3 (1C), 40.3 (1C), 32.0 (1C), 30.7 (1C), 29.3 (1C), 28.5 (1C), 27.8 (1C), 24.3 (1C). One ^{13}C-signal lies under the DMSO peak. IR: v [cm^{-1}] = 3081 (m), 3021 (m), 2948 (m), 2933 (m), 2916 (m), 2866 (m), 2736 (w), 1694 (vs), 1619 (m), 1593 (vs), 1550 (m), 1516 (m), 1475 (m), 1455 (vs), 1412 (m), 1368 (m), 1294 (m), 1268 (m), 1228 (s), 1206 (m), 1179 (m), 1108 (w), 1082 (w), 1024 (m), 1009 (m), 987 (m), 954 (m), 920 (m), 910 (m), 863 (m), 857 (m), 800 (m), 777 (s), 759 (s), 729 (m), 687 (m), 623 (w), 593 (w), 542 (w), 525 (m), 494 (w), 469 (w), 443 (w).

MS (ESI+): m/z (%) = 388 (100, $[M+H]^+$), 405 (5, $[M+NH_4]^+$), 410 (10, $[M+Na]^+$). HRMS (ESI+) calculated for $C_{21}H_{26}NO_4S$: 388.1577; found: 388.1574.

1-(5-(3-Hydroxyphenyl)thiophene-2-yl)-5-(4-(methylsulfonyl)piperazine-1-yl)pentan-1,5-dione (31a)

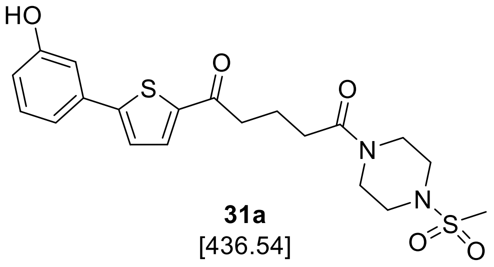

31a
[436.54]

The title compound was synthesised as described in the **general procedure 1A** with **2a** (400 mg, 1.38 mmol, 1.0 eq.), 1-methylsulfonyl-piperazine (227 mg, 1.38 mmol, 1.0 eq.), NEt_3 (0.6 mL, 4.14 mmol, 3.0 eq.), HOBt (280 mg, 2.07 mmol, 1.5 eq.) and EDC•HCl (397 mg, 2.07 mmol, 1.5 eq.) in DCM (25 mL). The crude product was purified *via* column chromatography (cyclohexane:EtOAc 3:1 → DCM:MeOH 10:1). 232 mg (0.53 mmol, 39%) of the desired product **31a** as colourless solid was obtained. R_f = 0.36 (DCM:MeOH 10:1). Melting point: 181-182 °C. ^{1}H-NMR: (DMSO-D_6, 400 MHz), δ [ppm] = 9.67 (s, 1H), 7.88 (d, ^3J = 4.1 Hz, 1H), 7.52 (d, ^3J = 4.1 Hz, 1H), 7.23 (t, ^3J = 7.8 Hz, 1H), 7.15 (d, ^3J = 8.2 Hz, 1H), 7.07 (s, 1H), 6.78 (dd, ^3J = 8.0 Hz, ^4J = 2.3 Hz, 1H), 3.58-3.46 (m, 4H), 3.09-3.04 (m, 4H), 2.96 (t, ^3J = 7.2 Hz, 2H), 2.84 (s, 3H), 2.39 (t, ^3J = 7.3 Hz, 2H), 1.83 (quin, ^3J = 7.2 Hz, 2H). ^{13}C-NMR: (DMSO-D_6, 100 MHz), δ [ppm] = 193.3 (1C), 170.9 (1C), 158.5 (1C), 151.9 (1C), 142.7 (1C), 134.8 (1C), 134.4 (1C), 130.9 (1C), 125.4 (1C), 117.4 (1C), 116.8 (1C), 113.1 (1C), 46.1 (1C), 45.7 (1C), 44.9 (1C), 41.1 (1C), 38.0 (1C), 34.7 (1C), 32.0 (1C), 20.4 (1C). IR: ν [cm^{-1}] = 3118 (w), 2844 (w), 1682 (m), 1646 (s), 1593 (s), 1475 (w), 1446 (s), 1324 (vs), 1269 (s), 1239 (m), 1173 (vs), 1052 (m), 964 (s), 904 (s), 816 (m), 773 (s), 678 (m), 560 (w), 511 (vs), 461 (w). MS (ESI+): m/z (%) = 459 (100, $[M+Na]^+$). HRMS (ESI+) calculated for $C_{20}H_{24}N_2O_5S_2Na$: 459.1019; found: 459.1026.

5-(5-(3-Hydroxyphenyl)thiophene-2-yl)-1-(4-(methylsulfonyl)piperazine-1-yl)pentane-1-one (31b)

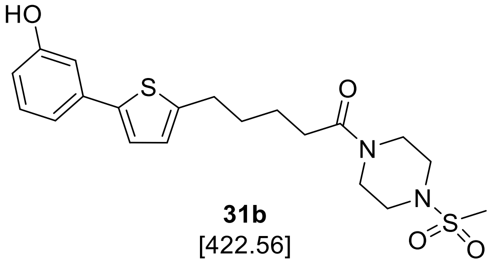

31b
[422.56]

As described in the **general procedure 1A** with **2b** (200 mg, 0.72 mmol, 1.0 eq.), 1-methylsulfonyl-piperazine (119 mg, 0.79 mmol, 1.0 eq.), NEt_3 (0.3 mL, 2.16 mmol, 3.0 eq.), HOBt (207 mg, 1.08 mmol, 1.5 eq.) and EDC•HCl (146 mg, 1.08 mmol, 1.5 eq.) in DCM (50 mL). The crude product was purified *via* column chromatography (EtOAc). 170 mg (0.40 mmol, 56%) of the desired product **31b** as colourless solid was obtained. R_f = 0.23 (EtOAc). Melting point: 178-182 °C. ^{1}H-NMR: (DMSO-D_6, 400 MHz), δ [ppm] = 9.50 (s, 1H), 7.22 (d, ^3J = 3.7 Hz, 1H), 7.15-7.19 (m, 1H), 7.01 (d, ^3J = 7.8 Hz, 1H), 6.95 (t, ^4J = 2.0 Hz, 1H), 6.82 (d, ^3J = 3.7 Hz, 1H), 6.67 (dd, ^3J = 8.0 Hz, ^4J = 2.3 Hz, 1H), 3.55 (t, ^3J = 5.2 Hz, 4H), 3.10-3.00 (m, 4H), 2.86 (s, 3H), 2.81 (t, ^3J = 7.3 Hz, 2H), 2.37 (t, ^3J = 7.2 Hz, 2H), 1.62-1.54 (m, 4H). ^{13}C-NMR: (DMSO-D_6, 100 MHz), δ [ppm] = 170.7 (1C), 157.8 (1C), 144.5 (1C), 140.9 (1C), 135.2 (1C), 130.1 (1C), 125.7 (1C), 123.1 (1C), 115.9 (1C), 114.3 (1C), 111.7 (1C), 45.6 (1C), 45.3 (1C), 44.4 (1C), 40.5 (1C), 34.0 (1C), 31.9 (1C), 30.7 (1C), 29.3 (1C), 24.1 (1C). IR: ν [cm^{-1}] = 3097 (w), 3012 (w), 2935 (w), 2850 (w), 1728 (w), 1617 (s), 1590 (vs), 1547 (w), 1506 (w), 1477 (s), 1446 (vs), 1343 (vs), 1326 (vs), 1295 (m), 1276 (m), 1230 (s), 1211 (m), 1170 (vs), 1112 (m), 1057 (m), 1026 (w), 1011 (w), 987 (m), 963 (s), 939 (s), 911 (s), 855 (s), 803 (m), 777 (vs), 747 (s), 685 (s), 623 (m), 592 (w), 562 (w), 524 (s), 510 (vs), 471 (m), 455 (m). MS (ESI-): m/z (%) = 421 (100, $[M-H]^-$). HRMS (ESI-) calculated for $C_{20}H_{25}N_2O_4S_2$: 421.1261; found: 421.1259.

1-(4-(Cyclopropylcarbonyl)piperazine-1-yl)-5-(5-(3-hydroxyphenyl)thiophene-2-yl)pentan-1,5-dione (32a)

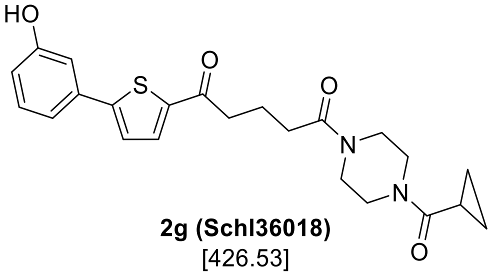

2g (Schl36018)
[426.53]

The title compound was synthesised as described in the **general procedure 1A** with **2a** (400 mg, 1.38 mmol, 1.0 eq.), cyclopropyl(piperazin-1-yl)ketone (263 mg, 1.38 mmol, 1.0 eq.), NEt_3 (0.6 mL, 4.14 mmol, 3.0 eq.), HOBt (280 mg, 2.07 mmol, 1.5 eq.) and EDC•HCl (397 mg, 2.07 mmol, 1.5 eq.) in DCM (50 mL). The crude product was purified *via* column chromatography (DCM:MeOH 20:1). 476 mg (1.12 mmol, 81%) of the desired product **32a** as pale-yellow solid was obtained. R_f = 0.16 (DCM:MeOH 10:1). Melting point: 149-152 °C. ^{1}H-NMR: (DMSO-D_6, 400 MHz), δ [ppm] = 9.71 (s, 1H), 7.92 (d, ^3J = 4.1 Hz, 1H), 7.55 (d, ^3J = 3.9 Hz, 1H), 7.26 (t, ^3J = 7.8 Hz, 1H), 7.18 (d, ^3J = 7.8 Hz, 1H), 7.11 (s, 1H), 6.83-6.80 (m, 1H), 3.68-3.65 (m, 4H), 3.48-3.44 (m, 4H), 3.00 (t, ^3J = 7.2 Hz, 2H), 2.42 (t, ^3J = 7.2 Hz, 2H), 1.99-1.93 (m, 1H), 1.87 (quin, ^3J = 7.2 Hz, 2H), 0.77-0.69 (m, 4H). ^{13}C-NMR: (DMSO-D_6, 100 MHz), δ [ppm] = 193.4 (1C), 171.8 (1C), 171.0 (1C), 158.5 (1C), 151.8 (1C), 142.7 (1C), 134.8 (1C), 134.4 (1C), 131.0 (1C), 125.4 (1C), 117.4 (1C), 116.8 (1C), 113.1 (1C), 45.4 (1C), 45.1 (1C), 42.2 (1C), 41.8 (1C), 38.0 (1C), 32.0 (1C), 20.4 (1C), 10.9 (1C), 7.6 (2C). IR: ν [cm^{-1}] = 3497 (w), 3151 (w), 2893 (w), 1692 (m), 1633 (vs), 1593 (vs), 1435 (vs), 1367 (w), 1288 (m), 1230 (vs), 1003 (s), 973 (m), 924 (w), 867 (s), 796 (m), 743 (vs), 678 (s), 530 (s), 471 (m). MS (ESI+): m/z (%) = 449 (100, $[M+Na]^+$). HRMS (ESI+) calculated for $C_{23}H_{27}N_2O_4S$: 427.1686; found: 427.1691.

1-(4-(Cyclopropylcarbonyl)piperazine-1-yl)-5-(5-(3-hydroxyphenyl)thiophene-2-yl)pentan-1-one (32b)

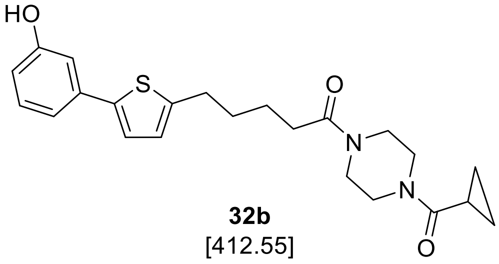

32b
[412.55]

The title compound was synthesised as described in the **general procedure 1A** with **2b** (100 mg, 0.36 mmol, 1.0 eq.), cyclopropylcarbonylpiperazine (0.15 mL, 0.36 mmol, 1.0 eq.), EDC•HCl (104 mg, 0.54 mmol, 1.5 eq.), HOBt (73 mg, 0.54 mmol, 1.5 eq.) and NEt_3 (0.15 mL, 1.08 mmol, 3.0 eq.) in DCM (25 mL). The crude product was purified *via* column chromatography (DCM:MeOH 20:1) and recrystallised of EtOAc. 87 mg (0.21 mmol, 58%) of the desired product **32b** as yellow solid was obtained. R_f = 0.40 (DCM:MeOH 20:1). Melting point: 115 °C. ^{1}H-NMR: (DMSO-D_6, 400 MHz), δ [ppm] = 9.51 (s, 1H), 7.22 (d, ^3J = 3.4 Hz, 1H), 7.17 (t, ^3J = 7.9 Hz, 1H), 7.00 (d, ^3J = 6.9 Hz, 1H), 6.95 (t, ^3J = 3.7 Hz, 1H), 6.83 (d, ^3J = 3.7 Hz, 1H), 6.67 (dd, ^3J = 8.2 Hz, ^4J = 1.4 Hz, 1H), 3.64-3.43 (m, 8H), 2.81 (t, ^3J = 7.1 Hz, 2H), 2.38 (t, ^3J = 7.1 Hz, 2H), 1.96-1.91 (m, 1H), 1.67 (quin, ^3J = 7.3 Hz, 2H), 1.58 (quin, ^3J = 7.3 Hz, 2H), 0.76-0.68 (m, 4H). ^{13}C-NMR: (DMSO-D_6, 100 MHz), δ [ppm] = 171.3 (1C), 170.8 (1C), 157.8 (1C), 144.5 (1C), 140.8 (1C), 135.2 (1C), 130.1 (1C), 125.7 (1C), 123.1 (1C), 115.8 (1C), 114.3 (1C), 111.7 (1C), 45.1 (1C), 44.7 (1C), 41.7 (1C), 41.3 (1C), 32.0 (1C), 30.7 (1C), 29.3 (1C), 24.1 (1C), 10.4 (1C), 7.1 (2C). IR: ν [cm^{-1}] = 3096 (w), 3009 (w), 2945 (w), 2914 (w), 2898 (w), 1638 (s), 1616 (s), 1593 (s), 1550 (w), 1514 (w), 1475 (m), 1438 (vs), 1369 (w), 1294 (m), 1227 (s), 1165 (m), 1088 (w), 1049 (w), 1030 (m), 1012 (m), 987 (m), 941 (w), 910 (w), 865 (m), 801 (m), 777 (s), 688 (m), 643 (w), 624 (w), 574 (w), 536 (m), 505 (w), 448 (m). MS (ESI+): m/z (%) = 413 (27, $[M+H]^+$), 430 (25, $[M+NH_4]^+$), 435 (20, $[M+Na]^+$). HRMS (ESI+) calculated for $C_{23}H_{29}N_2O_3S$: 413.1893; found: 413.1907 and calculated for $C_{23}H_{28}N_2NaO_3S$: 435.1713; found: 435.1718.

1-(4-(2-Furoylpiperazine-1-yl)-5-(5-(3-hydroxyphenyl)thiophene-2-yl)pentane-1,5-dione (33a)

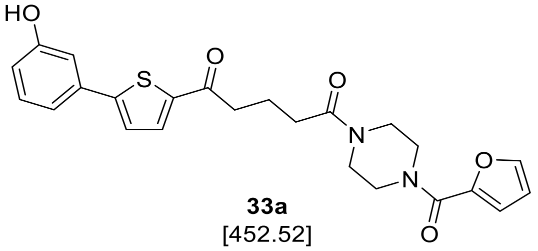

33a
[452.52]

The title compound was synthesised as described in the **general procedure 1A** with **2a** (200 mg, 0.69 mmol, 1.0 eq.), furan-2-yl-piperazinyl-methanone (124 mg, 0.69 mmol, 1.0 eq.), NEt_3 (0.3 mL, 2.07 mmol, 3.0 eq.), HOBt (140 mg, 1.04 mmol, 1.5 eq.) and EDC•HCl (199 mg, 1.04 mmol, 1.5 eq.) in DCM (50 mL). The crude product was purified *via* column chromatography (DCM:MeOH 10:1). 218 mg (0.48 mmol, 70%) of the desired product **33a** as yellow solid was obtained. R_f = 0.50 (DCM:MeOH 10:1). Melting point: 160-163 °C. ^{1}H-NMR: (DMSO-D_6, 400 MHz), δ [ppm] = 9.71 (s, 1H), 7.93 (d, ^3J = 3.9 Hz, 1H), 7.85 (brs, 1H), 7.56 (d, ^3J = 3.9 Hz, 1H), 7.26 (t, ^3J = 7.5 Hz, 1H), 7.18 (d, ^3J = 7.8 Hz, 1H), 7.11 (brs, 1H), 7.02 (d, ^3J = 3.9 Hz, 1H), 6.82 (d, ^3J = 8.0 Hz, 1H), 6.64-6.62 (m, 1H), 3.69-3.66 (m, 4H), 3.54 (s, 4H), 3.00 (t, ^3J = 7.0 Hz, 2H), 2.43 (t, ^3J = 7.1 Hz, 2H), 1.88 (quin, ^3J = 7.0 Hz, 2H). ^{13}C-NMR: (DMSO-D_6, 100 MHz), δ [ppm] = 193.4 (1C), 171.0 (1C), 159.0 (1C), 158.5 (1C), 151.8 (1C), 147.4 (1C), 145.4 (1C), 142.7 (1C), 134.8 (1C), 134.4 (1C), 131.0 (1C), 125.4 (1C), 117.4 (1C), 116.8 (1C), 116.4 (1C), 113.1 (1C), 111.9 (1C), 45.2 (2C), 41.5 (2C), 38.0 (1C), 32.0 (1C), 20.4 (1C). IR: ν [cm^{-1}] = 3524 (w), 3129 (m), 2884 (w), 1653 (s), 1610 (vs), 1475 (m), 1416 (s), 1338 (w), 1269 (s), 1189 (s), 1071 (w), 934 (m), 856 (m), 737 (vs), 609 (w), 520 (m), 471 (m). MS (ESI+): m/z (%) = 475 (100, $[M+Na]^+$). HRMS (ESI+): calculated for $C_{24}H_{24}NaN_2O_5S$: 475.1298; found: 475.1305.

1-(4-(2-Furoylpiperazine-1-yl)-5-(5-(3-hydroxyphenyl)thiophene-2-yl)pentan-1-one (33b)

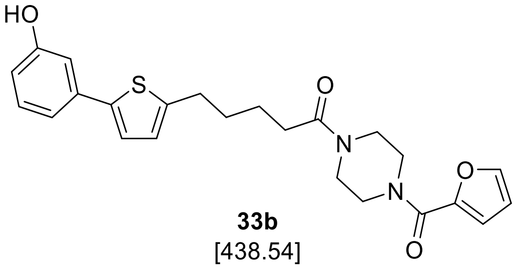

33b
[438.54]

The title compound was synthesised as described in the **general procedure 1A** with **2b** (100 mg, 0.36 mmol, 1.0 eq.), furan-2-yl-piperazinyl-methanone (65.0 mg, 0.36 mmol, 1.0 eq.), EDC•HCl (104 mg, 0.54 mmol, 1.5 eq.), HOBt (73 mg, 0.54 mmol, 1.5 eq.) and NEt_3 (0.15 mL, 1.08 mmol, 3.0 eq.) in DCM (25 mL). The crude product was purified *via* column chromatography (cyclohexane:EtOAc 1:3) and recystallised of EtOAc. 61 mg (0.14 mmol, 39%) of the desired product **33b** as colourless solid was obtained. R_f = 0.15 (cyclohexane:EtOAc 1:3). Melting point: 136 °C. ^{1}H-NMR: (DMSO-D_6, 400 MHz), δ [ppm] = 9.50 (s, 1H), 7.84-7.83 (m, 1H), 7.22 (d, ^3J = 3.4 Hz, 1H), 7.17 (t, ^3J = 7.9 Hz, 1H), 7.02-6.99 (m, 2H), 6.95 (t, ^3J = 3.7 Hz, 1H), 6.83 (d, ^3J = 3.7 Hz, 1H), 6.67 (dd, ^3J = 8.0 Hz, ^4J = 2.3 Hz, 1H), 6.63-6.62 (m, 1H), 3.66 (s, 4H), 3.54-3.51 (m, 4H), 2.81 (t, ^3J = 7.1 Hz, 2H), 2.38 (t, ^3J = 7.2 Hz, 2H), 1.67 (quin, ^3J = 7.3 Hz, 2H), 1.59 (quin, ^3J = 7.0 Hz, 2H). ^{13}C-NMR: (DMSO-D_6, 100 MHz), δ [ppm] = 170.8 (1C), 158.5 (1C), 157.8 (1C), 146.8 (1C), 144.9 (1C), 144.5 (1C), 140.9 (1C), 135.2 (1C), 130.1 (1C), 125.7 (1C), 123.1 (1C), 115.9 (2C), 114.3 (1C), 111.7 (1C), 111.4 (1C), 44.8 (2C), 41.0 (1C), 32.0 (1C), 30.7 (1C), 29.3 (1C), 24.1 (1C). IR: ν [cm^{-1}] = 3125 (w), 3105 (w), 2947 (w), 2915 (w), 2896 (w), 2866 (w), 1614 (vs), 1591 (s), 1573 (s), 1549 (m), 1474 (s), 1427 (vs), 1386 (m), 1369 (m), 1292 (s), 1248 (s), 1239 (s), 1225 (s), 1206 (m), 1188 (s), 1169 (m), 1083 (w), 1051 (w), 1024 (m), 1016 (m), 1007 (s), 985 (m), 938 (m), 911 (w), 883 (w), 857 (s), 801 (m), 777 (vs), 749 (s), 736 (s), 686 (m), 639 (w), 612 (w), 593 (m), 576 (w), 562 (w), 528 (m), 487 (m), 449 (m). MS (ESI+): m/z (%) = 439 (17, $[M+H]^+$), 456 (72, $[M+NH_4]^+$), 461 (10, $[M+Na]^+$). HRMS (ESI+) calculated for $C_{24}H_{27}N_2O_4S$: 439.1686; found: 439.1690 and calculated for $C_{24}H_{26}N_2NaO_4S$: 461.1505; found: 461.1512.

1-(5-(3-Hydroxyphenyl)thiophene-2-yl)-5-(4-(pyrimidine-2-yl)piperazine-1-yl)pentane-1,5-dione (34a)

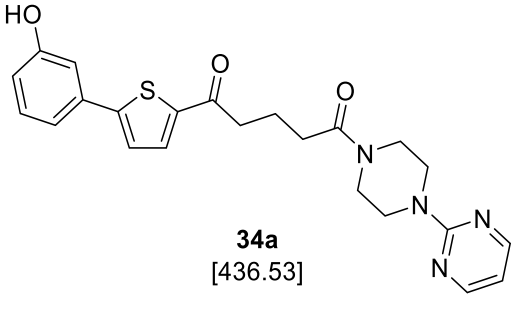

34a
[436.53]

The title compound was synthesised as described in the **general procedure 1A** with **2a** (200 mg, 0.69 mmol, 1.0 eq.), pyrimidine-2-yl-piperazine (113 mg, 0.69 mmol, 1.0 eq.), NEt_3 (0.3 mL, 2.07 mmol, 3.0 eq.), HOBt (140 mg, 1.04 mmol, 1.5 eq.) and EDC•HCl (199 mg, 1.04 mmol, 1.5 eq.) in DCM (50 mL). The crude product was purified *via* column chromatography (DCM:MeOH 10:1). 241 mg (0.55 mmol, 80%) of the desired product **34a** as pale-yellow solid was obtained. $R_f = 0.53$ (DCM:MeOH 10:1). Melting point: 192-195 °C. ^{1}H-NMR: (DMSO-D$_6$, 400 MHz), δ [ppm] = 9.67 (s, 1H), 8.34 (d, ^3J = 4.8 Hz, 2H), 7.88 (d, ^3J = 4.1 Hz, 1H), 7.51 (d, ^3J = 3.9 Hz, 1H), 7.22 (t, ^3J = 7.8 Hz, 1H), 7.15 (d, ^3J = 8.2 Hz, 1H), 7.07 (t, 1H, ^4J = 2.0 Hz), 6.78 (dd, ^3J = 7.6 Hz, ^4J = 3.2 Hz, 1H), 6.62 (t, ^3J = 4.8 Hz, 1H), 3.72-3.68 (m, 4H), 3.51 (s, 4H), 2.98 (t, ^3J = 7.2 Hz, 2H), 2.41 (t, ^3J = 7.3 Hz, 2H), 1.85 (quin, ^3J = 7.2 Hz, 2H). ^{13}C-NMR: (DMSO-D$_6$, 100 MHz), δ [ppm] = 193.4 (1C), 171.0 (1C), 161.6 (1C), 158.5 (3C), 151.8 (1C), 142.7 (1C), 134.8 (1C), 134.4 (1C), 131.0 (1C), 125.4 (1C), 117.4 (1C), 116.8 (1C), 113.1 (1C), 110.9 (1C), 45.0 (1C), 44.0 (1C), 43.6 (1C), 41.3 (1C), 38.0 (1C), 32.1 (1C), 20.4 (1C). IR: ν [cm^{-1}] = 3102 (w), 2934 (w), 1693 (m), 1653 (s), 1623 (s), 1580 (vs), 1545 (s), 1487 (m), 1416 (m), 1362 (m), 1235 (m), 1180 (w), 1126 (w), 1034 (m), 976 (s), 924 (m), 856 (m), 801 (m), 688 (m), 585 (w), 512 (m) 461 (w). MS (ESI+): m/z (%) = 459 (100, [M+Na]$^+$). HRMS (ESI+) calculated for $C_{23}H_{24}NaN_4O_3S$: 459.1461; found: 459.1466.

5-(5-(3-Hydroxyphenyl)thiophene-2-yl)-1-(4-(pyrimidine-2-yl)piperazine-1-yl)pentane-1-one (34b)

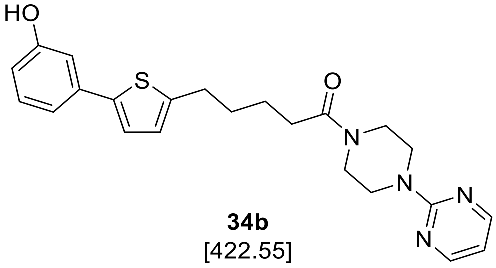

34b
[422.55]

The title compound was synthesised as described in the **general procedure 1A** with **2b** (100 mg, 0.36 mmol, 1.0 eq.), pyrimidine-2-yl-piperazine (0.1 mL, 0.36 mmol, 1.0 eq.), EDC•HCl (104 mg, 0.54 mmol, 1.5 eq.), HOBt (73 mg, 0.54 mmol, 1.5 eq.) and NEt_3 (0.15 mL, 1.08 mmol, 3.0 eq.) in DCM (25 mL). The crude product was purified *via* column chromatography (cyclohexane:EtOAc 2:1). 76 mg (0.18 mmol, 50%) of the desired product **34b** as colourless solid was obtained. $R_f = 0.30$

(cyclohexane:EtOAc 2:1). Melting point: 193 °C. ^{1}H-NMR: (DMSO-D$_6$, 400 MHz), δ [ppm] = 9.50 (s, 1H), 8.37 (d, ^{3}J = 2.3 Hz, 2H), 7.21 (d, ^{3}J = 3.7 Hz, 1H), 7.16 (t, ^{3}J = 7.9 Hz, 1H), 7.00 (d, ^{3}J = 7.8 Hz, 1H), 6.95 (t, ^{3}J = 1.8 Hz, 1H), 6.83 (d, ^{3}J = 3.4 Hz, 1H), 6.68-6.64 (m, 2H), 3.73-3.69 (m, 4H), 3.52 (t, ^{3}J = 5.2 Hz, 4H), 2.82 (t, ^{3}J = 7.1 Hz, 2H), 2.40 (t, ^{3}J = 7.2 Hz, 2H), 1.71-1.56 (m, 4H). ^{13}C-NMR: (DMSO-D$_6$, 100 MHz), δ [ppm] = 170.7 (1C), 161.1 (1C), 158.0 (2C), 157.8 (1C), 144.5 (1C), 140.9 (1C), 135.2 (1C), 130.1 (1C), 125.7 (1C), 123.1 (1C), 115.8 (1C), 114.3 (1C), 111.7 (1C), 110.4 (1C), 44.6 (1C), 43.5 (1C), 43.1 (1C), 40.7 (1C), 32.0 (1C), 30.7 (1C), 29.3 (1C), 24.2 (1C). IR: ν [cm^{-1}] = 3078 (w), 3019 (w), 2947 (w), 2915 (w), 2895 (m), 2865 (m), 1615 (m), 1588 (vs), 1549 (s), 1497 (s), 1475 (m), 1466 (m), 1436 (vs), 1394 (m), 1364 (s), 1308 (m), 1291 (m), 1267 (m), 1241 (s), 1224 (s), 1204 (s), 1078 (w), 1055 (w), 1029 (m), 1014 (m), 982 (s), 957 (m), 912 (m), 862 (m), 855 (m), 800 (m), 791 (m), 775 (s), 730 (m), 682 (m), 638 (m), 623 (m), 490 (m), 438 (m). MS (ESI+): m/z (%) = 423 (100, [M+H]$^+$), 445 (10, [M+Na]$^+$). HRMS (ESI+) calculated for $C_{23}H_{27}N_4O_2S$: 423.1849; found: 423.1854.

1-(4-Acetylpiperazine-1-yl)-5-(5-(3-hydroxyphenyl)thiophene-2-yl)pentane-1,5-dione (35a)

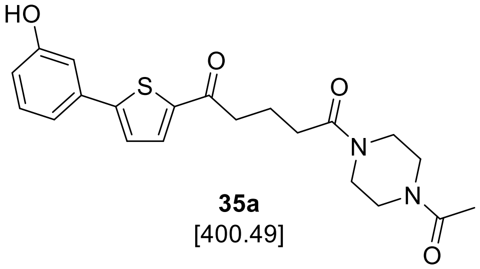

35a
[400.49]

The title compound was synthesised as described in the **general procedure 1A** with **2a** (200 mg, 0.69 mmol, 1.0 eq.), 1-(piperazin-1-yl)ethanone (88.0 mg, 0.69 mmol, 1.0 eq.), NEt_3 (0.3 mL, 2.07 mmol, 3.0 eq.), HOBt (140 mg, 1.04 mmol, 1.5 eq.) and EDC•HCl (199 mg, 1.04 mmol, 1.5 eq.) in DCM (50 mL). The crude product was purified *via* column chromatography (cyclohexane:EtOAc 4:1). 96.5 mg (0.24 mmol, 35%) of the desired product **35a** as colourless solid was obtained. R_f = 0.26 (cyclohexane:EtOAc 4:1). Melting point: 197-201 °C. ^{1}H-NMR: (DMSO-D$_6$, 400 MHz), δ [ppm] = 9.71 (s, 1H), 7.92 (d, ^{3}J = 3.9 Hz, 1H), 7.55 (d, ^{3}J = 4.1 Hz, 1H), 7.26 (t, ^{3}J = 7.9 Hz, 1H), 7.18 (d, ^{3}J = 7.8 Hz, 1H), 7.11 (s, 1H), 6.82 (dd, ^{3}J = 8.0 Hz, ^{4}J = 2.1 Hz, 1H), 3.50-3.40 (m, 8H), 3.00 (t, ^{3}J = 7.1 Hz, 2H), 2.42 (t, ^{3}J = 7.3 Hz, 2H),

2.01 (s, 3H), 1.86 (quin, ^3J = 7.2 Hz, 2H). ^{13}C-NMR: (DMSO-D_6, 100 MHz), δ [ppm] = 192.9 (1C), 170.5 (1C), 168.5 (1C), 158.0 (1C), 151.3 (1C), 142.2 (1C), 134.3 (1C), 133.9 (1C), 130.4 (1C), 124.9 (1C), 116.8 (1C), 116.3 (1C), 112.6 (1C), 45.7 (1C), 44.8 (1C), 41.1 (1C), 40.8 (1C), 37.1 (1C), 31.5 (1C), 21.3 (1C), 19.9 (1C). IR: ν [cm^{-1}] = 3084 (w), 2863 (w), 1682 (w), 1646 (vs), 1574 (m), 1486 (m), 1435 (s), 1337 (m), 1275 (m), 1232 (s), 1170 (m), 934 (w), 885 (m), 836 (s), 789 (s), 695 (s), 601 (m), 550 (w), 471 (m). MS (ESI-): m/z (%) = 399 (100, [M-H]$^-$). HRMS (ESI-) calculated for $C_{21}H_{23}N_2O_4S$: 399.1384; found: 399.1388.

1-(4-Acetylpiperazine-1-yl)-5-(5-(3-hydroxyphenyl)thiophene-2-yl)pentane-1-one (35b)

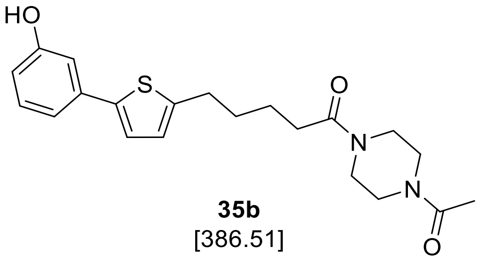

35b
[386.51]

The title compound was synthesised as described in the **general procedure 1A** with **2b** (100 mg, 0.36 mmol, 1.0 eq.), 1-(piperazin-1-yl)ethanone (46.0 mg, 0.36 mmol, 1.0 eq.), EDC•HCl (104 mg, 0.54 mmol, 1.5 eq.), HOBt (73 mg, 0.54 mmol, 1.5 eq.) and NEt_3 (0.15 mL, 1.08 mmol, 3.0 eq.) in DCM (25 mL). The crude product was purified *via* column chromatography (DCM:MeOH 20:1) and recrystallised of EtOAc. 94 mg (0.24 mmol, 67%) of the desired product **35b** as yellow solid was obtained. R_f = 0.15 (DCM:MeOH 20:1). Melting point: 163 °C. ^{1}H-NMR: (DMSO-D_6, 400 MHz), δ [ppm] = 9.67 (s, 1H), 7.21 (d, ^3J = 3.5 Hz, 1H), 7.16 (t, ^3J = 7.9 Hz, 1H), 6.99 (d, ^3J = 7.8 Hz, 1H), 6.95 (brs, 1H), 6.82 (d, ^3J = 3.5 Hz, 1H), 6.68-6.65 (m, 1H), 3.46-3.39 (m, 8H), 2.81 (t, ^3J = 7.2 Hz, 2H), 2.37 (t, ^3J = 7.2 Hz, 2H), 2.00 (s, 3H), 1.70-1.53 (m, 4H). ^{13}C-NMR: (DMSO-D_6, 100 MHz), δ [ppm] = 170.7 (1C), 168.4 (1C), 158.1 (1C), 144.5 (1C), 141.0 (1C), 135.2 (1C), 130.1 (1C), 125.7 (1C), 123.0 (1C), 115.6 (1C), 114.4 (1C), 111.8 (1C), 79.1 (1C), 45.7 (1C), 45.4 (1C), 44.9 (1C), 44.6 (1C), 31.9 (1C), 30.7 (1C), 29.3 (1C), 24.2 (1C), 21.3 (1C). IR: ν [cm^{-1}] = 3139 (w), 2945 (w), 2915 (w), 2898 (w), 2866 (w), 1651 (s), 1618 (s), 1594 (vs), 1551 (w), 1514 (w), 1473 (s), 1438 (vs), 1367 (m), 1295 (m), 1285 (m), 1243 (vs), 1228 (s), 1208 (s), 1174 (w), 1115 (w), 1083 (w), 1051 (w), 1031 (m), 998 (m), 987 (s), 910 (w), 860 (m), 800 (m), 777 (s), 748

(m), 730 (m), 686 (m), 642 (w), 624 (w), 593 (w), 572 (w), 538 (m), 494 (m), 447 (m). MS (ESI+): m/z (%) = 387 (90, $[M+H]^+$), 404 (100, $[M+NH_4]^+$), 409 (60, $[M+Na]^+$). HRMS (ESI+) calculated for $C_{21}H_{27}N_2O_3S$: 387.1737 found: 387.1747 and calculated for: $C_{21}H_{26}N_2NaO_3S$: 409.1556; found: 409.1571.

1-(4-(2-Hydroxyethyl)piperazine-1-yl)-5-(5-(3-hydroxyphenyl)thiophene-2-yl)pentane-1,5-dione (36a)

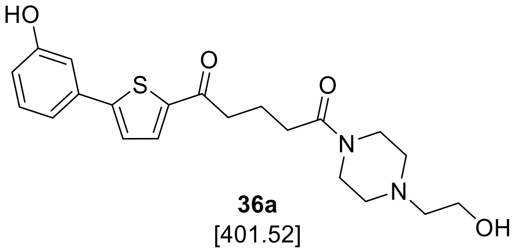

36a
[401.52]

The title compound was synthesised as described in the **general procedure 1A** with **2a** (300 mg, 1.03 mmol, 1.0 eq.), 2-piperazin-1-ylethanol (0.1 mL, 1.03 mmol, 1.0 eq.), NEt_3 (0.43 mL, 3.09 mmol, 3.0 eq.), HOBt (209 mg, 1.55 mmol, 1.5 eq.) and EDC•HCl (297 mg, 1.55 mmol, 1.5 eq.) in DCM (50 mL). The crude product was purified *via* column chromatography (cyclohexane:EtOAc 2:1 → DCM:MeOH 10:1). 245 mg (0.61 mmol, 59%) of the desired product **36a** as colourless solid was obtained. R_f = 0.27 (DCM:MeOH 10:1). Melting point: 234-238 °C. ^{1}H-NMR: (DMSO-D$_6$, 400 MHz), δ [ppm] = 9.79 (s, 1H), 7.91 (d, ^3J = 3.9 Hz, 1H), 7.55 (d, ^3J = 3.9 Hz, 1H), 7.26 (t, ^3J = 7.9 Hz, 1H), 7.18 (d, ^3J = 7.8 Hz, 1H), 7.13 (s, 1H), 6.84-6.82 (m, 1H), 5.33 (s, 1H), 4.43-4.40 (m, 1H), 4.05-4.02 (m, 1H), 3.79 (t, ^3J = 4.6 Hz, 2H), 3.57-3.51 (m, 3H), 3.32 (s, 2H), 3.16-3.08 (m, 3H), 2.99 (t, ^3J = 7.1 Hz, 2H), 2.45-2.42 (m, 2H), 1.86 (quin, ^3J = 7.1 Hz, 2H). ^{13}C-NMR: (DMSO-D$_6$, 100 MHz), δ [ppm] = 192.8 (1C), 170.6 (1C), 158.0 (1C), 151.4 (1C), 142.1 (1C), 134.3 (1C), 133.9 (1C), 130.4 (1C), 124.9 (1C), 116.8 (1C), 116.4 (1C), 112.6 (1C), 57.8 (1C), 55.0 (1C), 51.3 (1C), 51.0 (1C), 41.6 (1C), 37.8 (1C), 37.4 (1C), 31.2 (1C), 19.7 (1C). IR: ṽ [cm^{-1}] = 3200 (m), 2971 (w), 1652 (vs), 1584 (m), 1447 (s), 1308 (w), 1284 (m), 1189 (m), 1071 (w), 1032 (w), 991 (m), 844 (m), 757 (m), 678 (m), 537 (w). MS (ESI+): m/z (%) = 425 (100, $[M+Na]^+$). HRMS (ESI+) calculated for $C_{21}H_{26}NaN_2O_4S$: 425.1505; found: 425.1505.

1-(4-(2-Hydroxyethyl)piperazine-1-yl)-5-(5-(3-hydroxyphenyl)thiophene-2-yl)pentane-1-one (36b)

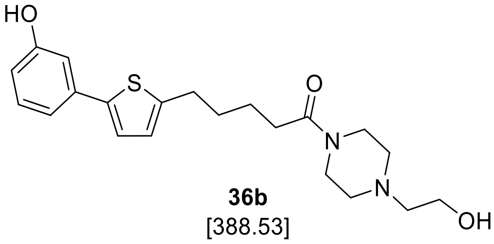

36b
[388.53]

The title compound was synthesised as described in the **general procedure 1A** with **2b** (100 mg, 0.36 mmol, 1.0 eq.), 2-piperazinethanol (0.05 mL, 0.36 mmol, 1.0 eq.), EDC•HCl (104 mg, 0.54 mmol, 1.5 eq.), HOBt (73 mg, 0.54 mmol, 1.5 eq.) and NEt_3 (0.15 mL, 1.08 mmol, 3.0 eq.) in DCM (25 mL). The crude product was purified *via* column chromatography (DCM:MeOH 40:1 → 30:1 → 20:1 → 10:1) and recrystallised of EtOAc. 59 mg (0.15 mmol, 42%) of the desired product **36b** as yellow solid was obtained. R_f = 0.16 (DCM:MeOH 40:1). Melting point: 147 °C. ^{1}H-NMR: (DMSO-D_6, 400 MHz), δ [ppm] = 9.50 (s, 1H), 7.22 (d, ^3J = 3.7 Hz, 1H), 7.17 (t, ^3J = 7.9 Hz, 1H), 7.01 (d, ^3J = 7.8 Hz, 1H), 6.95 (t, ^3J = 2.0 Hz, 1H), 6.82 (d, ^3J = 3.7 Hz, 1H), 6.67 (dd, ^3J = 8.0 Hz, ^4J = 2.3 Hz, 1H), 4.38 (t, ^3J = 5.4 Hz, 1H), 3.49 (q, ^3J = 5.9 Hz, 2H), 3.43-3.39 (m, 4H), 2.80 (t, ^3J = 7.2 Hz, 2H), 2.39-2.31 (m, 8H), 1.65 (quin, ^3J = 7.3 Hz, 2H), 1.55 (quin, ^3J = 7.1 Hz, 2H). ^{13}C-NMR: (DMSO-D_6, 100 MHz), δ [ppm] = 170.4 (1C), 158.0 (1C), 144.5 (1C), 140.9 (1C), 135.2 (1C), 130.1 (1C), 125.7 (1C), 123.1 (1C), 115.9 (1C), 114.3 (1C), 111.7 (1C), 60.1 (1C), 58.5 (1C), 53.5 (1C), 53.0 (1C), 45.0 (1C), 41.0 (1C), 31.9 (1C), 30.7 (1C), 29.3 (1C), 24.3 (1C). IR: ν [cm^{-1}] = 3446 (m), 3076 (w), 3051 (w), 3017 (w), 2949 (m), 2927 (m), 2882 (m), 2865 (m), 2812 (m), 2776 (m), 2745 (m), 2690 (w), 1618 (s), 1593 (vs), 1547 (m), 1516 (w), 1474 (s), 1450 (vs), 1413 (m), 1380 (m), 1364 (m), 1300 (m), 1288 (m), 1273 (m), 1247 (m), 1228 (m), 1273 (m), 1247 (m), 1228 (m), 1204 (s), 1164 (w), 1144 (w), 1128 (m), 1087 (w), 1065 (w), 1051 (m), 1041 (m), 1026 (m), 998 (m), 986 (s), 914 (w), 864 (m), 854 (m), 814 (m), 782 (m), 745 (s), 731 (s), 685 (m), 646 (m), 602 (m), 594 (m), 570 (m), 557 (m), 536 (m), 521 (m), 490 (m), 443 (s), 424 (w), 412 (w). MS (ESI+): m/z (%) = 389 (100, $[M+H]^+$). HRMS (ESI+) calculated for: $C_{21}H_{29}N_2O_3S$: 389.1893; found: 389.1909.

2-(4-(5-(5-(3-Hydroxyphenyl)thiophene-2-yl)-5-oxopentanoyl)piperazine-1-yl)-*N*,*N*-dimethylethan-1-ammonium (2,2,2-trifluoroacetate) (37a)

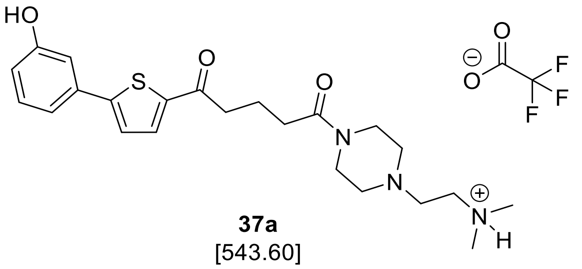

37a
[543.60]

The title compound was synthesised as described in the **general procedure 1A** with **2a** (300 mg, 1.03 mmol, 1.0 eq.), *N*,*N*-dimethyl-2-piperazin-1-ylethanamine (0.1 mL, 1.03 mmol, 1.0 eq.), NEt_3 (0.43 mL, 3.09 mmol, 3.0 eq.), HOBt (209 mg, 1.55 mmol, 1.5 eq.) and EDC•HCl (297 mg, 1.55 mmol, 1.5 eq.) in DCM (25 mL). The crude product was purified *via* column chromatography (DCM:MeOH 20:1 → 10:1). Furthermore, the product was purified *via* HPLC (product signal peak: 30% acetonitrile : 70% H_2O). 129 mg (0.30 mmol, 29%) of the desired product **37a** as colourless solid was obtained. R_f = 0.13 (DCM:MeOH 10:1). Melting point: 142-146 °C. ^{1}H-NMR: (acteone-D_6, 400 MHz), δ [ppm] = 7.86 (d, ^3J = 3.9 Hz, 1H), 7.48 (d, ^3J = 3.9 Hz, 1H), 7.31-7.27 (m, 1H), 7.24-7.22 (m, 2H), 6.91-6.88 (m, 1H), 3.69 (s, 4H), 3.56 (t, ^3J = 6.4 Hz, 2H), 3.25-3.17 (m, 2H), 3.05-3.01 (m, 8H), 2.89-2.83 (m, 4H), 2.48 (t, ^3J = 7.1 Hz, 2H), 2.08-1.95 (m, 2H). ^{13}C-NMR: (DMSO-D_6, 100 MHz), δ [ppm] = 192.9 (1C), 170.4 (1C), 158.6 (q, ^2J = 32.5 Hz, 1C), 158.0 (1C), 151.4 (1C), 142.2 (1C), 134.4 (1C), 133.9 (1C), 130.5 (1C), 124.9 (1C), 116.8 (1C), 116.4 (1C), 115.3 (1C), 112.6 (1C), 52.2 (1C), 51.8 (1C), 50.8 (1C), 49.3 (1C), 49.2 (1C), 45.7 (1C), 42.6 (2C), 37.4 (1C), 31.3 (1C), 19.9 (1C). IR: ν [cm^{-1}] = 3366 (s), 2676 (w), 2460 (w), 1644 (vs), 1447 (vs), 1377 (m), 1278 (m), 1235 (m), 1175 (m), 1103 (m), 988 (m), 865 (w), 786 (m), 744 (s), 598 (w), 511 (w). MS (ESI+): m/z (%) = 452 (100, [M+Na]$^+$). HRMS (ESI+) calculated for $C_{23}H_{31}NaN_3O_3S^+$: 452.1978; found: 452.1977.

(2-(4-(5-(5-(3-Hydroxyphenyl)thiophene-2-yl)pentanoyl)piperazin-1-yl) (2,2,2-trifluoroacetate)-*N*,*N*-dimethylethan-1-ammonium (37b)

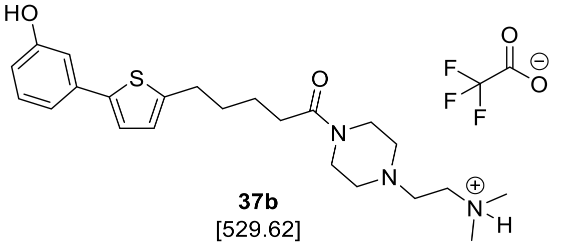

37b
[529.62]

The title compound was synthesised as described in the **general procedure 1A** with **2b** (100 mg, 0.36 mmol, 1.0 eq.), *N*,*N*-dimethyl-2-piperazin-1-ylethanamine (0.04 mL, 0.36 mmol, 1.0 eq.), EDC•HCl (104 mg, 0.54 mmol, 1.5 eq.), HOBt (73 mg, 0.54 mmol, 1.5 eq.) and NEt_3 (0.15 mL, 1.08 mmol, 3.0 eq.) in DCM (25 mL). The crude product was purified *via* column chromatography (DCM:MeOH 10:1 + 1% NEt_3) and recrystallised from EtOAc/MeOH. Furthermore, the product was purified *via* HPLC (product signal peak: 37% MeCN : 63% H_2O). 88 mg (0.17 mmol, 47%) of the desired product **37b** as pale-brown solid was obtained. R_f = 0.28 (DCM:MeOH 10:1). Melting point: 129 °C. ^{1}H-NMR: (DMSO-D_6, 400 MHz), δ [ppm] = 9.64 (brs, 1H), 7.22 (d, ^3J = 3.4 Hz, 1H), 7.17 (t, ^3J = 7.9 Hz, 1H), 7.01 (d, ^3J = 7.8 Hz, 1H), 6.95 (t, ^3J = 3.7 Hz, 1H), 6.82 (d, ^3J = 3.4 Hz, 1H), 6.68 (dd, ^3J = 8.5 Hz, ^4J = 1.9 Hz, 1H), 3.63 (brs, 4H), 3.39 (t, ^3J = 6.4 Hz, 2H), 3.17-3.11 (m, 2H), 2.95 (brs, 4H), 2.82-2.79 (m, 8H), 2.39 (t, ^3J = 7.2 Hz, 2H), 1.67 (quin, ^3J = 7.3 Hz, 2H), 1.57 (quin, ^3J = 7.2 Hz, 2H). ^{13}C-NMR: (DMSO-D_6, 100 MHz), δ [ppm] = 170.7 (1C), 158.4 (q, ^2J = 35.6 Hz, 1C), 157.8 (1C), 144.5 (1C), 140.9 (1C), 135.2 (1C), 130.1 (1C), 125.7 (1C), 1231 (1C), 115.8 (1C), 114.3 (1C), 111.7 (1C), 52.0 (1C), 51.6 (1C), 50.3 (1C), 50.2 (1C), 44.6 (1C), 42.7 (2C), 40.7 (1C), 31.6 (1C), 30.7 (1C), 29.3 (1C), 24.0 (1C). IR: ν [cm^{-1}] = 3251 (w), 2948 (w), 2442 (w), 2333 (w), 1673 (s), 1623 (m), 1595 (m), 1505 (w), 1459 (m), 1445 (m), 1416 (w), 1296 (w), 1276 (w), 1249 (w), 1198 (s), 1172 (vs), 1122 (vs), 1031 (w), 1015 (w), 975 (m), 914 (w), 857 (w), 829 (m), 813 (w), 790 (m), 721 (vs), 690 (w), 654 (w), 596 (w), 520 (w), 497 (w), 468 (w), 445 (m), 413 (w). MS (ESI+): m/z (%) = 416 (100, [M-trifluoracetate]$^+$). HRMS (ESI+) calculated for $C_{23}H_{34}N_3O_2S$: 416.2366; found: 416.2369.

2-(4-(5-(5-(3-Hydroxyphenyl)thiophene-2-yl)-5-oxopentanoyl)piperazine-1-yl)-*N*-isopropylacetamide (38a)

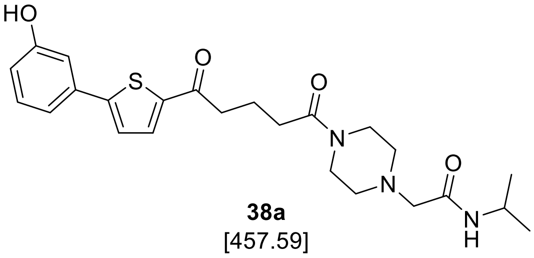

38a
[457.59]

The title compound was synthesised as described in the **general procedure 1A** with **2a** (300 mg, 1.03 mmol, 1.0 eq.), *N*-Isopropyl-2-piperazine-1-yl acetamide (191 mg, 1.03 mmol, 1.0 eq.), NEt_3 (0.43 mL, 3.09 mmol, 3.0 eq.), HOBt (209 mg, 1.55 mmol, 1.5 eq.) and EDC•HCl (297 mg, 1.55 mmol, 1.5 eq.) in DCM (50 mL). The crude product was purified *via* column chromatography (cyclohexane:EtOAc 1:5 → DCM:MeOH 10:1). 454 mg (0.99 mmol, 96%) of the desired product **38a** as colourless solid was obtained. R_f = 0.29 (DCM:MeOH 20:1). Melting point: 120-124 °C. ^{1}H-NMR: (DMSO-D_6, 400 MHz), δ [ppm] = 9.70 (s, 1H), 8.22 (s), 7.91 (d, ^3J = 3.9 Hz, 1H), 7.55 (d, ^3J = 4.1 Hz, 1H), 7.26 (t, ^3J = 7.8 Hz, 1H), 7.19 (d, ^3J = 6.8 Hz, 1H), 7.11 (s, 1H), 6.82 (d, ^3J = 7.8 Hz, 1H), 3.92-3.84 (m, 1H), 3.66-3.58 (m, 1H), 3.49 (s, 2H), 3.17-3.11 (m, 1H), 2.99 (t, ^3J = 7.1 Hz, 2H), 2.73 (s, 1H), 3.39 (t, ^3J = 7.3 Hz, 1H), 1.84 (q, ^3J = 7.2 Hz, 2H), 1.28-1.23 (m, 6H),1.07 (d, ^3J = 6.4 Hz, 6H). ^{13}C-NMR: (DMSO-D_6, 100 MHz), δ [ppm] = 192.9 (1C), 170.2 (2C), 158.0 (1C), 151.3 (1C), 142.2 (1C), 134.3 (1C), 133.9 (1C), 130.4 (1C), 124.9 (1C), 116.9 (1C), 116.3 (1C), 112.6 (1C), 53.6 (1C), 52.8 (1C), 52.4 (1C), 41.8 (1C), 38.0 (1C), 37.5 (1C), 31.5 (1C), 22.3 (1C), 20.0 (1C), 18.1 (1C), 16.7 (1C). IR: ν [cm^{-1}] = 3308 (m), 2969 (m), 1651 (vs), 1524 (w), 1444 (s), 1328 (m), 1278 (m), 1221 (m), 1194 (m), 1151 (m), 1001 (m), 936 (w), 842 (s), 742 (m), 698 (m), 557 (s), 501 (m). MS (ESI-): m/z (%) = 456 (100, [M-H]$^-$). HRMS (ESI-) calculated for $C_{24}H_{30}N_3O_4S$: 456.1963; found: 456.1968.

2-(4-(5-(5-(3-Hydroxyphenyl)thiophene-2-yl)pentanoyl)piperazine-1-yl)-N-isopropylacetamid (38b)

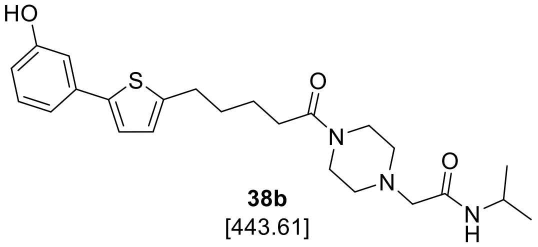

38b
[443.61]

The title compound was synthesised as described in the **general procedure 1A** with **2b** (100 mg, 0.36 mmol, 1.0 eq.), *N*-Isopropyl-2-piperazine-1-yl acetamide (67.0 mg, 0.36 mmol, 1.0 eq.), EDC•HCl (104 mg, 0.54 mmol, 1.5 eq.), HOBt (73 mg, 0.54 mmol, 1.5 eq.) and NEt_3 (0.15 mL, 1.08 mmol, 3.0 eq.) in DCM (25 mL). The crude product was purified *via* column chromatography (DCM:MeOH 30:1) and recrystallised of EtOAc. 118 mg (0.27 mmol, 75%) of the desired product **38b** as yellow solid was obtained. R_f = 0.31 (DCM:MeOH 30:1). Melting point: 142 °C. ^{1}H-NMR: (DMSO-D_6, 400 MHz), δ [ppm] = 9.50 (s, 1H), 7.47 (d, ^3J = 8.0 Hz, 1H), 7.22 (d, ^3J = 3.7 Hz, 1H), 7.17 (t, ^3J = 7.9 Hz, 1H), 7.01 (d, ^3J = 7.6 Hz, 1H), 6.95 (t, ^3J = 1.9 Hz, 1H), 6.82 (d, ^3J = 3.7 Hz, 1H), 6.67 (dd, ^3J = 8.0 Hz, ^4J = 1.4 Hz, 1H), 3.92-3.83 (m, 1H), 3.45 (s, 4H), 2.89 (s, 2H), 2.80 (t, ^3J = 7.3 Hz, 2H), 2.40-2.31 (m, 6H), 1.65 (quin, ^3J = 7.2 Hz, 2H), 1.55 (quin, ^3J = 7.3 Hz, 2H), 1.06 (d, ^3J = 6.6 Hz, 6H). ^{13}C-NMR: (DMSO-D_6, 100 MHz), δ [ppm] = 170.4 (1C), 167.8 (1C), 158.0 (1C), 144.5 (1C), 140.8 (1C), 135.1 (1C), 130.0 (1C), 125.6 (1C), 123.0 (1C), 115.8 (1C), 114.3 (1C), 111.7 (1C), 60.9 (1C), 52.9 (1C), 52.5 (1C), 44.8 (1C), 40.9 (1C), 40.0 (1C), 31.9 (1C), 30.6 (1C), 29.2 (1C), 24.2 (1C), 22.3 (2C). IR: ν [cm^{-1}] = 3380 (w), 3048 (w), 2968 (w), 2943 (m), 2913 (m), 2897 (m), 2865 (m), 2825 (m), 1676 (vs), 1615 (m), 1593 (s), 1549 (m), 1510 (s), 1465 (m), 1446 (vs), 1432 (s), 1414 (m), 1395 (m), 1376 (m), 1360 (m), 1334 (m), 1295 (m), 1283 (m), 1264 (m), 1239 (m), 1228 (s), 1206 (s), 1172 (m), 1139 (s), 1129 (m), 1080 (m), 1057 (m), 1039 (m), 1000 (m), 987 (m), 965 (m), 922 (w), 909 (m), 863 (m), 822 (m), 800 (m), 778 (s), 754 (s), 686 (m), 664 (w), 636 (w), 624 (w), 603 (m), 561 (m), 518 (m), 496 (m), 484 (w), 445 (m). MS (ESI+): m/z (%) = 344 (100, $[M+H]^+$), 466 (10, $[M+Na]^+$). HRMS (ESI+) calculated for $C_{24}H_{33}N_3O_3S$: 444.2351; found: 444.2326.

1-(4-Ethylpiperazine-1-yl)-5-(5-(3-hydroxyphenyl)thiophene-2-yl)pentane-1,5-dione (39a)

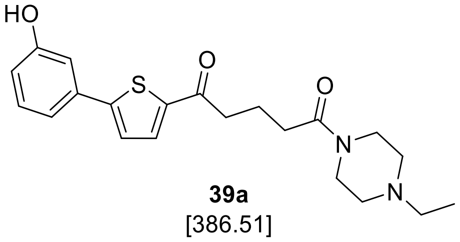

39a
[386.51]

The title compound was synthesised as described in the **general procedure 1A** with **2a** (300 mg, 1.03 mmol, 1.0 eq.), 1-ethylpiperazine (0.1 mL, 1.03 mmol, 1.0 eq.), NEt_3 (0.43 mL, 3.09 mmol, 3.0 eq.), HOBt (209 mg, 1.55 mmol, 1.5 eq.) and EDC•HCl (297 mg, 1.55 mmol, 1.5 eq.) in DCM (50 mL). The crude product was purified *via* column chromatography (DCM:MeOH 30:1 → 10:1). 253 mg (0.65 mmol, 63%) of the desired product **39a** as pale-yellow solid was obtained. R_f = 0.22 (DCM:MeOH 20:1). Melting point: 200 °C (under decomposition). ^{1}H-NMR: (DMSO-D$_6$, 400 MHz), δ [ppm] = 9.74 (s, 1H), 7.91 (d, ^{3}J = 3.9 Hz, 1H), 7.55 (d, ^{3}J = 3.9 Hz, 1H), 7.26 (t, ^{3}J = 7.8 Hz, 1H), 7.18 (d, ^{3}J = 7.8 Hz, 1H), 7.12 (s, 1H), 6.83-6.81 (m, 1H), 3.55-3.31 (m, 5H), 2.99 (t, ^{3}J = 7.1 Hz, 2H), 2.67 (s, 4H), 2.42-2.39 (m, 3H), 1.85 (quin, ^{3}J = 7.2 Hz, 2H), 1.12-1.09 (m, 3H). ^{13}C-NMR: (DMSO-D$_6$, 100 MHz), δ [ppm] = 192.8 (1C), 170.3 (1C), 158.0 (1C), 151.3 (1C), 142.2 (1C), 134.3 (1C), 133.9 (1C), 130.4 (1C), 124.9 (1C), 116.8 (1C), 116.3 (1C), 112.6 (1C), 51.8 (1C), 51.5 (1C), 51.1 (2C), 43.5 (1C), 37.4 (1C), 31.4 (1C), 19.9 (1C), 10.6 (1C). IR: ν [cm^{-1}] = 3139 (m), 2962 (m), 2676 (w), 1652 (vs), 1583 (m), 1524 (w), 1447 (s), 1318 (w), 1283 (m), 1193 (m), 1071 (w), 1004 (m), 845 (s), 752 (m), 688 (m), 558 (m), 471 (w). MS (ESI-): m/z (%) = 385 (100, [M-H]$^-$). HRMS (ESI-) calculated for $C_{21}H_{25}N_2O_3S$: 385.1591; found: 385.1592.

1-(4-Ethylpiperazine-1-yl)-5-(5-(3-hydroxyphenyl)thiophene-2-yl)pentane-1-one (39b)

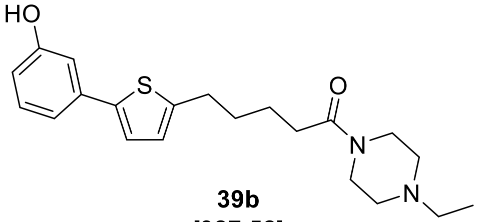

39b
[327.52]

The title compound was synthesised as described in the **general procedure 1A** with **2b** (100 mg, 0.36 mmol, 1.0 eq.), ethylpiperazine (0.05 mL, 0.36 mmol, 1.0 eq.), EDC•HCl (104 mg, 0.54 mmol, 1.5 eq.), HOBt (73 mg, 0.54 mmol, 1.5 eq.) and NEt_3 (0.15 mL, 1.08 mmol, 3.0 eq.) in DCM (25 mL). The crude product was purified *via* column chromatography (DCM:MeOH 20:1) and recrystallised of EtOAc. 87 mg (0.27 mmol, 75%) of the desired product **39b** as yellow solid was obtained. R_f = 0.12 (DCM:MeOH 20:1). Melting point: 124 °C. ^{1}H-NMR: (DMSO-D_6, 400 MHz), δ [ppm] = 9.51 (s, 1H), 7.22 (d, ^3J = 3.4 Hz, 1H), 7.17 (t, ^3J = 7.9 Hz, 1H), 7.00 (d, ^3J = 8.5 Hz, 1H), 6.95 (t, ^3J = 1.9 Hz, 1H), 6.82 (d, ^3J = 3.4 Hz, 1H), 6.67 (ddd, ^3J = 7.8 Hz, ^4J = 2.3 Hz, ^4J = 0.7 Hz, 1H), 3.43-3.39 (m, 4H), 2.80 (t, ^3J = 7.2 Hz, 2H), 2.34-2.25 (m, 8H), 1.65 (quin, ^3J = 7.2 Hz, 2H), 1.55 (quin, ^3J = 7.3 Hz, 2H), 0.98 (t, ^3J = 7.2 Hz, 3H). ^{13}C-NMR: (DMSO-D_6, 100 MHz), δ [ppm] = 170.4 (1C), 157.8 (1C), 144.5 (1C), 140.9 (1C), 135.2 (1C), 130.1 (1C), 125.7 (1C), 123.1 (1C), 115.4 (1C), 114.3 (1C), 111.6 (1C), 52.7 (1C), 52.2 (1C), 51.5 (1C), 44.9 (1C), 41.0 (1C), 32.0 (1C), 30.7 (1C), 29.3 (1C), 24.3 (1C),12.9 (1C). IR: v [cm^{-1}] = 3169 (m), 3011 (w), 2969 (w), 2951 (w), 2926 (w), 2914 (m), 2836 (w), 2803 (w), 2759 (w), 1621 (vs), 1592 (vs), 1546 (w), 1515 (w), 1472 (s), 1449 (vs), 1415 (m), 1376 (m), 1342 (m), 1302 (m), 1291 (m), 1272 (m), 1243 (s), 1229 (s), 1205 (s), 1163 (m), 1148 (m), 1123 (m), 1090 (w), 1051 (w), 1020 (m), 987 (m), 965 (w), 916 (m), 862 (m), 813 (s), 783 (s), 760 (m), 730 (m), 713 (s), 683 (s), 640 (m), 624 (m), 594 (m), 562 (w), 509 (m), 494 (m), 442 (m). MS (ESI+): m/z (%) = 373 (100, $[M+H]^+$), 395 (5, $[M+Na]^+$). HRMS (ESI+) calculated for $C_{21}H_{29}N_2O_2S$: 373.1944; found: 373.1960.

1-(5-(3-Hydroxyphenyl)thiophene-2-yl)-5-(4-(4-methylbenzyl)piperazine-1-yl)pentane-1,5-dion (40a)

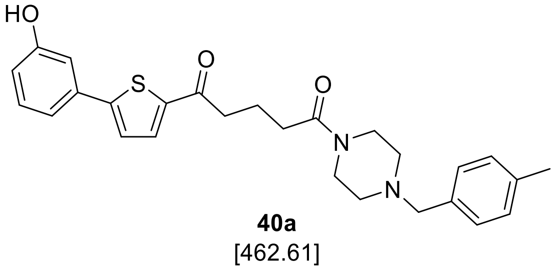

40a
[462.61]

The title compound was synthesised as described in the **general procedure 1A** with **2a** (300 mg, 1.03 mmol, 1.0 eq.), 1-(4-methylbenzyl)piperazine (196 mg, 1.03 mmol, 1.0 eq.), NEt_3 (0.43 mL, 3.09 mmol, 3.0 eq.), HOBt (209 mg, 1.55 mmol, 1.5 eq.) and

EDC•HCl (297 mg, 1.55 mmol, 1.5 eq.) in DCM (50 mL). The crude product was purified *via* column chromatography (DCM:MeOH 20:1). 397 mg (0.86 mmol, 83%) of the desired product **40a** as yellow solid was obtained. R_f = 0.32 (DCM:MeOH 20:1). Melting point: 170-171 °C. ^{1}H-NMR: (DMSO-D_6, 400 MHz), δ [ppm] = 9.71 (s, 1H), 7.91 (d, ^3J = 3.9 Hz, 1H), 7.55 (d, ^3J = 3.9 Hz, 1H), 7.26 (t, ^3J = 7.8 Hz, 1H), 7.19-7.11 (m, 6H), 6.82 (d, ^3J = 6.2 Hz, 1H), 3.43-3.41 (m, 6H), 2.97 (t, ^3J = 7.1 Hz, 2H), 2.36 (t, ^3J = 7.3 Hz, 2H), 2.31-2.27 (m, 7H), 1.85 (quin, ^3J = 6.9 Hz, 2H). ^{13}C-NMR: (DMSO-D_6, 100 MHz), δ [ppm] = 192.8 (1C), 170.0 (1C), 157.9 (1C), 151.3 (1C), 142.2 (1C), 136.0 (1C), 134.7 (1C), 134.2 (1C), 133.9 (1C), 130.4 (1C), 128.8 (2C), 128.7 (2C), 124.8 (1C), 116.8 (1C), 116.3 (1C), 112.5 (1C), 61.6 (1C), 52.7 (1C), 52.2 (1C), 44.8 (1C), 41.0 (1C), 37.4 (1C), 31.4 (1C), 20.6 (1C), 19.9 (1C). IR: ν [cm^{-1}] = 3218 (m), 2939 (m), 2825 (m), 1647 (vs), 1583 (s), 1445 (vs), 1337 (w), 1278 (m), 1224 (s), 1189 (m), 1032 (m), 997 (s), 924 (m), 846 (m), 785 (s), 747 (m), 687 (m), 551 (w), 471 (m). MS (ESI-): m/z (%) = 461 (100, [M-H]$^-$). HRMS (ESI-) calculated for $C_{27}H_{29}N_2O_3S$: 461.1904; found: 461.1905.

5-(5-(3-Hydroxyphenyl)thiophene-2-yl)-1-(4-(4-methylbenzyl)piperazine-1-yl)pentane-1-one (40b)

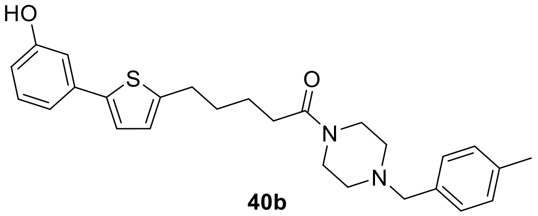

40b
[448.63]

The title compound was synthesised as described in the **general procedure 1A** with **2b** (100 mg, 0.36 mmol, 1.0 eq.), 1-(4-methylbenzyl)piperazine (69.0 mg, 0.36 mmol, 1.0 eq.), EDC•HCl (104 mg, 0.54 mmol, 1.5 eq.), HOBt (73 mg, 0.54 mmol, 1.5 eq.) and NEt_3 (0.15 mL, 1.08 mmol, 3.0 eq.) in DCM (25 mL). The crude product was purified *via* column chromatography (DCM:MeOH 20:1) and recrystallised from EtOAc. 55 mg (0.12 mmol, 33%) of the desired product **40b** as yellow solid was obtained. R_f = 0.11 (DCM:MeOH 20:1). Melting point: 136 °C. ^{1}H-NMR: (DMSO-D_6, 400 MHz), δ [ppm] = 9.50 (s, 1H), 7.22 (d, ^3J = 3.7 Hz, 1H), 7.19-7.10 (m, 5H), 7.01 (d, ^3J = 7.6 Hz, 1H), 6.95 (t, ^3J = 1.9 Hz, 1H), 6.81 (d, ^3J = 3.7 Hz, 1H), 6.67 (ddd, ^3J = 8.0 Hz, ^4J = 2.3 Hz, ^4J = 0.9 Hz, 1H), 3.43-3.40 (m, 6H), 2.79 (t, ^3J = 7.2 Hz, 2H), 2.33-2.26

(m, 9H), 1.64 (quin, ^3J = 7.3 Hz, 2H), 1.55 (quin, ^3J = 7.2 Hz, 2H). ^{13}C-NMR: (DMSO-D$_6$, 100 MHz), δ [ppm] = 170.4 (1C), 157.8 (1C), 144.5 (1C), 140.9 (1C), 136.1 (1C), 135.2 (1C), 134.7 (1C), 130.1 (1C), 128.9 (2C), 128.8 (2C), 125.7 (1C), 123.1 (1C), 115.8 (1C), 114.3 (1C), 111.7 (1C), 61.6 (1C), 52.8 (1C), 52.3 (1C), 44.9 (1C), 41.0 (1C), 32.0 (1C), 30.7 (1C), 29.3 (1C), 24.3 (1C), 20.7 (1C).IR: ν [cm^{-1}] = 3146 (m), 2945 (m), 2930 (m), 2913 (w), 2825 (w), 1616 (s), 1594 (vs), 1550 (m), 1514 (m), 1476 (s), 1451 (vs), 1410 (m), 1361 (w), 1342 (w), 1321 (w), 1295 (m), 1274 (w), 1240 (m), 1228 (s), 1215 (m), 1201 (m), 1162 (w), 1143 (m), 1113 (m), 1102 (m), 1081 (w), 1027 (m), 994 (m), 863 (m), 817 (m), 800 (m), 778 (vs), 744 (s), 686 (s), 639 (w), 574 (w), 552 (m), 514 (m), 487 (m), 466 (w), 443 (m), 429 (m). MS (ESI+): m/z (%) = 449 (100, [M+H]$^+$). HRMS (ESI+) calculated for $C_{27}H_{33}N_2O_2S$: 449.2257; found: 449.2281.

***tert*-Butyl 4-(5-(5-(3-hydroxyphenyl)thiophene-2-yl)-5-oxopentanoyl)piperazine-1-carboxylate (41a)**

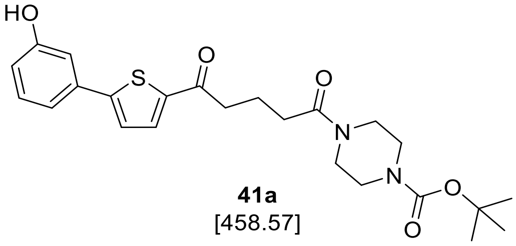

The title compound was synthesised as described in the **general procedure 1A** with **2a** (700 mg, 2.40 mmol, 1.0 eq.), 1-Boc-Piperazin (449 mg, 2.40 mmol, 1.0 eq.), NEt_3 (1.0 mL, 7.20 mmol, 3.0 eq.), HOBt (486 mg, 3.60 mmol, 1.5 eq.) and EDC•HCl (690 mg, 3.60 mmol, 1.5 eq.) in DCM (50 mL). The crude product was purified *via* column chromatography (DCM:MeOH 20:1). 413 mg (0.90 mmol, 38%) of the desired product **41a** as colourless solid was obtained. R_f = 0.21 (cyclohexane:EtOAc 1:5). Melting point: 194-195 °C. ^{1}H-NMR: (DMSO-D$_6$, 400 MHz), δ [ppm] = 9.70 (s, 1H), 7.91 (d, ^3J = 4.1 Hz, 1H), 7.55 (d, ^3J = 3.9 Hz, 1H), 7.27 (t, ^3J = 7.8 Hz, 1H), 7.18 (d, ^3J = 7.8 Hz, 1H), 7.11 (s, 1H), 6.81 (dd, ^3J = 8.0 Hz, ^4J = 2.3 Hz, 1H), 3.43 (s, 4H), 3.33-3.28 (m, 4H), 2.99 (t, ^3J = 7.2 Hz, 2H,), 2.40 (t, ^3J = 7.3 Hz, 2H), 1.85 (quin, ^3J = 7.2 Hz, 2H), 1.40 (s, 9H). ^{13}C-NMR: (DMSO-D$_6$, 100 MHz), δ [ppm] = 192.8 (1C), 170.4 (1C), 157.9 (1C), 153.8 (1C), 151.3 (1C), 142.2 (1C), 134.2 (1C), 133.8 (1C), 130.4 (1C), 124.8 (1C), 116.8 (1C), 116.3 (1C), 112.5 (1C), 79.1 (1C), 44.5 (2C), 40.7 (2C), 37.4 (1C), 31.5 (1C), 28.0 (3C), 19.9 (1C). IR: ν [cm^{-1}] = 3148 (m), 2975 (m),

2884 (m), 1702 (s), 1649 (vs), 1587 (s), 1446 (vs), 1416 (s), 1268 (s), 1229 (s), 1163 (s), 1033 (m), 995 (s), 934 (m), 875 (m), 846 (s), 743 (s), 687 (m), 568 (m), 481 (m). MS (ESI+): m/z (%) = 481 (80, $[M+Na]^+$). HRMS (ESI+) calculated for $C_{24}H_{30}NaN_2O_5S$: 481.1768; found: 481.1769.

tert-Butyl 4-(5-(5-(3-hydroxyphenyl)thiophene-2-yl)pentanoyl)piperazine-1-carboxylate (41b)

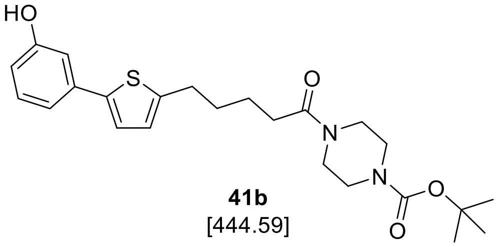

41b
[444.59]

The title compound was synthesised as described in the **general procedure 1A** with **2b** (200 mg, 0.72 mmol, 1.0 eq.), Boc-piperazine (134 mg, 0.72 mmol, 1.0 eq.), EDC•HCl (208 mg, 1.08 mmol, 1.5 eq.), HOBt (146 mg, 1.08 mmol, 1.5 eq.) and NEt_3 (0.3 mL, 2.16 mmol, 3.0 eq.) in DCM (50 mL). The crude product was purified *via* column chromatography (cyclohexane:EtOAc 1:1) and recrystallised of EtOAc. 235 mg (0.53 mmol, 74%) of the desired product **41b** as colourless solid was obtained. R_f = 0.33 (cyclohexane:EtOAc 1:1). Melting point: 155 °C. ^{1}H-NMR: (DMSO-D_6, 400 MHz), δ [ppm] = 9.51 (s, 1H), 7.21 (d, ^3J = 3.7 Hz, 1H), 7.17 (t, ^3J = 7.9 Hz, 1H), 7.00 (d, ^3J = 7.8 Hz, 1H), 6.95 (t, ^3J = 3.9 Hz, 1H), 6.82 (d, ^3J = 3.7 Hz, 1H), 6.67 (dd, ^3J = 8.0 Hz, ^4J = 2.3 Hz, 1H), 3.43-3.40 (m, 4H), 3.33-3.25 (m, 4H), 2.80 (t, ^3J = 7.2 Hz, 2H), 2.35 (t, ^3J = 7.2 Hz, 2H), 1.65 (quin, ^3J = 7.3 Hz, 2H), 1.56 (quin, ^3J = 7.0 Hz, 2H), 1.40 (s, 9H). ^{13}C-NMR: (DMSO-D_6, 100 MHz), δ [ppm] = 170.7 (1C), 157.8 (1C), 153.8 (1C), 144.5 (1C), 140.9 (1C), 135.2 (1C), 130.1 (1C), 125.7 (1C), 123.1 (1C), 115.8 (1C), 114.3 (1C), 111.7 (1C), 79.1 (1C), 44.6 (1C), 42.5 (1C), 40.9 (1C), 40.8 (1C), 32.0 (1C), 30.7 (1C), 29.3 (1C), 28.1 (3C), 24.2 (1C). IR: ν [cm^{-1}] = 3149 (w), 3009 (w), 2976 (w), 2946 (m), 2866 (w), 1690 (s), 1615 (s), 1593 (vs), 1548 (w), 1514 (w), 1474 (m), 1444 (s), 1427 (s), 1410 (s), 1364 (s), 1286 (m), 1243 (vs), 1226 (vs), 1210 (m), 1165 (vs), 1134 (m), 1082 (w), 1054 (w), 1028 (m), 1014 (w), 992 (s), 968 (w), 910 (w), 857 (s), 804 (m), 778 (vs), 763 (s), 730 (m), 685 8m), 650 (w), 622 (w), 544 (w), 493 (m), 447 (m). MS (ESI+): m/z (%) = 445 (50, $[M+H]^+$), 462 (32, $[M+NH_4]^+$), 467 (10,

[M+Na]+). HRMS (ESI+) calculated for $C_{24}H_{33}N_2O_4S$: 445.2156; found: 445.2151 and calculated for $C_{24}H_{32}N_2NaO_4S$: 467.1975; found: 467.1974.

4-(5-(5-(3-Hydroxyphenyl)thiophene-2-yl)-5-oxopentanoyl)piperazinium-chloride (42a)

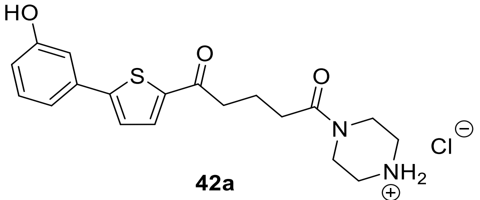

42a
[394.91]

The title compound was synthesised as described in the **general procedure 3** with **41a** (257 mg, 0.56 mmol, 1.0 eq.) and 4 M HCl in 1,4-dioxan (0.6 mL) in DCM (3.0 mL) and MeOH (0.5 mL). 173 mg (0.44 mmol, 79%) of the desired product **42a** as colourless solid was obtained. Melting point: 227 °C (under decomposition). ^{1}H-NMR: (DMSO-D$_6$, 400 MHz), δ [ppm] = 9.78 (s, 1H), 9.42 (s, 2H), 7.91 (d, ^3J = 3.9 Hz, 1H), 7.55 (d, ^3J = 4.1 Hz, 1H), 7.26 (t, ^3J = 7.9 Hz, 1H), 7.18 (d, ^3J = 7.8 Hz, 1H), 7.13 (s, 1H), 6.84-6.82 (m, 1H), 3.70-3.67 (m, 4H), 3.10-2.98 (m, 6H), 2.43 (t, ^3J = 7.2 Hz, 2H), 1.86 (quin, ^3J = 7.2 Hz, 2H). ^{13}C-NMR: (DMSO-D$_6$, 100 MHz), δ [ppm] = 192.8 (1C), 170.6 (1C), 158.0 (1C), 151.4 (1C), 142.2 (1C), 134.3 (1C), 133.9 (1C), 130.4 (1C), 124.9 (1C), 116.8 (1C), 116.4 (1C), 112.6 (1C), 42.7 (1C), 42.5 (1C), 41.7 (1C), 37.8 (1C), 37.4 (1C), 31.2 (1C), 19.7 (1C). IR: ν [cm^{-1}] = 3189 (m), 2972 (m), 2785 (m), 2460 (m), 1656 (vs), 1584 (s), 1545 (w), 1446 (vs), 1279 (s), 1200 (m), 1140 (m), 1027 (m), 934 (w), 874 (w), 845 (m), 775 (s), 688 (m), 550 (w), 494 (m), 452 (w). MS (ESI+): m/z (%) = 359 (80, [M-Cl$^-$]$^+$). HRMS (ESI+) calculated for $C_{19}H_{23}N_2O_3S$: 359.1424; found: 359.1425.

4-(5-(5-(3-Hydroxyphenyl)thiophene-2-yl)-5-pentanoyl)piperazinium-chloride (42b)

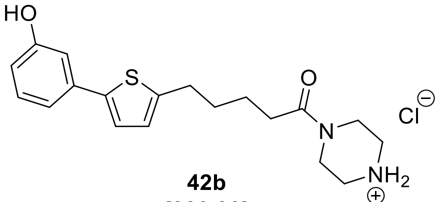

42b
[380.93]

The title compound was synthesised as described in the **general procedure 3** with **41b** (120 mg, 0.27 mmol, 1.0 eq.) and 4M HCl in 1,4-dioxan (2.0 mL) and in DCM (1.5 mL). 16 mg (0.04 mmol, 16%) of the desired product **42b** as colourless solid was obtained. Melting pint: 196 °C. ^{1}H-NMR: (DMSO-D$_6$, 400 MHz), δ [ppm] = 9.55 (s, 1H), 9.26 (s, 2H), 7.22 (d, ^{3}J = 3.5 Hz, 1H), 7.17 (t, ^{3}J = 7.9 Hz, 1H), 7.00 (d, ^{3}J = 7.9 Hz, 1H), 6.96 (brs, 1H), 6.82 (d, ^{3}J = 3.5 Hz, 1H), 6.68 (dd, ^{3}J = 8.1 Hz, ^{4}J = 1.6 Hz, 1H), 3.66 (s, 4H), 3.05 (d, ^{2}J = 21.1 Hz, 4H), 2.81 (t, ^{3}J = 7.2 Hz, 2H), 2.39 (t, ^{3}J = 7.2 Hz, 2H), 1.70-1.53 (m, 4H). ^{13}C-NMR: (DMSO-D$_6$, 100 MHz), δ [ppm] = 170.8 (1C), 157.8 (1C), 144.5 (1C), 140.9 (1C), 135.2 (1C), 130.1 (1C), 125.7 (1C), 123.1 (1C), 115.8 (1C), 114.3 (1C), 111.7 (1C), 42.8 (1C), 42.6 (1C), 41.8 (1C), 37.8 (1C), 31.6 (1C), 30.7 (1C), 29.3 (1C), 23.9 (1C). IR: ν [cm^{-1}] = 3365 (m), 3102 (m), 3009 (m), 2946 (m), 2866 (m), 2803 (m), 2451 (m), 1616 (s), 1593 (vs), 1552 (m) 1513 (w), 1477 (m), 1454 (vs), 1413 (m), 1370 (m), 1296 (m), 1261 (m), 1244 (m), 1227 (s), 1196 (m), 1166 (m), 1145 (m), 1075 (m), 1040 (m), 1024 (m), 1011 (m), 987 (m), 908 (m), 888 (w), 864 (m), 858 (m), 800 (m), 778 (s), 747 (m), 691 (m), 555 (m), 507 (w), 494 (m), 448 (m), 422 (m). MS (ESI+): m/z (%) = 445 (100, [M-Cl$^-$]$^+$). HRMS (ESI+) calculated for $C_{19}H_{25}N_2O_2S$: 345.1631; found: 345.1635.

tert-Butyl 4-(*N,N*-dimethylsulfamoyl)piperazine-1-carboxylate (49)

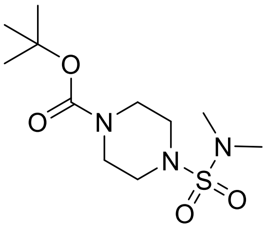

49
[293.38]

The title compound was synthesised as described in the **general procedure 4** with Boc-piperazine (400 mg, 2.15 mmol, 1.0 eq.), *N*,*N*-dimethylsulfamoylchloride (0.2 mL, 2.15 mmol, 1.0 eq.) und NEt_3 (0.9 mL, 6.45 mmol, 3.0 eq.) in 1,4-dioxan (4.5 mL) und H_2O (1.5 mL). The crude product was used without any purification on the next step. 614 mg (2.09 mmol, 97%) of the desired product **49** as colourless solid was obtained. Melting point: 124-127 °C. ^{1}H-NMR: (DMSO-D$_6$, 400 MHz), δ [ppm] = 3.37 (t, ^{3}J = 4.8 Hz, 4H), 3.12 (t, ^{3}J = 5.0 Hz, 4H), 2.76 (s, 6H), 1.41 (s, 9H).^{13}C-NMR: (DMSO-D$_6$, 100 MHz), δ [ppm] = 153.6 (1C), 79.2 (1C), 45.8 (4C), 37.8 (2C), 28.0 (3C). MS

(ESI+): m/z (%) = 316 (100, [M+Na]$^+$). HRMS (ESI+) calculated for $C_{11}H_{23}NaN_3O_4S$: 316.1301; found: 316.1298.

4-(*N,N*-Dimethylsulfamoyl)piperazinium chloride (50)

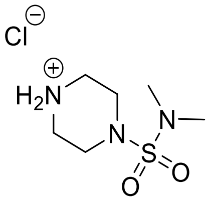

50
[229.72]

The title compound was synthesised as described in the **general procedure 3** with **49** (474 mg, 1.62 mmol, 1.0 eq.) and 4 M HCl in 1,4-dioxan (1.5 mL) in 1,4-dioxan (7 mL). 190 mg (0.83 mmol, 61%) of the desired product **50** as colourless solid was obtained. Melting point: 219-222 °C. ^{1}H-NMR: (DMSO-D$_6$, 400 MHz), δ [ppm] = 9.52 (s, 2H), 3.39 (t, ^3J = 5.2 Hz, 4H), 3.13 (t, ^3J = 5.2 Hz, 4H), 2.78 (s, 6H). ^{13}C-NMR: (DMSO-D$_6$, 100 MHz), δ [ppm] = 42.8 (2C), 42.3 (2C), 37.8 (2C). MS (ESI+): m/z (%) = 194 (100, [M-Cl]$^+$). HRMS (ESI+) calculated for $C_6H_{15}N_3O_2S$: 194.0958; found: 194.0955.

4-(5-(5-(3-Hydroxyphenyl)thiophene-2-yl)-5-oxopentanoyl)-*N*,*N*-dimethylpiperazine-1-sulfonamide (43a)

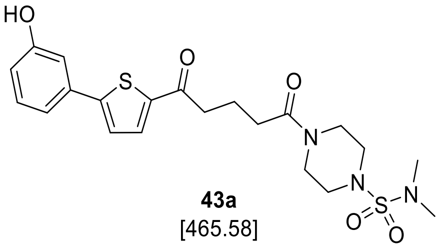

43a
[465.58]

The title compound was synthesised as described in the **general procedure 1A** with **2a** (200 mg, 0.69 mmol, 1.0 eq.), **50** (159 mg, 0.69 mmol, 1.0 eq.), NEt_3 (0.3 mL, 2.07 mmol, 3.0 eq.), HOBt (141 mg, 1.04 mmol, 1.5 eq.) and EDC•HCl (198 mg, 1.04 mmol, 1.5 eq.) in DCM (25 mL). The crude product was purified *via* column chromatography (DCM:MeOH 60:1). 190 mg (0.41 mmol, 59%) of the desired product **43a** as pale-yellow solid was obtained. R_f = 0.33 (DCM:MeOH 60:1). Melting point: 183-185 °C. ^{1}H-NMR: (DMSO-D$_6$, 400 MHz), δ [ppm] = 9.74 (s, 1H), 7.91 (d,

^{3}J = 5.0 Hz, 1H), 7.55-7.54 (m, 1H), 7.26 (t, ^{3}J = 7.8 Hz, 1H), 7.18 (d, ^{3}J = 7.6 Hz, 1H), 7.11 (s, 1H), 6.81 (d, ^{3}J = 6.9 Hz, 1H), 3.50 (s, 4H), 3.16-3.12 (m, 4H), 2.99 (t, ^{3}J = 7.0 Hz, 2H), 2.77 (s, 6H), 2.41 (t, ^{3}J = 7.1 Hz, 2H), 1.85 (quin, ^{3}J = 7.0 Hz, 2H). ^{13}C-NMR: (DMSO-D$_6$, 100 MHz), δ [ppm] = 192.9 (1C), 170.4 (1C), 158.0 (1C), 151.3 (1C), 142.2 (1C), 134.4 (1C), 133.9 (1C), 130.5 (1C), 124.9 (1C), 116.9 (1C), 116.3 (1C), 112.6 (1C), 46.3 (1C), 46.0 (1C), 44.6 (1C), 40.7 (1C), 37.9 (2C), 37.4 (1C), 31.5 (1C), 19.8 (1C). IR: ν [cm^{-1}] = 3184 (m), 2923 (m), 1765 (w), 1645 (vs), 1579 (m), 1534 (w), 1444 (s), 1376 (m), 1342 (m), 1280 (m), 1191 (m), 1142 (s), 1025 (m), 951 (m), 872 (m), 808 (m), 744 (s), 687 (m), 637 (m), 585 (m), 573 (m), 518 (s). MS (ESI-): m/z (%) = 464 (100, [M-H]$^-$). HRMS (ESI-) calculated for $C_{21}H_{26}N_3O_5S_2$: 464.1307; found: 464.1307.

4-(5-(5-(3-Hydroxyphenyl)thiophene-2-yl)pentanoyl)-*N*,*N*-dimethylpiperazine-1-sulfonamide (43b)

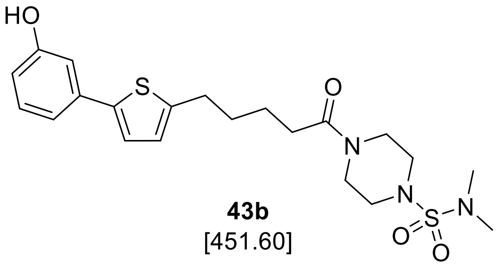

43b
[451.60]

The title compound was synthesised as described in the **general procedure 1A** with **2b** (100 mg, 0.36 mmol, 1.0 eq.), **50** (83.0 mg, 0.36 mmol, 1.0 eq.), EDC•HCl (104 mg, 0.54 mmol, 1.5 eq.), HOBt (73 mg, 0.54 mmol, 1.5 eq.) and NEt_3 (0.15 mL, 1.08 mmol, 3.0 eq.) in DCM (25 mL). The crude product was purified *via* column chromatography (DCM:MeOH 80:1) and recrystallised of EtOAc. 90 mg (0.20 mmol, 56%) of the desired product **43b** as yellow solid was obtained. R_f = 0.08 (DCM:MeOH 80:1). Melting point: 134 °C. ^{1}H-NMR: (DMSO-D$_6$, 400 MHz), δ [ppm] = 9.51 (s, 1H), 7.22 (d, ^{3}J = 3.7 Hz, 1H), 7.17 (t, ^{3}J = 7.8 Hz, 1H), 7.00 (dt, ^{3}J = 7.6 Hz, ^{4}J = 1.6 Hz, 1H), 6.95 (t, ^{4}J = 2.0 Hz, 1H), 6.82 (d, ^{3}J = 3.4 Hz, 1H), 6.67 (dd, ^{3}J = 8.0 Hz, ^{4}J = 2.5 Hz, 1H), 3.49 (s, 4H), 3.16-3.09 (m, 4H), 2.81 (t, ^{3}J = 7.2 Hz, 2H), 2.76 (s, 6H), 2.37 (t, ^{3}J = 7.3 Hz, 2H), 1.66 (quin, ^{3}J = 7.3 Hz, 2H), 1.57 (quin, ^{3}J = 7.1 Hz, 2H). ^{13}C-NMR: (DMSO-D$_6$, 100 MHz), δ [ppm] = 170.7 (1C), 157.8 (1C), 144.5 (1C), 140.9 (1C), 135.2 (1C), 130.1 (1C), 125.7 (1C), 123.1 (1C), 115.8 (1C), 114.3 (1C), 111.7 (1C), 46.3 (1C), 46.0 (1C), 44.7 (1C), 40.7 (1C), 37.8 (2C), 31.9 (1C), 30.7 (1C), 29.3 (1C), 24.1 (1C). IR: ν [cm^{-}

[1]] = 3170 (w), 2945 (w), 2933 (w), 2850 (w), 1620 (s), 1595 (s), 1549 (w), 1476 (m), 1445 (s), 1414 (m), 1372 (w), 1330 (s), 1312 (m), 1291 (s), 1240 (m), 1224 (m), 1198 (m), 1140 (vs), 1111 (m), 1085 (w), 1074 (w), 1056 (w), 984 (vs), 963 (m), 938 (m), 890 (vs), 857 (m), 838 (w), 801 (m), 785 (m), 774 (vs), 747 (vs), 707 (s), 689 (s), 631 (m), 601 (w), 591 (w), 576 (m), 536 (s), 519 (m), 485 (w), 471 (w), 458 (w), 441 (m), 417 (w). MS (ESI+): m/z (%) = 496 (14, $[M+NH_4]^+$), 474 (10, $[M+Na]^+$). HRMS (ESI+) calculated for $C_{21}H_{29}N_3NaO_4S_2$: 474.1492; found: 474.1499.

tert-Butyl 4-(phenylsulfonyl)piperazin-1-carboxylate (51)

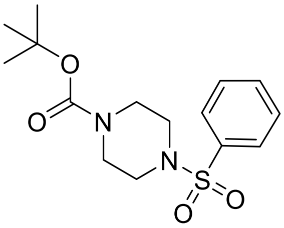

51
[326.41]

The title compound was synthesised as described in the **general procedure 4** with Boc-piperazine (400 mg, 2.15 mmol, 1.0 eq.), phenylsulfonyl chloride (0.3 mL, 2.15 mmol, 1.0 eq.) and NEt_3 (0.9 mL, 6.45 mmol, 3.0 eq.) in 1,4-dioxan (4.5 mL) and H_2O (1.5 mL). The crude product was used without any purification on the next step. 689 mg (2.11 mmol, 98%) of the desired product **51** as pale-yellow solid was obtained. Melting point: 113-115 °C. ^{1}H-NMR: (DMSO-D$_6$, 400 MHz), δ [ppm] = 7.80-7.73 (m, 3H), 7.68-7.58 (m, 2H), 3.39 (t, ^3J = 4.9 Hz, 4H), 2.85 (t, ^3J = 5.0 Hz, 4H), 1.34 (s, 9H). ^{13}C-NMR: (DMSO-D$_6$, 100 MHz), δ [ppm] = 153.4 (1C), 134.8 (1C), 133.3 (1C), 129.4 (2C), 127.5 (2C), 79.3 (1C), 45.7 (4C), 27.9 (3C). MS (ESI+): m/z (%) = 349 (45, $[M+Na]^+$). HRMS (ESI+) calculated for $C_{15}H_{22}NaN_2O_4S$: 349.1192; found: 349.1185.

4-(Phenylsulfonyl)piperazinium chloride (52)

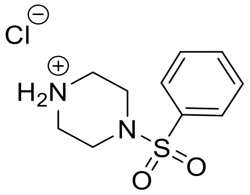

52
[262.75]

The title compound was synthesised as described in the **general procedure 3** with **51** (611 mg, 1.87 mmol, 1.0 eq.) and 4 M HCl in 1,4-dioxan (2 mL) in 1,4-Dioxan (5 mL). 345 mg (1.31 mmol, 70%) of the desired product **52** as colourless solid was obtained. Melting point: 216-218 °C. ^{1}H-NMR: (DMSO-D$_6$, 400 MHz), δ [ppm] = 9.39 (s, 2H), 7.79-7.68 (m, 5H), 3.16 (s, 8H). ^{13}C-NMR: (DMSO-D$_6$, 100 MHz), δ [ppm] = 134.4 (1C), 133.7 (1C), 129.6 (2C), 127.6 (2C), 42.7 (2C), 42.0 (2C). MS (ESI+): m/z (%) = 227 (100, [M-Cl]$^+$). HRMS (ESI+) calculated for $C_{10}H_{15}N_2O_2S$: 227.0849; found: 227.0845.

1-(5-(3-Hydroxyphenyl)thiophene-2-yl)-5-(4-(phenylsulfonyl)piperazine-1-yl)pentane-1,5-dione (44a)

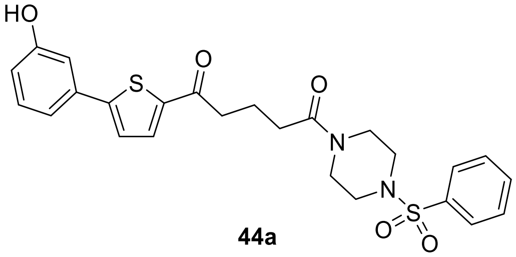

44a
[498.61]

The title compound was synthesised as described in the **general procedure 3** with **2a** (300 mg, 1.03 mmol, 1.0 eq.), **52** (270 mg, 1.03 mmol, 1.0 eq.), NEt_3 (0.4 mL, 3.09 mmol, 3.0 eq.), HOBt (209 mg, 1.55 mmol, 1.5 eq.) and EDC•HCl (296 mg, 1.55 mmol, 1.5 eq.) in DCM (25 mL). The crude product was purified *via* column chromatography (DCM:MeOH 60:1). 489 mg (0.75 mmol, 73%) of the desired product **44a** as colourless solid was obtained. R_f = 0.40 (DCM:MeOH 60:1). Melting point: 174-176 °C. ^{1}H-NMR: (DMSO-D$_6$, 400 MHz), δ [ppm] = 9.71 (s, 1H), 7.87-7.85 (m, 1H), 7.75-7.71 (m, 3H), 7.67-7.63 (m, 2H), 7.53-7.51 (m, 1H), 7.26 (t, ^{3}J = 7.9 Hz, 1H), 7.17 (d, ^{3}J = 7.6 Hz, 1H), 7.10-7.09 (m, 1H), 6.83-6.80 (m, 1H), 3.52 (s, 4H), 2.87-2.94 (m, 6H), 2.33 (t, ^{3}J = 7.2 Hz, 2H), 1.77 (quin, ^{3}J = 7.0 Hz, 2H). ^{13}C-NMR: (DMSO-D$_6$,

100 MHz), δ [ppm] = 192.8 (1C), 170.3 (1C), 158.0 (1C), 151.3 (1C), 142.1 (1C), 134.7 (1C), 134.3 (1C), 133.9 (1C), 133.4 (1C), 130.4 (1C), 129.5 (2C), 127.6 (2C), 124.9 (1C), 116.9 (1C), 116.3 (1C), 112.6 (1C), 46.0 (1C), 45.8 (1C), 44.0 (1C), 40.1 (1C), 37.4 (1C), 21.3 (1C), 19.7 (1C). MS (ESI-): m/z (%) = 497 (100, $[M-H]^-$). HRMS (ESI-) calculated for $C_{25}H_{25}N_2O_5S_2$: 497.1210; found: 497.1196.

5-(5-(3-Hydroxyphenyl)thiophene-2-yl)-1-(4-(phenylsulfonyl)piperazine-1-yl)pentane-1-one (44b)

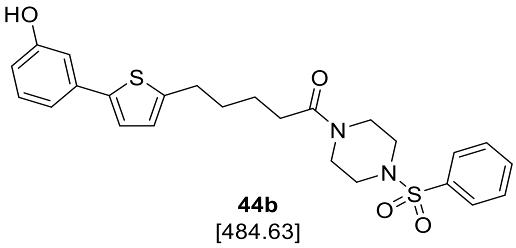

44b
[484.63]

The title compound was synthesised as described in the **general procedure 3** with **2b** (100 mg, 0.36 mmol, 1.0 eq.), **52** (94.0 mg, 0.36 mmol, 1.0 eq.), EDC•HCl (104 mg, 0.54 mmol, 1.5 eq.), HOBt (73 mg, 0.54 mmol, 1.5 eq.) and NEt_3 (0.15 mL, 1.08 mmol, 3.0 eq.) in DCM (25 mL). The crude product was purified *via* column chromatography (DCM:MeOH 80:1) and recrystallised of EtOAc. 55 mg (0.11 mmol, 31%) of the desired product **44b** as colourless solid was obtained. R_f = 0.08 (DCM:MeOH 80:1). Melting point: 173 °C. ^{1}H-NMR: (DMSO-D_6, 400 MHz), δ [ppm] = 9.53 (s, 1H), 7.74-7.72 (m, 3H), 7.66-7.62 (m, 2H), 7.20-7.15 (m, 2H), 7.00 (d, ^3J = 7.1 Hz, 1H), 6.92 (s, 1H), 6.78 (d, ^3J = 3.0 Hz, 1H), 6.67 (d, ^3J = 8.0 Hz, 1H), 3.52 (s, 4H), 2.87 (s, 4H), 2.75 (t, ^3J = 7.2 Hz, 2H), 2.29 (t, ^3J = 7.1 Hz, 2H), 1.59 (quin, ^3J = 7.2 Hz, 2H), 1.49 (quin, ^3J = 7.0 Hz, 2H). ^{13}C-NMR: (DMSO-D_6, 100 MHz), δ [ppm] = 170.6 (1C), 157.8 (1C), 144.5 (1C), 140.9 (1C), 135.2 (1C), 134.7 (1C), 133.4 (1C), 130.1 (1C), 129.5 (2C), 127.6 (2C), 125.6 (1C), 123.1 (1C), 115.8 (1C), 114.3 (1C), 111.7 (1C), 59.8 (1C), 46.1 (1C), 45.8 (1C), 44.1 (1C), 31.7 (1C), 30.6 (1C), 29.2 (1C), 24.0 (1C). IR: ν [cm^{-1}] = 3145 (w), 2943 (w), 2910 (w), 2849 (w), 1611 (m), 1592 (s), 1548 (w), 1447 (s), 1417 (w), 1354 (s), 1330 (m), 1310 (m), 1295 (m), 1277 (m), 1204 (m), 1171 (vs), 1116 (m), 1093 (m), 1054 (w), 1033 (w), 986 (w), 944 (w), 918 (m), 870 (w), 858 (m), 801 (w), 778 (m), 755 (m), 738 (vs), 690 (s), 644 (w), 589 (m), 574 (vs), 532 (m), 503 (w), 448 (m). MS (ESI+): m/z (%) = 502 (44, $[M+NH_4]^+$), 506 (50, $[M+Na]^+$). HRMS (ESI+) calculated for $C_{25}H_{28}N_2NaO_4S_2$: 507.1383; found: 507.1390.

1-(5-(3-Hydroxyphenyl)thiophene-2-yl)-5-morpholinopentane-1,5-dione (45a)

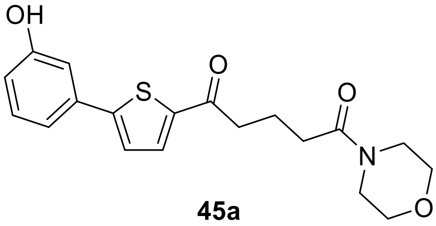

45a
[359.44]

The title compound was synthesised as described in the **general procedure 1A** with **2a** (200 mg, 0.69 mmol, 1.0 eq.), morpholine (0.06 mL, 0.69 mmol, 1.0 eq.), NEt_3 (0.3 mL, 2.07 mmol, 3.0 eq.), HOBt (140 mg, 1.04 mmol, 1.5 eq.) and EDC•HCl (199 mg, 1.04 mmol, 1.5 eq.) in DCM (25 mL). The crude product was purified *via* column chromatography (cyclohexane:EtOAc 1:4). 172 mg (0.48 mmol, 70%) of the desired product **45a** as yellow solid was obtained. R_f = 0.10 (cyclohexane:EtOAc 1:4). Melting point: 208-209 °C. ^{1}H-NMR: (DMSO-D_6, 400 MHz), δ [ppm] = 9.71 (s, 1H), 7.91 (d, ^3J = 3.9 Hz, 1H), 7.55 (d, ^3J = 3.9 Hz, 1H), 7.26 (t, ^3J = 7.8 Hz, 1H), 7.18 (d, ^3J = 7.6 Hz, 1H), 7.11 (s, 1H), 6.81 (dd, ^3J = 8.0 Hz, ^4J = 2.3 Hz, 1H), 3.55-3.52 (m, 4H), 3.43 (s, 4H), 2.99 (t, ^3J = 7.2 Hz, 2H), 2.38 (t, ^3J = 7.3 Hz, 2H), 1.85 (quin, ^3J = 7.3 Hz, 2H). ^{13}C-NMR: (DMSO-D_6, 100 MHz), δ [ppm] = 192.9 (1C), 170.5 (1C), 158.0 (1C), 151.3 (1C), 142.2 (1C), 134.3 (1C), 133.9 (1C), 130.4 (1C), 124.9 (1C), 116.8 (1C), 116.3 (1C), 112.6 (1C), 66.1 (2C), 45.3 (1C), 41.4 (1C), 37.5 (1C), 31.3 (1C), 19.9 (1C, Thiophen-CO-CH_2-$\underline{C}H_2$). IR: ν [cm^{-1}] = 3080 (w), 2917 (m), 1653 (s), 1618 (m), 1582 (s), 1446 (vs), 1361 (m), 1326 (m), 1262 (s), 1220 (s), 1191 (s), 1184 (s), 1105 (vs), 1056 (m), 1028 (s), 999 (m), 958 (m), 907 (m), 864 (s), 836 (vs), 807 (m), 751 (vs), 687 (s), 645 (m), 581 (m), 559 (m), 496 (m), 467 (m). MS (ESI+): m/z (%) = 360 (60, $[M+H]^+$). HRMS (ESI+) calculated for $C_{19}H_{22}NO_4S$: 360.1264; found: 360.1255.

5-(5-(3-Hydroxyphenyl)thiophene-2-yl)-1-morpholinopentane-1-one (45b)

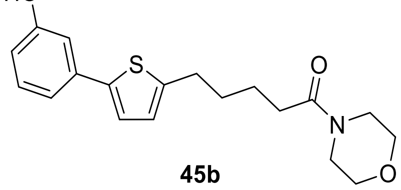

45b
[345.46]

The title compound was synthesised as described in the **general procedure 1A** with **2b** (100 mg, 0.36 mmol, 1.0 eq.), morpholine (0.03 mL, 0.36 mmol, 1.0 eq.), EDC•HCl (104 mg, 0.54 mmol, 1.5 eq.), HOBt (73.0 mg, 0.54 mmol, 1.5 eq.) and NEt_3 (0.15 mL, 1.08 mmol, 3.0 eq.) in DCM (25 mL). The crude product was purified *via* column chromatography (cyclohexane:EtOAc 1:1 → 1:2). 67 mg (0.19 mmol, 53%) of the desired product **45b** as yellow solid was obtained. R_f = 0.12 (cyclohexane:EtOAc 1:1). Melting point: 194 °C. ^{1}H-NMR: (DMSO-D$_6$, 400 MHz), δ [ppm] = 9.50 (s, 1H), 7.22 (d, ^3J = 3.6 Hz, 1H), 7.17 (t, ^3J = 7.9 Hz, 1H), 7.02-6.99 (m, 1H), 6.95 (t, ^3J = 1.9 Hz, 1H), 6.82 (d, ^3J = 3.6 Hz, 1H), 6.67 (dd, ^3J = 8.1 Hz, ^4J = 2.4 Hz, 1H), 3.53 (brs, 4H), 3.43-3.41 (m, 4H), 2.80 (t, ^3J = 7.2 Hz, 2H), 2.34 (t, ^3J = 7.3 Hz, 2H), 1.66 (quin., ^3J = 7.3 Hz, 2H), 1.56 (quin, ^3J = 7.3 Hz, 2H). ^{13}C-NMR: (DMSO-D$_6$, 100 MHz), δ [ppm] = 170.7 (1C), 157.8 (1C), 144.5 (1C), 140.8 (1C), 135.2 (1C), 130.0 (1C), 125.6 (1C), 123.0 (1C), 115.8 (1C), 114.3 (1C), 111.7 (1C), 66.1 (2C), 45.4 (1C), 41.4 (1C), 31.7 (1C), 30.6 (1C), 29.2 (1C), 24.0 (1C). IR: ν [cm^{-1}] = 3082 (w), 3015 (w), 2945 (m), 2915 (m), 2865 (m), 1615 (m), 1588 (s), 1550 (m), 1516 (m), 1469 (m), 1445 (vs), 1413 (m), 1364 (m), 1295 (m), 1271 (m), 1242 (s), 1226 (s), 1216 (s), 1194 (s), 1166 (m), 1141 (m), 1107 (vs), 1086 (m), 1071 (m), 1054 (m), 1029 (m), 1014 (m), 986 (m), 957 (m), 911 (m), 881 (w), 865 (m), 847 (s), 800 (m), 777 (vs), 758 (s), 688 (m), 642 (m), 624 (w), 581 (m), 560 (m), 533 (w), 509 (w), 495 (m), 471 (m), 449 (m). MS (ESI+): m/z (%) = 346 (100, $[M+H]^+$), 363 (13, $[M+NH_4]^+$), 368 (30, $[M+Na]^+$). HRMS (ESI+) calculated for $C_{19}H_{23}NO_3S$: 346.1471; found: 346.1468.

1-(5-(3-Hydroxyphenyl)thiophene-2-yl)-5-thiomorpholinopentane-1,5-dione (46a)

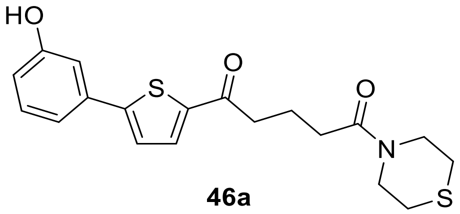

46a
[375.50]

The title compound was synthesised as described in the **general procedure 1A** with **2a** (200 mg, 0.69 mmol, 1.0 eq.), thiomorpholine (0.1 mL, 0.69 mmol, 1.0 eq.), EDC•HCl (199 mg, 1.04 mmol, 1.5 eq.), HOBt (141 mg, 1.04 mmol, 1.5 eq.) and NEt_3 (0.3 mL, 2.07 mmol, 3.0 eq.) in DCM (25 mL). The crude product was purified *via* column chromatography (cyclohexane:EtOAc 1:2) and recrystallised from

cyclohexane/EtOAc/MeOH. 129 mg (0.34 mmol, 49%) of the desired product **46a** as colourless solid was obtained. R_f = 0.26 (cyclohexane:EtOAc 1:2). Melting point: 182 °C. ^{1}H-NMR: (DMSO-D$_6$, 500 MHz), δ [ppm] = 9.71 (s, 1H), 7.91 (d, ^3J = 4.0 Hz, 1H), 7.55 (d, ^3J = 4.0 Hz, 1H), 7.26 (t, ^3J = 7.9 Hz, 1H), 7.20-7.18 (m, 1H), 7.11 (t, ^3J = 1.9 Hz, 1H), 6.83-6.81 (m, 1H), 3.73-3.68 (m, 4H), 2.99 (t, ^3J = 7.3 Hz, 2H), 2.61-2.52 (m, 4H),2.39 (t, ^3J = 7.3 Hz, 2H), 1.85 (quin, ^3J = 7.2 Hz, 2H). ^{13}C-NMR: (DMSO-D$_6$, 125 MHz), δ [ppm] = 192.8 (1C), 170.2 (1C), 157.9 (1C), 151.3 (1C), 142.2 (1C), 134.2 (1C), 133.9 (1C), 130.4 (1C), 124.8 (1C), 116.8 (1C), 116.3 (1C), 112.5 (1C), 47.6 (1C), 43.6 (1C), 37.5 (1C), 31.5 (1C), 27.0 (1C), 26.5 (1C),19.9 (1C). IR: ν [cm^{-1}] =3182 (m), 3099 (w), 2956 (w), 2916 (w), 1650 (vs), 1617 (vs), 1594 (s), 1537 (w), 1446 (vs), 1417 (m), 1372 (m), 1337 (m), 1302 (m), 1289 (m), 1265 (m), 1250 (m), 1225 (s), 1204 (w), 1189 (s), 1079 (w), 1067 (m), 1052 (m), 1024 (m), 994 (w), 959 (m), 935 (m), 868 (m), 843 (m), 809 (m), 779 (m), 769 (m), 747 (m), 704 (m), 684 (s), 665 (m), 645 (w), 585 (w), 574 (w), 524 (m), 502 (w), 471 (w), 461 (w), 432 (m), 410 (w). MS (ESI+): m/z (%) = 376 (40, [M+H]$^+$), 398 (20, [M+Na]$^+$). HRMS (ESI+) calculated for $C_{19}H_{22}NO_3S_2$: 376.1036; found: 376.1038.

5-(5-(3-Hydroxyphenyl)thiophene-2-yl)-1-thiomorpholinopentane-1-one (46b)

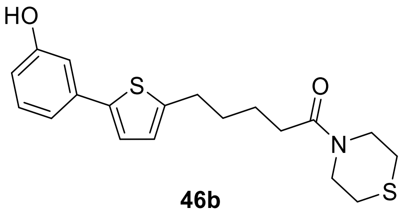

46b
[361.52]

As described in the **general procedure 1A** with **2b** (200 mg, 0.72 mmol, 1.0 eq.), morpholine (0.07 mL, 0.72 mmol, 1.0 eq.), EDC•HCl (208 mg, 1.08 mmol, 1.5 eq.), HOBt (146 mg, 1.08 mmol, 1.5 eq.) and NEt_3 (0.30 mL, 2.16 mmol, 3.0 eq.) in DCM (25 mL). The crude product was purified *via* column chromatography (cyclohexane:EtOAc 1:2). 222 mg (0.61 mmol, 85%) of the desired product **46b** as yellow solid was obtained. R_f = 0.31 (cyclohexane:EtOAc 1:2). Melting point: 163 °C. ^{1}H-NMR: (DMSO-D$_6$, 400 MHz), δ [ppm] = 9.49 (s, 1H), 7.22 (d, ^3J = 3.6 Hz, 1H), 7.17 (t, ^3J = 7.9 Hz, 1H), 7.01 (d, ^3J = 7.7 Hz, 1H), 6.95 (t, ^3J = 1.8 Hz, 1H), 6.82 (d, ^3J = 3.6 Hz, 1H), 6.67 (ddd, ^3J = 8.1 Hz, ^4J = 2.3 Hz, ^4J = 0.8 Hz, 1H), 3.68 (brs, 4H), 2.80 (t, ^3J = 7.3 Hz, 2H), 2.58 (brs, 4H), 2.34 (t, ^3J = 7.2 Hz, 2H), 1.65 (quin.,

^3J = 7.5 Hz, 2H), 1.56 (quin, ^3J = 7.6 Hz, 2H). ^{13}C-NMR: (DMSO-D$_6$, 100 MHz), δ [ppm] = 170.5 (1C), 157.8 (1C), 144.6 (1C), 140.9 (1C), 135.2 (1C), 130.1 (1C), 125.7 (1C), 123.1 (1C), 115.9 (1C), 114.3 (1C), 111.7 (1C), 47.7 (1C), 43.6 (1C), 32.0 (1C), 30.7 (1C), 29.3 (1C), 27.1 (1C), 26.6 (1C), 24.2 (1C). IR: ν [cm^{-1}] = 3145 (w), 3091 (w), 2948 (w), 2917 (w), 2865 (w), 1616 (m), 1591 (s), 1549 (w), 1514 (w), 1472 (m), 1444 (s), 1412 (m), 1366 (w), 1305 (w), 1294 (m), 1283 (w), 1248 (m), 1226 (m), 1212 (w), 1193 (s), 1165 (w), 1084 (w), 1029 (w), 986 (w), 954 (w), 911 (m), 866 (w), 855 (w), 801 (w), 777 (s), 755 (m), 686 (m), 659 (w), 622 (w), 533 (w), 497 (w), 445 (w). MS (ESI+): m/z (%) = 362 (70, [M+H]$^+$), 384 (5, [M+Na]$^+$). HRMS (ESI+) calculated for $C_{19}H_{23}NO_2S_2$: 362.1243; found: 362.1248 and calculated for $C_{19}H_{23}NNaO_2S_2$: 384.1062; found: 384.1067.
